# TNEA Regulates Hippocampal Oscillation by Improving Inhibitory Synaptic Plasticity to Ameliorates Cognitive Impairment in Alzheimer's Disease

**DOI:** 10.1002/advs.202510885

**Published:** 2025-11-11

**Authors:** Zhongzhao Guo, Hong Ni, Yu Lu, Zhengyu Cui, Yixing Wang, Zilu Zhu, Xinyu Wei, Chenyi Xia, Ming Xu, Lixia Du, Yufang Yang, Shi Shu, Ke Wang, Zhifei Wang, Chunlei Shan, Deheng Wang

**Affiliations:** ^1^ Rehabilitation Center Tongren Hospital Shanghai Jiao Tong University School of Medicine Shanghai 200336 China; ^2^ School of Integrative Medicine Shanghai University of Traditional Chinese Medicine Shanghai 201203 China; ^3^ Yuanshen Rehabilitation Institute Shanghai Jiao Tong University School of Medicine Shanghai 200025 China; ^4^ Yueyang Hospital of Integrated Traditional Chinese and Western Medicine Shanghai University of Traditional Chinese Medicine Shanghai 201203 China; ^5^ Engineering Research Center of Traditional Chinese Medicine Intelligent Rehabilitation Shanghai University of Traditional Chinese Medicine Shanghai 201203 China; ^6^ Department of Traditional Chinese Medicine Shanghai East Hospital Tongji University School of Medicine Shanghai 201203 China; ^7^ Shanghai Key Laboratory of Flexible Medical Robotics Tongren Hospital Institute of Medical Robotics Shanghai JiaoTong University Shanghai 200336 China

**Keywords:** alzheimer's disease, CA1, in vivo recording, oscillation, three‐needle electroacupuncture

## Abstract

Three‐needle electroacupuncture (TNEA) has demonstrated efficacy in improving cognitive function in both Alzheimer's disease (AD) model animals and patients, although its underlying mechanism remains unclear. Here this work investigates the potential connection between cognitive‐enhancing effect and TNEA in 5×familial Alzheimer disease（5xFAD) mice model, a model characterized by Amyloid‐beta (Aβ) pathology. This work finds alterations in gamma/theta oscillations and deficits in inhibitory monosynaptic transmission in the hippocampal CA1 region of AD. Parvalbumin‐positive (PV^+^) interneurons are crucial for generating gamma oscillations and modulating theta oscillation, thereby maintaining the excitation‐inhibition (E/I) balance in local neural circuits. In 5xFAD mice, TNEA modulated PV^+^ interneuron function, enhancing gamma oscillations during quiescent states. Furthermore, during the novel object recognition test (NORT), TNEA increased theta oscillation power by strengthening presynaptic inhibitory interneurons involved in monosynaptic connections. Collectively, these findings suggest TNEA is a viable minimally invasive treatment approach for AD.

## Graphical abstract

TNEA improves cognition in 5xFAD mice by modulating parvalbumin‐positive interneurons in hippocampal CA1. TNEA enhances gamma oscillations during rest and theta power during novel object recognition, restoring excitation/inhibition balance. These findings support TNEA as a minimally invasive therapy for Alzheimer's disease.

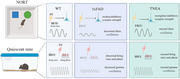

## 1 Introduction

Alzheimer's disease (AD)is the most common neurodegenerative disorder, characterized by memory loss and cognitive dysfunction.^[^ ^1^ ^]^ AD is characterized by senile plaques formed by the abnormal deposition of Amyloid‐beta (Aβ), neurofibrillary tangles (NFTs),^[^ ^2^ ^]^ and microglia activation.^[^ ^3^ ^]^ The cortex and hippocampus exhibit some of the earliest damage associated with AD. Recent studies indicate that microglia and neuroinflammation play a critical role in AD.^[^ ^3^ ^]^ In patients with AD^[^ ^4^ ^]^ and in mouse model of AD,^[^ ^5^, ^6^ ^]^ alterations in oscillations within the gamma and theta frequency band associated hippocampal CA1 network function have been observed.^[^ ^7^, ^8^ ^]^ Reductions in gamma and theta oscillations have been documented in vitro in the amyloid precursor protein (APP) mouse model,^[^ ^5^ ^]^ and similarly in vivo inTriple‐transgenic Alzheimer's disease (3xTg AD) mice and 5×familial Alzheimer disease (5xFAD) mice.^[^ ^9^ ^]^ The dysfunction of neuronal oscillations^[^ ^10^ ^]^ can be detected even before the formation Aβ plaques.^[^ ^11^, ^12^ ^]^ The mechanism by which this occurs may be that soluble β Aβ or its fibrillar form could affect synaptic and neuronal function.^[^ ^10^, ^13^, ^14^ ^]^

Both gamma and theta alterations depend on the rapid firing of hippocampal CA1Parvalbumin‐positive ( PV^+^) interneurons.^[^ ^15^, ^16^ ^]^ Inhibitory GABAergic interneurons, particularly PV^+^ cells in the hippocampus, are essential for generating gamma oscillations.^[^ ^15^, ^17^, ^18^ ^]^ Meanwhile, PV^+^ cells in the hippocampal CA1 region receive input from medial septum (MS) PV^+^ cells and play a crucial role in regulating theta band oscillations in the hippocampal CA1.^[^ ^19^ ^]^ Furthermore, optogenetic activation of PV^+^ interneurons has shown the capability to stimulate gamma oscillations and alleviate pathological features associated with AD.^[^ ^20^ ^]^ PV^+^ cells are vital for maintaining the excitatory and inhibitory balance of local circuits by preserving synaptic connections.^[^ ^21^ ^]^

Synaptic dysfunction is considered a central factor in the development of AD. In 5xFAD mice, post‐mortem analysis of pyramidal cells shows a reduction in dendritic spine density. Additionally, various AD mouse models demonstrate an increased vulnerability of interneurons.^[^ ^22^, ^23^, ^24^, ^25^ ^]^ Synaptic loss is one of the strongest correlates of cognitive deficits associated with the disease,^[^ ^26^, ^27^ ^]^ and synaptic transmission correlates with memory deficits.^[^ ^23^, ^24^, ^28^, ^29^ ^]^ 5xFAD mice are defective in excitatory and inhibitory synaptic transmission, It is thought that these synaptic deficits result in reduced neural activity as excitatory and inhibitory interactions are important for generating theta or gamma oscillations.^[^ ^30^, ^31^, ^32^, ^33^ ^]^ However, clinical trials that targeted Aβ, tau pathologies, and neuroinflammation have failed to demonstrate the desired effects on cognitive impairments in AD patients,^[^ ^34^, ^35^ ^]^ meaning that new targets are urgently required.

Electroacupuncture(EA) therapy, which is based on the meridian and acupoints plays a significant role in the clinical treatment of AD patients. It has the potential to prevent the onset of AD, slow the progression of the disease, and stabilize or enhance symptoms.^[^ ^36^, ^37^ ^]^ Numerous laboratory experiments have suggested that EA can reduce neuroinflammatory responses in the hippocampus,^[^ ^38^ ^]^ promote the degradation of Aβ,^[^ ^39^ ^]^ and improves spatial learning memory^[^ ^40^ ^]^ in various AD mouse models. Furthermore, EA at GV24 and bilateral GB13, which is called three‐needle electroacupuncture (TNEA), has previously been shown to improve cognitive impairment in both AD patients^[^ ^41^ ^]^ and mouse models,^[^ ^42^, ^43^ ^]^ however, the underlying mechanisms are still under investigation.

Consequently, to explore the underlying mechanisms of TNEA to treat AD, behavioral experiments were performed to examine the cognitive ability of 5xFAD mice and a series of molecular biology experiments were conducted in order to understand the internal mechanisms. Additionally, neuronal activity change in hippocampal CA1 was performed by Tetrode in‐vivo recording. Behaviorally, TNEA significantly improved the spatial learning and memory of 5xFAD mice. Molecularly, TNEA reduced Aβ deposition, inhibited microglial activation, increased the number of PV^+^ interneurons, and activated brain derived neurotrophic factor (BDNF) by the cyclic adenosine monophosphate (cAMP)‐Protein kinase A (PKA) cAMP–(response element‐binding protein) CREB‐BDNF pathway in the hippocampal CA1. Electrophysiologically, TNEA enhanced gamma oscillation in the hippocampal CA1 region of 5xFAD by activating PV^+^ neurons during quiescent state and improved theta oscillation by activating presynaptic PV^+^ neurons in inhibitory synaptic connections during novel object recognition test (NORT). Importantly, all of these effects of TNEA, except for Aβ deposition and microglial morphological transition, disappeared after the chemogenetic inhibition of PV^+^ interneurons.″ In summary, we demonstrated, for the first time, that TNEA enhanced cognitive functions by modulating theta and gamma oscillations through the activation of PV^+^ interneurons or PV^+^ interneurons that are involved in inhibitory monosynaptic connections.

## 2 Results

### 2.1 TNEA Improved Cognitive Function in 5xFAD Mice

**Figure 1:**
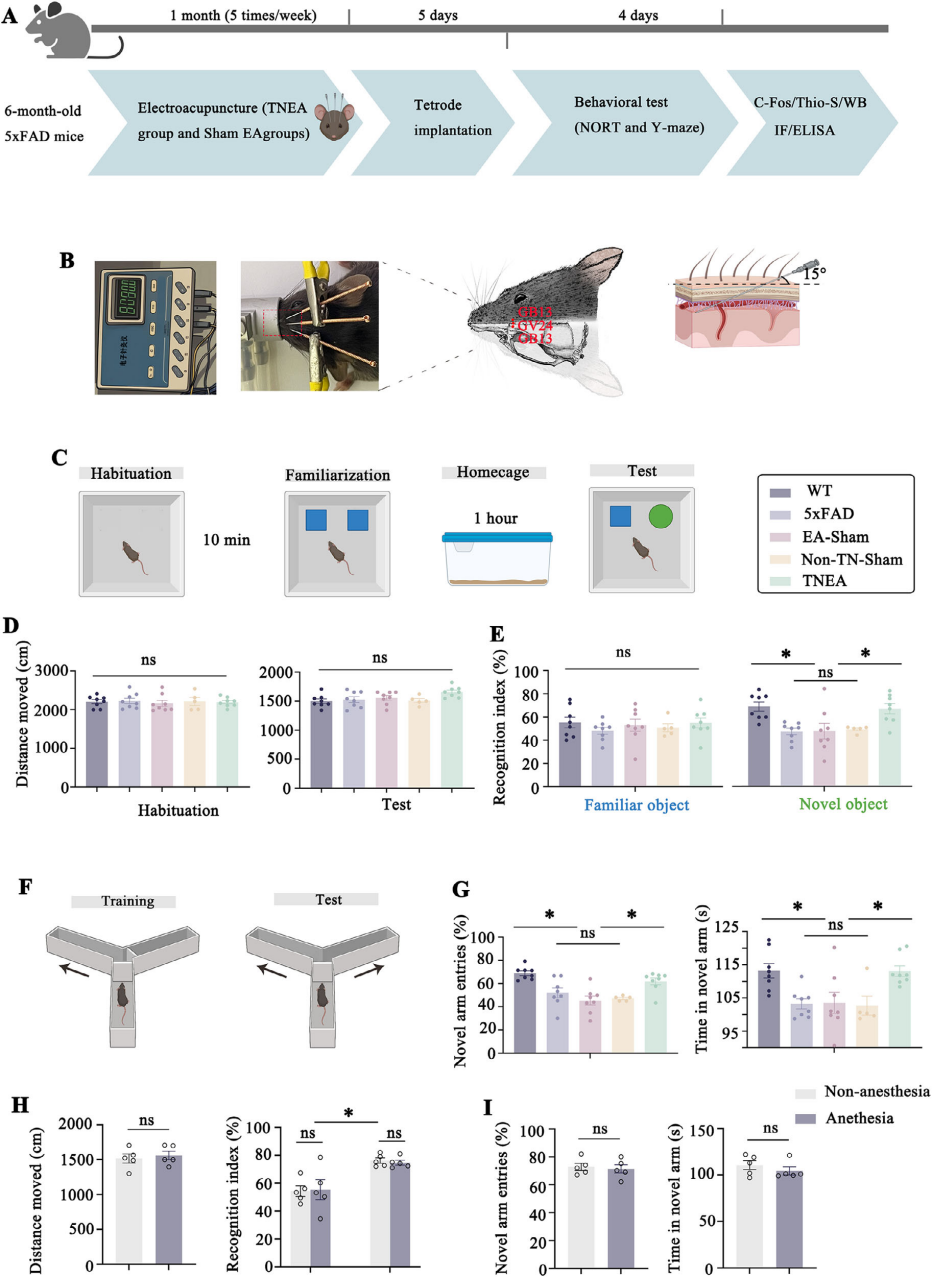
TNEA improved the cognitive function in 5xFAD mice. A) Experimental procedures. NORT: The novel object recognition test; WB: Western blotting; IF: Immunofluorescence. B) The position of GV24 and bilateral GB13 with TNEA treatment, and the anatomical structure diagram of acupuncture points. C) Schematic representation of NORT experimental procedures. D) Distance traveled during the habituation and test sessions during NORT. (n = 8 per group for WT, 5xFAD, EA‐Sham, and TNEA groups. n= 5 per group for Non‐TN‐Sham group). E) The recognition index during the choice phase of NORT (n = 8 per group for WT, 5xFAD, EA‐Sham, and TNEA groups. n = 5 per group for Non‐TN‐Sham group). Statistical significance was set at *p < 0.05, one‐way ANOVA with Tukey's multiple comparisons test, ns: not significant. F) Schematic representation of Y‐maze experimental procedures. G) The percentage of noval arm entries (left) and the time spent in noval arm(right) in the Y‐maze test. (n = 8 per group for WT, 5xFAD, EA‐Sham, and TNEA groups. n= 5 for Non‐TN‐Sham group). H) Distance traveled during the habituation and test sessions during NORT. (n = 5 per group) I) The percentage of noval arm entries (left) and the time spent in noval arm(right) in the Y‐maze test. (n = 5 per group) Statistical significance was set at *p < 0.05, two‐tailed unpaired t‐test, ns: not significant. All data are expressed as mean ± s.e.m.

To investigate the effects of TNEA in AD, 6‐month‐old 5xFAD mice were treated with TNEA, Sham‐EA or non‐three needle (TN)‐acupoit for 4 weeks (**Figure** **1**A,B). Y‐maze behavioral test (Y‐maze) and NORT were used to evaluate spatial learning memory and recognition memory separately (Figure 1C, F). There are 3 stages in NORT: the stage of habituation, the stage of familiarization, and test phase (Figure 1C). First we tested the locomotor ability of 5 groups of mice, and found that there was no significant difference in the distance traveled by the mice in the stage of habituation, and the stage of test (Figure 1D), suggesting that the locomotor ability of 5 groups were the same. Then we discovered the recognition index (RI) of 5xFAD mice decreased obviously during the test phase, while they were significantly improved in the TNEA but not EA‐Sham group or non TN acupoit group (Figure 1E). When exposed to Y‐maze, 5xFAD mice entered the novel arm less frequently than wild type (WT) mice; however, TNEA treatment significantly increased the number of novel arm entries in 5xFAD mice, whereas no differences were found with EA‐Sham group or non‐TN‐group (Figure 1G left). Meanwhile, 5xFAD mice treated with TNEA but not with EA‐Sham group or non‐TN‐group spent significantly more time in the novel arm (Figure 1G right). Consistent with previous reports suggesting that prolonged or repeated exposure to anesthesia may induce cognitive impairment in experimental animals, we sought to evaluate whether the specific anesthetic regimen used in this study (15 min per session, 5 sessions per week, for 4 weeks) could potentially affect cognitive function in mice. An anesthesia control group, which received the same frequency and duration of anesthetic administration as the TNEA acupuncture group but without the intervention, was compared to a non‐anesthesia control group in NORT and Y‐maze test. Results demonstrated that under the current anesthetic protocol, no significant differences in cognitive performance were observed between groups(Figure 1H,I). TNEA improved the cognitive function of the 5xFAD mouse.

### 2.2 TNEA Reduced the Deposition of Aβ and Promoted Microglial Phenotype Transition in the HIPPocampal CA1 of 5xFAD Mice

**Figure 2:**
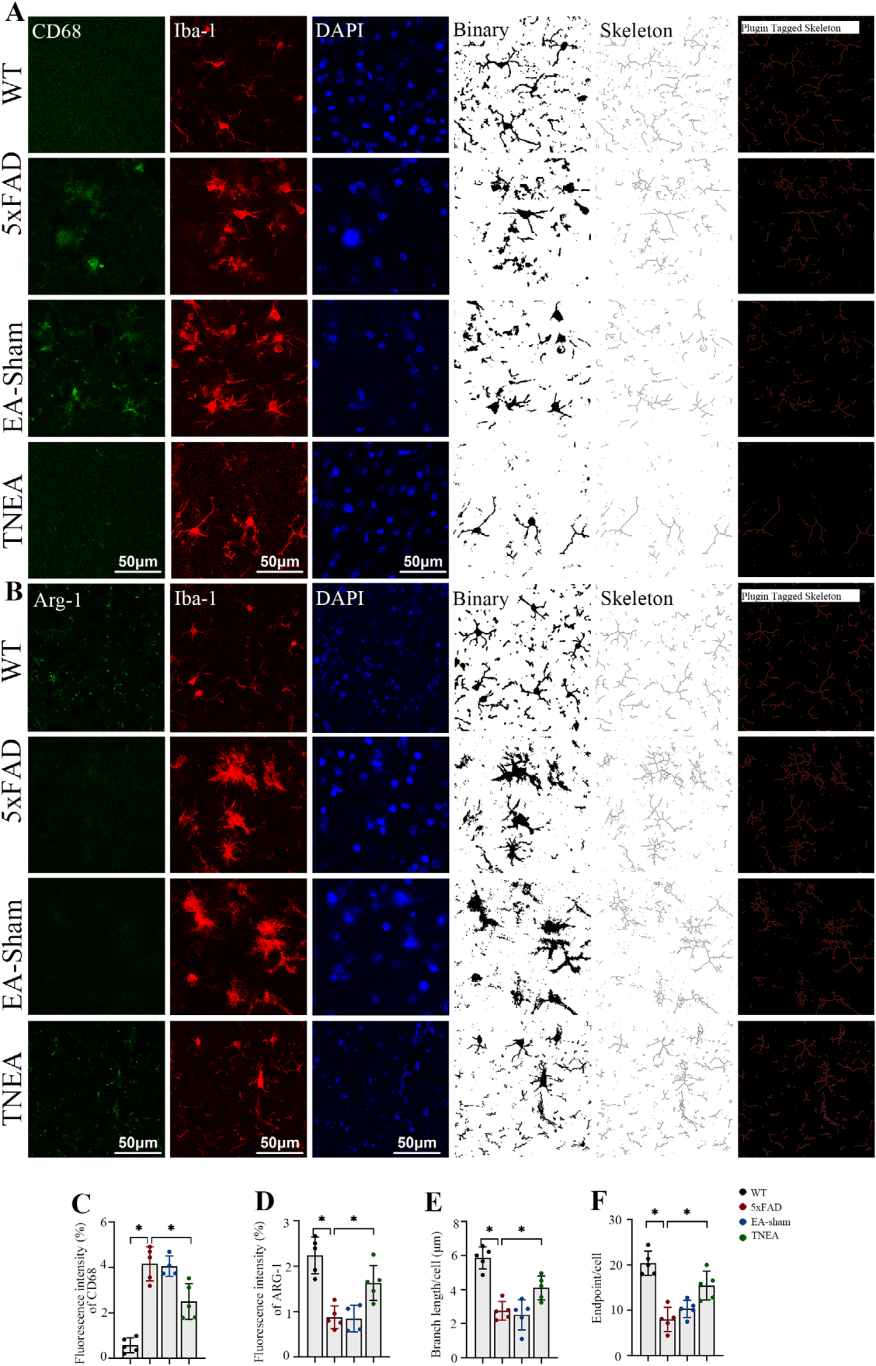
TNEA reduced microglial activation, and promoted microglial phenotype transition in the hippocampal CA1 of 5xFAD mice. A) Representative double immunofluorescence micrographs of CD68 (green) and Iba‐1 (red) in the hippocampal CA1 of different groups and skeleton analysis of Iba‐1^+^ microglial morphologies. B) Representative double immunofluorescence micrographs of Arg‐1 (green) and Iba‐1 (red) in the hippocampal CA1 of different groups and skeleton analysis of Iba‐1^+^ microglial morphologies. C) Quantification of CD68^+^ fluorescence intensity. (n = 5 mice per group) D) Quantification of Arg‐1^+^ fluorescence intensity. (n = 5 mice per group) E) Quantitative analysis of microglia process length/cell. (n = 5 mice per group) F) Quantitative analysis of microglia endpoints/cells in the hippocampus of different groups. (n = 5 mice per group). Statistical significance was set at *p < 0.05, one‐way ANOVA with Tukey's multiple comparisons test, ns: not significant All data are expressed as mean ± s.e.m.

We recorded local field potentials (LFPs) in brain regions typically affected by AD pathology—including the cortex, dentate gyrus (DG), and CA1 area of the hippocampus—following TNEA. The results demonstrated that TNEA significantly enhanced oscillatory power in the CA1 region. In contrast, no significant changes in oscillatory power were observed in either the cortex or the DG region (Figure S1A,B, Supporting Information, Figure 3A–D). We then compared thioflavin‐S (Thio‐S) positive plaques between the groups at the cortex CA1 and DG regions to detect the potential effect of TNEA on the Aβ burden. This analysis demonstrated that the 5xFAD mice exhibited apparent Aβ deposition. In cortex and CA1 region, a significant decrease in plaque deposits was observed in the TNEA treatment group, while no changes were observed in the EA‐shame group (Figure S1C,D, Supporting Information). Meanwhile, the TNEA group exhibited increased c‐Fos positive cells in hippocampal CA1 versus other groups (Figure S1E,F, Supporting Information). In the immunofluorescence (IF) of ionized calcium‐binding adapter molecule 1(Iba‐1) (red) and cluster of differentiation 68 (CD68)/ arginase‐1 (Arg‐1) (green), we found a significant increase in CD68 expression, but a decrease in Arg1 in the hippocampus of 5xFAD mice. The change, however, was reverted by TNEA but not EA‐Sham (**Figure** **2**A–D). It was also observed that a significant number of microglia were activated in the CA1 region of the hippocampus as compared to the WT group, which showed broader cell bodies and simpler structures with fewer branches (Figure 2A,B). Next, we calculated the total branch length and the number of branching endpoints on each cell based on the statistical method applied in previous study^[^ ^44^ ^]^ to identify the changes in microglial morphology. Our results showed that the total branch length and endpoints of the microglia decreased in the 5xFAD ones which would be rescued by the TNEA treatment (Figure 2E,F). No changes in microglial morphology were detected in the EA‐sham group compared to the 5xFAD group (Figure 2E,F). TNEA primarily acts on the CA1 region of the hippocampus, improving oscillations in the CA1 area, alleviating Aβ plaques, and inhibiting microglial activation.″

### 2.3 TNEA Enhanced the High Gamma Oscillations in 5xFAD Mice By Activating Interneurons in the Hippocampal CA1 During Quiescent State

**Figure 3:**
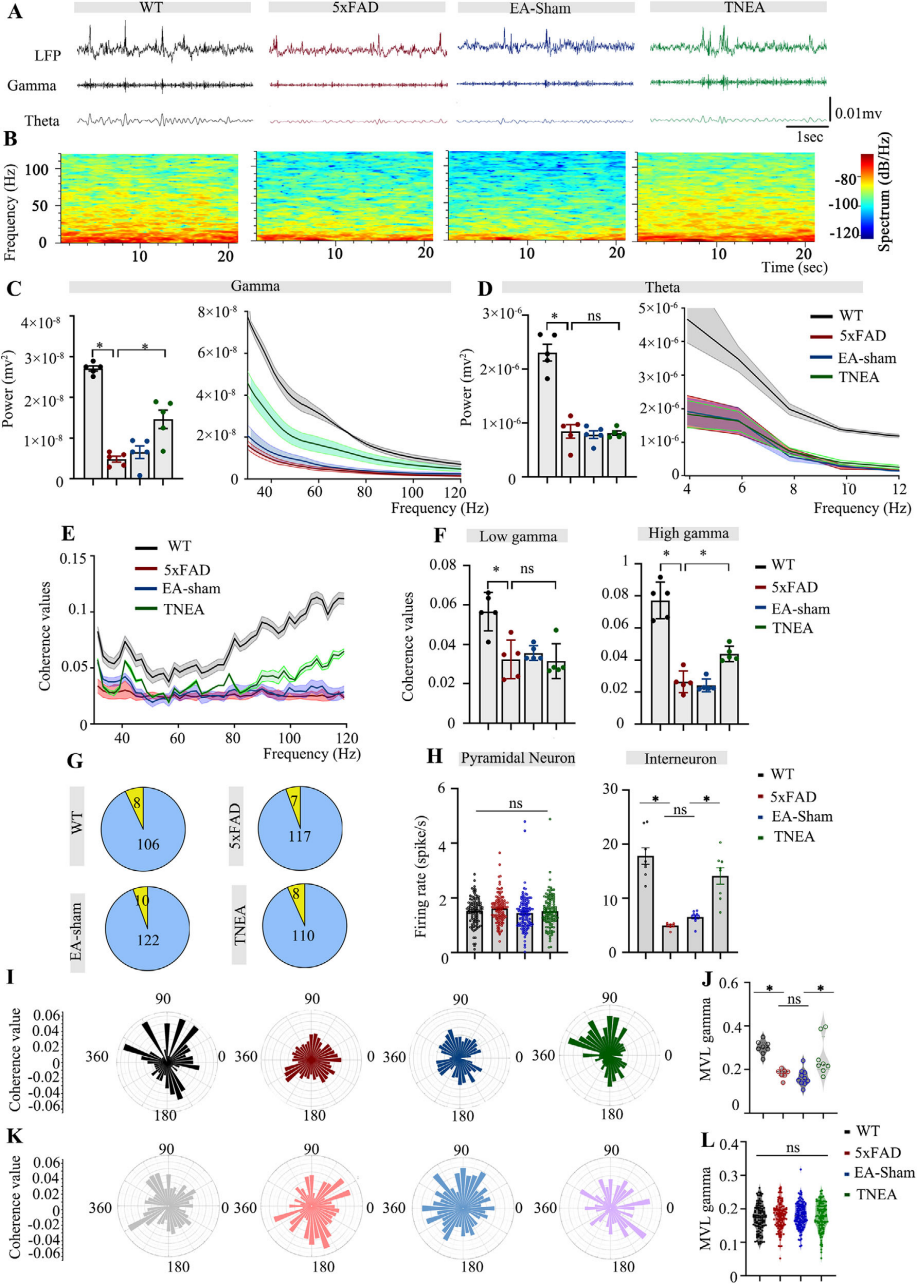
TNEA enhanced the gamma oscillations in 5xFAD mice by activating interneurons in the hippocampal CA1 during quiescent state. A) Representative 1D view of LFP filtered gamma and theta oscillation from 4 groups. B) Representative power spectrogram of broad‐band oscillation from 4 groups. C) Power spectral density (PSD) of gamma oscillations from 4 groups. D) PSD of theta oscillation from 4 groups. E) Coherence values of gamma and theta oscillation of 4 groups. F) Coherence values of low gamma (left), high gamma (Right) and theta oscillation of 4 groups. (n = 5 per group). G) The number of putative pyramidal neurons and putative interneurons recorded in hippocampal CA1 of 4 groups. (H) The firing rate of putative pyramidal neurons (left) (n = 106 for WT, n = 117 for 5xFAD, n = 122 for EA‐Sham, n = 110 for TNEA) and putative interneurons (right), (n = 8 for WT, n = 7 for 5xFAD, n = 10 for EA‐Sham, n = 8 for TNEA). I) Representative polar plots of putative interneurons’ spikes distributions along the gamma phase. J) MVL of 4 groups (n = 8 for WT, n = 7 for 5xFAD, n = 10 for EA‐Sham, n = 8 for TNEA). K) Representative polar plots of putative pyramidal neurons’ spikes distributions along the gamma phase. L) MVL of 4 groups (n = 106 for WT, n = 117 for 5xFAD, n = 122 for EA‐Sham, n = 110 for TNEA). Statistical significance was set at *p < 0.05, one‐way ANOVA with Tukey's multiple comparisons test. All data are expressed as mean ± s.e.m.

In order to assess the neural activities of hippocampal CA1 neurons, we performed LFP in vivo recording. The gamma oscillations are relatively high frequency of up to 120 Hz while the theta oscillations are slow waves between 4 to 8 Hz (**Figure** **3**A,B). To address the contribution of TNEA on gamma and theta oscillations in the hippocampal CA1, the power of gamma and theta bands were recorded. The results showed that both of them were decreased significantly in 5xFAD mice. A partial restoration of gamma oscillations was observed in the TNEA group while no effects on theta oscillations. No significant changes were detected in the EA‐sham group (Figure 3C,D). Then, to explore the relationship between gamma and theta oscillations, we calculated the coherence value^[^ ^45^ ^]^ and noticed that TNEA significantly upregulated in terms of the impaired coherence value associated with high gamma (90–120Hz) and theta oscillations, although not low gamma (30–90Hz) in 5xFAD mice (Figure 3E,F). However, compared to the EA‐sham group, no differences were observed. In addition, since gamma oscillations were modulated by the phase of theta oscillations,^[^ ^45^ ^]^ we sorted gamma power by theta phase and discovered that 5xFAD mice displayed decreased phase‐amplitude coupling of gamma oscillations. Interestingly, this phase‐amplitude coupling dysfunction was attenuated by TNEA but not the EA‐sham (Figure S2A,B, Supporting Information). The spike of a single neuron can cause oscillations at different LFP broad‐band frequencies, and the distribution of the oscillations depends on the type of activated cells. So, we wondered whether the decrease in gamma oscillation power in the 5xFAD group of mice was related to the firing of different kinds of neurons. Total 475 well‐isolated neurons in the hippocampal CA1 were recorded. Based on the electrophysiological firing criteria,^[^ ^46^, ^47^ ^]^ we classified 8/114, 7/124, 10/122, and 8/118 recorded neurons as putative interneurons in WT, 5xFAD, EA‐sham, and TNEA groups, respectively (Figure 3G). We observed the 5xFAD mice showed significant lower firing rates of putative interneurons, but no difference was noticed in the firing rate of pyramidal neurons among the groups (Figure 3H left). Unexpectedly, the inhibition of putative interneurons in 5xFAD mice were reactivated by TNEA treatment (Figure 3H right). We wondered whether TNEA modulated gamma oscillations by improving interneuron's functions. Spike‐gamma coherence was reduced in 5xFAD interneurons but rescued by TNEA treatment. No changes were noted between EA‐sham and 5xFAD mice (Figure S2C,D, Supporting Information). To calculated the phase distribution of spike over gamma oscillation, the mean vector length (MVL)^[^ ^48^ ^]^ were calculated. Relative to EA‐sham, TNEA significantly increased MVL magnitude in 5xFAD mice (Figure 3I,J). No such difference was observed in pyramidal neurons (Figure 3K,L). TNEA primarily improves high gamma oscillations mostly through interneurons.

### 2.4 TNEA Modified the Theta Oscillations in the Hippocampal CA1 of 5xFAD Mice During NORT

We recorded the LFP signals of three groups of mice during four stages. Baseline1 represents the recorded LFP signals during the habituation stage, while Baseline2 represents the LFP signals when the three groups of mice explored zones other than the novel and familiar object zones.

**Figure 4:**
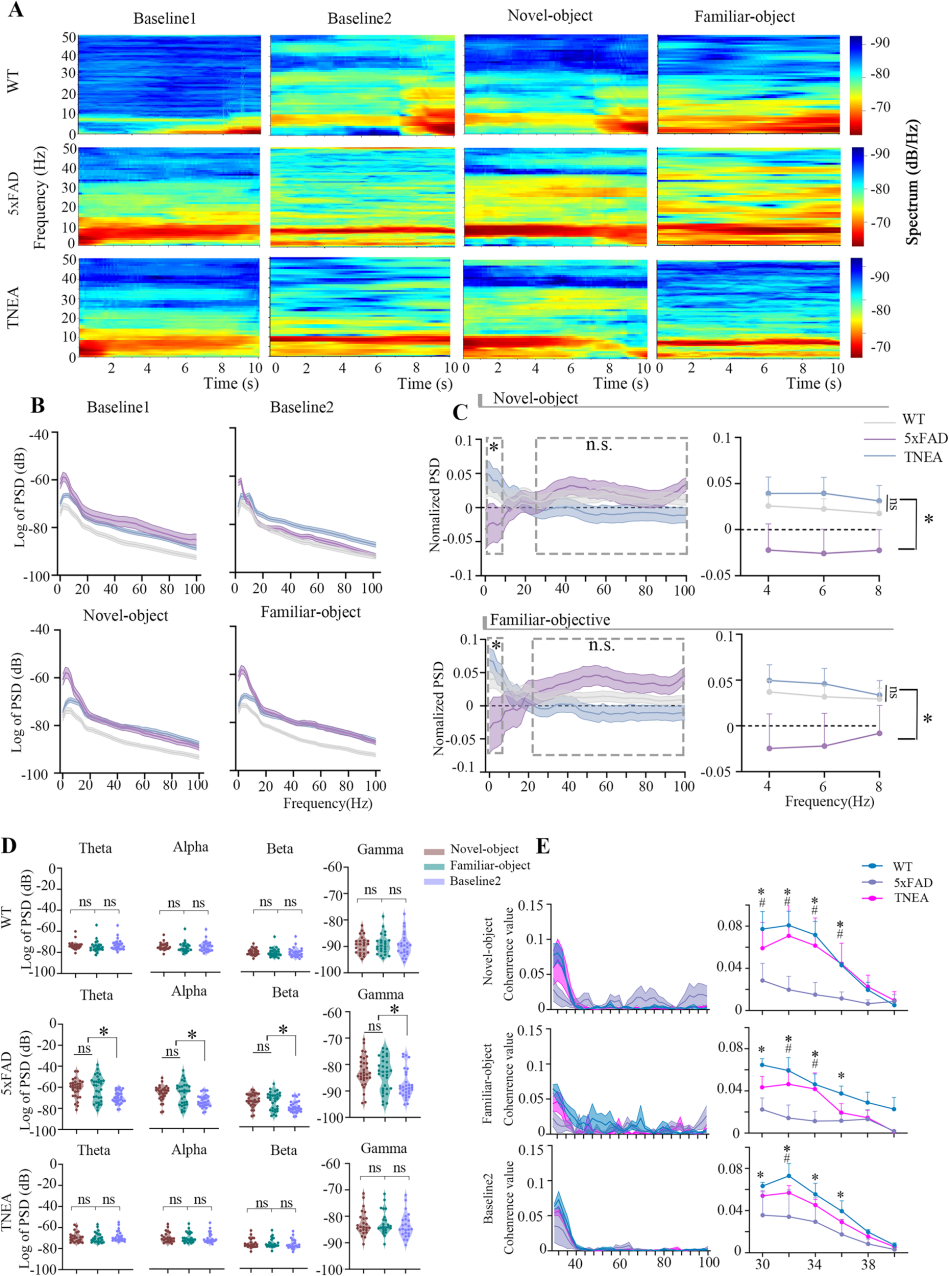
TNEA increased the theta‐band PSD and theta/gamma coherence in the hippocampal CA1 of 5xFAD mice during the NORT. A) Schematic diagram of time‐frequency analysis of LFP signals in WT (upper), 5xFAD (middle), and TNEA mice (lower). (Baseline 1: habituation; Baseline 2:the phase of exploring in the arena other than the novel or familiar object; Novel‐object: the phase of exploring novel object; Familiar‐object: the phase of exploring familiar object. B) PSD analysis of LFP in WT, 5xFAD, and TNEA mice during the phase of Baseline 1, Baseline 2, Novel‐ and Familiar‐object. C): Normalized PSD (Calculated by the mean deviation of the PSD of each mouse when it explores novel/familiar object relative to Baseline 1) for broadband LFP (0 – 100 Hz; upper left) and theta oscillation (upper right) in WT, 5xFAD, and TNEA‐treated mice during novel object exploration. Normalized PSD for broadband LFP (0 – 100 Hz; lower left) and theta oscillation (lower right) in WT, 5xFAD, and TNEA‐treated mice during familiar object exploration (n = 5 per group). D) PSD across frequency bands during novel‐object, familiar‐object, and baseline‐2 exploration (n = 5 per group). E) Changes in the coherence values between theta and gamma oscillations (n = 5 per group) Statistical significance was set at *p < 0.05, ns: not significant two‐way ANOVA with Tukey's multiple comparisons test. All data are expressed as mean ± s.e.m.

During the exploration of the novel and familiar objects, the theta band power of the 5xFAD mice was reduced compared to the WT group, and TNEA increased the theta band power in the 5xFAD mice (**Figure** **4**A). Using Baseline1 as a reference, we calculated the rate of power change of oscillations in the three groups. (Figure 4B). The theta‐band power of the 5xFAD mice decreased during the novel and familiar object exploration compared to the WT group, while TNEA increased theta power (Figure 4C). Additionally, we observed no changes in broad‐band oscillations in the WT group during the three exploration stages. In contrast, the power of broad‐band oscillations increased in the 5xFAD mice during the explorations, while TNEA could decrease the power of broad‐band oscillations (Figure 4D). Figure 4E illustrates the Pearson correlation between theta and gamma powers. The coherence between theta and gamma powers was reduced in the 5xFAD mice compared to the WT mice during the exploration stages, while TNEA increased the coherence of the two bands' oscillation powers. We further quantified key oscillatory properties, including peak frequency and spectral half‐width. During the NORT, TNEA treatment enhanced theta oscillations’ peak frequency (Figure S3C,D, Supporting Information) and significantly reduced the spectral half‐width of theta activity (Figure S3F, Supporting Information). In contrast, TNEA did not alter the peak frequency of gamma oscillations, either during quiescent states or during NORT (Figure S3A,B, Supporting Information). However, it did lead to a reduction in the spectral half‐width of gamma oscillations specifically during quiescent states (Figure S3E, Supporting Information).

Prior studies^[^ ^49^ ^]^ have established a close relationship between oscillatory activities in the CA1 and CA3 regions of the hippocampus. CA3 plays a critical role in the generation of oscillations.^[^ ^50^, ^51^, ^52^, ^53^ ^]^ In the present study, we recorded LFPs from both CA3 and CA1. Our results demonstrate that TNEA treatment enhanced oscillatory power in the CA3 region (Figure S4C,D, Supporting Information). Furthermore, it significantly increased coherence between CA1 and CA3, indicating strengthened functional connectivity in this hippocampal circuit (Figures S4,‐F and S5B,C, Supporting Information). TNEA improves theta oscillations during the NORT, and these improvements are likely derived from the CA3 region of the hippocampus.″

### 2.5 TNEA Rescued the Abnormal Firing of Pyramidal Neurons in 5xFAD Mice During NORT

A single neuron can fire locked to different LFP oscillations. So, we wondered whether the decrease in theta oscillation power in the 5xFAD group of mice was related to the firing of different kinds of neurons. We recorded all neurons’ firing rates in three groups of mice during NORT. The WT group of mice showed a significant decrease in firing rates during the exploration stages compared to the baseline, whereas there was no significant difference in the 5xFAD group. And the TNEA decrease the abnormal firing in 5xFAD mice (Figure S6A, Supporting Information). Firing rates change of 5xFAD mice was significantly higher in pyramidal neurons (Figure S6B, Supporting Information bottom) compared with mice in the WT group, while there was no significant change in interneurons (Figure S6C, Supporting Information (bottom)). We also found that TNEA can rescue the abnormal firing of pyramidal neurons in 5xFAD mice (Figure S6A,B, Supporting Information).

### 2.6 TNEA Restored Theta Oscillation Without Relying on the Abnormal Firing of Specific Pyramidal Neurons in 5xFAD Mice During NORT

**Figure 5:**
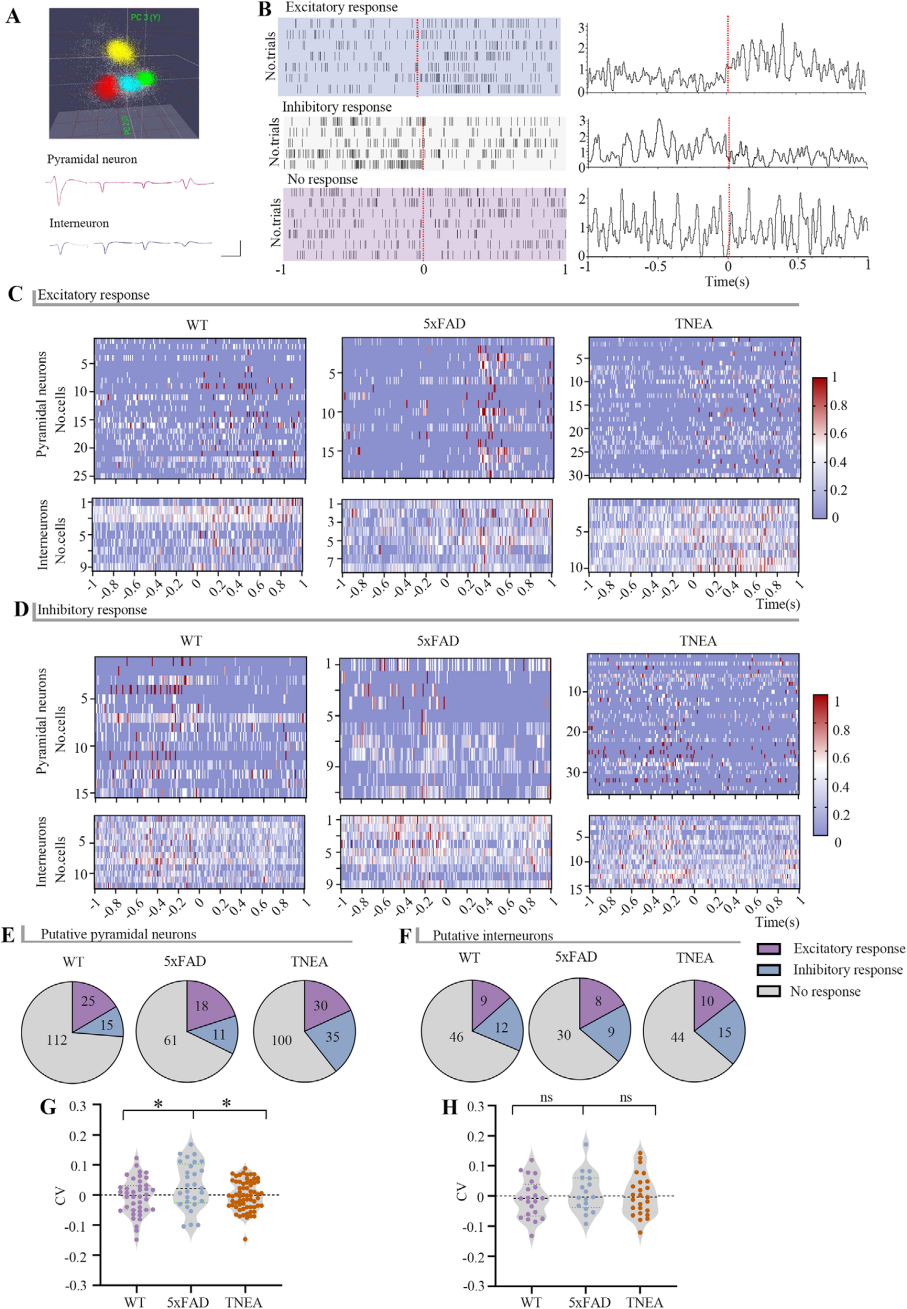
Changes in firing rates of putative interneurons and pyramidal neurons in WT, 5xFAD and TNEA‐treated mouse groups. A) Representative spike waveforms of a putative pyramidal neuron and a putative interneuron recorded by tetrode are displayed in red and blue, respectively. Scale bars 0.7ms 0.1 mv. B) Typical spike responses to novel‐object stimuli for excitatory (top), inhibitory responses (middle), and no response (bottom). C) Representative normalized firing rates (Firing rates were normalized per unit by dividing by maximum firing rate) of excitatory responses in single‐unit recordings during object exploration (aligned to exploration onset). Top: Putative pyramidal neurons; Bottom: Putative interneurons. Color scale indicates firing activity intensity (blue: low; red: high). Groups: WT, 5xFAD and TNEA‐treated. D) Representative normalized firing rates of inhibitory responses in single‐unit recordings during object exploration (aligned to exploration onset). Top: Putative pyramidal neurons; Bottom: Putative interneurons. Color scale indicates firing activity intensity (blue: low; red: high). Groups: WT, 5xFAD and TNEA‐treated. E,F) Neuron counts with differential responses to novel object exploration in WT, 5xFAD, and TNEA‐treated groups (E: putative pyramidal neurons, F: putative interneurons). Excitatory, no response, and inhibitory response are indicated in purple, blue and gray, respectively. G,H) CV in mean firing rates relative to exploration onset across WT, 5xFAD, and TNEA‐treated groups (G: putative pyramidal neurons, H: putative interneurons). Statistical significance was set at *p < 0.05, one‐way ANOVA with Tukey's multiple comparisons test, ns: not significant. All data are expressed as mean ± s.e.m.

The discriminating ability between novel and familiar objects is dominated by specific neurons. We recorded neurons that responded to novel and familiar objects (referred to as object‐reactive neurons). In total, we recorded 219 neurons (152 pyramidal neurons and 67 interneurons) in WT mice, 137 neurons (90 pyramidal neurons and 47 interneurons) in 5xFAD mice, and 234 neurons (165 pyramidal neurons and 69 interneurons) in the TNEA group of mice. Among these, the object‐reactive pyramidal neurons exhibited excitatory responses at rates of 16.4%, 20%, and 18.2%, respectively, as well as inhibitory responses at rates of 9.9%, 12.2%, and 21.2% (**Figure** **5**E). Interneurons displayed similar patterns, with excitatory responses at rates of 13.4%, 17%, and 14.5%, and inhibitory responses at 17.9%, 19.1%, and 21.7% (Figure 5F). We recorded neuronal changes from 1 s before to 1 s after the novel object was touched in all three groups of mice (Figure 5C,D). The excitatory responses of each pyramidal neuron across the three groups are shown in the top section of Figure 5C, while the excitatory responses of each interneuron are presented in the bottom section. Similar to the inhibitory responses are displayed in Figure 5D.

The coefficient of variation (CV) represent the change of firing rates before 500 ms and 500 ms after the novel object was touched. The CV of pyramidal neurons was elevated in 5xFAD mice compared to WT mice, while TNEA reduced the CV (Figure 5G). We also calculated the CV for interneurons and found no statistically significant differences among the three groups (Figure 5H).

Additionally, we calculated CV when familiar object was touched (Figure S7A,B, Supporting Information). No significant differences in the CV for either pyramidal neurons or interneurons among the three groups (Figure S7E,F, Supporting Information). Interestingly, the CV of pyramidal neurons in the 5xFAD group that exhibited inhibitory responses was elevated, but this did not affect the overall CV of the pyramidal neurons (Figure S8B, Supporting Information, bottom). It remains unclear whether the abnormalities in 5xFAD pyramidal neurons during novel object exploration contribute to the reduced power of theta oscillation in these mice and whether TNEA enhances theta oscillation in 5xFAD mice by improving the firing of pyramidal neurons.

The MVL of object‐reacted pyramidal neurons is significantly higher than the object‐reacted interneurons along the theta oscillations (Figure S9A, Supporting Information). This indicates that theta oscillations are more correlated with object‐reacted pyramidal neurons in all groups of mice. Thus, there is still no explanation for the impaired theta oscillations in 5xFAD mice.

### 2.7 TNEA Rescued the Impaired Inhibitory Monosynaptic Connections in 5xFAD During NORT

**Figure 6:**
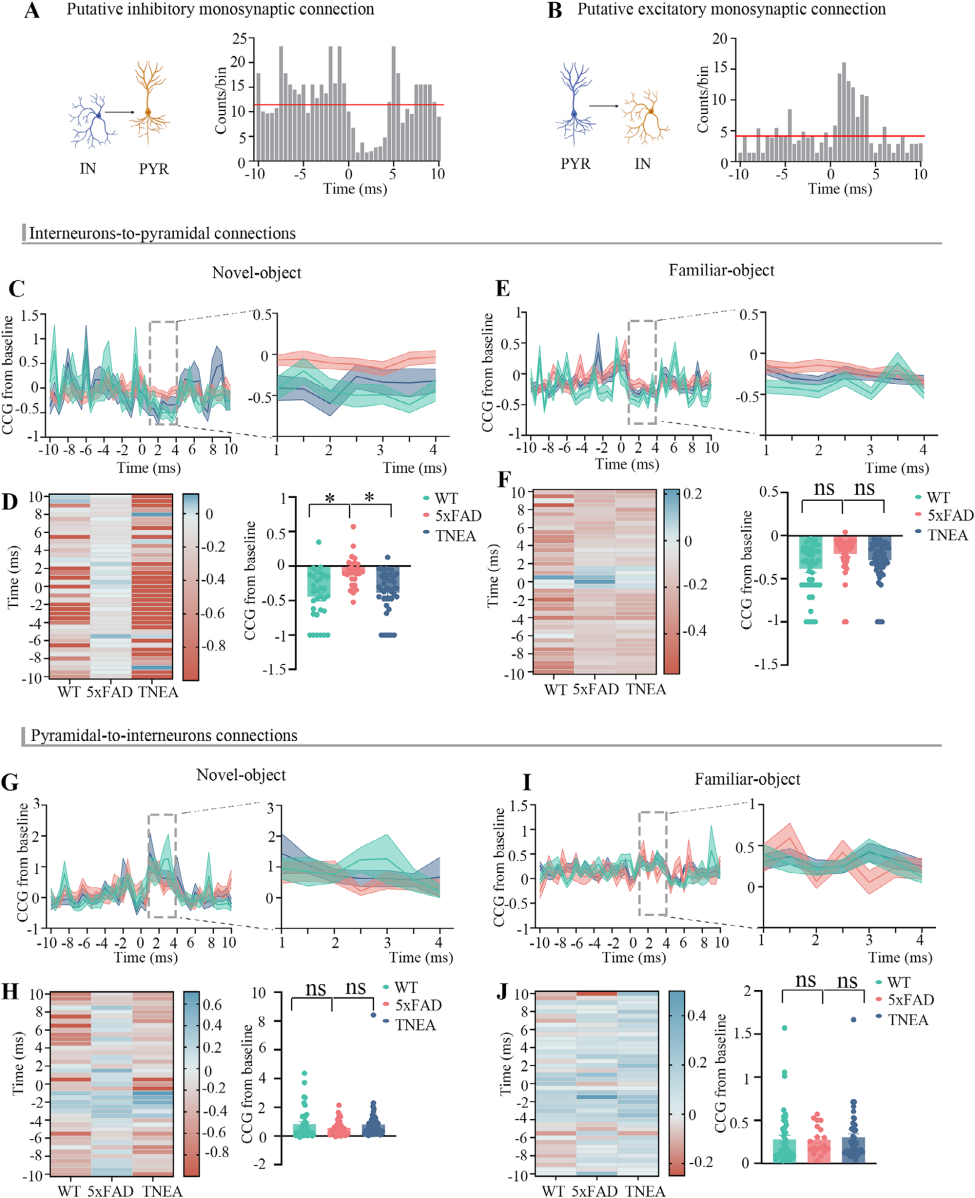
TNEA strengthened inhibitory monosynaptic (interneuron (INT) to pyramidal neuron (PYR)) connections in 5xFAD mice. A) Representative cross‐correlogram between INT to PYR showed a possible monosynaptic and inhibitory interaction. Reference events corresponded to the spikes of the presynaptic neurons (bins of 0.5 ms). Left: Illustration of connection type; Right: example of a INT to PYR connection with CCG values ranging from −10 to +10 ms time lag. Dashed line indicates baseline (the baseline is defined as the average CCG of the lags from 5 to 6 ms or 1 ms before the monosynaptic connection latency windows). B) Like (A), it is the schematic diagram of excitatory connections targeting PYR to INT connections. C) Left: Mean CCGs of INT to PYR cell pairs during the exploration of novel‐object in mice of the WT, 5xFAD, and TNEA groups, with a time lag ranging from −10 to +10 ms, Normalized CCG (Calculated by the geometric mean firing rate and shown as the difference from baseline) for connection strength. Right: zoomed‐in view of the left connection strength plot at 1 to 4 ms time lags. D) Connection strength of mice in the WT, 5xFAD, and TNEA groups. Left: Connection strength of every INT to PYR cell connection pairs; right: Averaged connection strength. Each dot represents the connection strength measured for a single cell pair. (n = 32 pairs for WT, n = 28 pairs for 5xFAD, n = 41 pairs for TNEA). E) like (C), it is targeting INT to PYR cell pairs during familiar‐object exploration. F) like (D), it is targeting INT to PYR cell pairs during familiar‐object exploration. Right: averaged connection strength. Each dot represents the connection strength. (n = 44 pairs for WT, n = 42 pairs for 5xFAD, n = 51 pairs for TNEA). G) Left: Mean CCGs of PYR to INT cell pairs during the exploration of novel‐object in mice of the WT, 5xFAD, and TNEA groups, with a time lag ranging from −10 to +10 ms. Normalized CCG for connection strength. Right: zoomed‐in view of the left connection strength plot at 1 to 4 ms time lags. H) Connection strength of mice in the WT, 5xFAD, and TNEA groups. Left: connection strength of every cell connection pairs, right: averaged connection strength of every cell connection pairs. Each dot represents the connection strength. (n = 42 pairs for WT, n = 33 pairs for 5xFAD, n = 51 pairs for TNEA) I) Like (G), it illustrated the connections from PYR to INT during familiar‐object exploration. J) Like (H), it showed the connections from PYR to INT during familiar‐object exploration. (n = 44 pairs for WT, n = 42 pairs for 5xFAD, n = 51 pairs for TNEA). All data are expressed as mean ± s.e.m. Statistical significance was set at *p < 0.05, one‐way ANOVA with Tukey's multiple comparisons test, ns: not significant.

Many studies^[^ ^54^, ^55^, ^56^ ^]^ have reported that inhibitory monosynaptic connections in the hippocampal CA1 region of 5xFAD model mice are weaker compared to those in WT mice. We first aimed to determine whether synaptic efficacy in vivo in 5xFAD mice was similarly altered during novel and familiar object test. To assess synaptic connectivity in awake mice in a behavioral state, we employed a well‐established method of measuring synaptic connectivity and connection strength through extracellular recordings.^[^ ^57^, ^58^, ^59^ ^]^ In this method, putative monosynaptically connected cell pairs are identified by calculating the crosscorrelogram (CCG), which reveals pairs of cells that exhibit a significant increase or decrease at time delays (i.e., 1 to 4 ms), thus defining monosynaptic connectivity (see methods for details). Putative excitatory and inhibitory connections were identified by detecting significant peaks or valleys in the CCG at 1 to 4 ms delay compared to the baseline (the baseline is defined as the average CCG of the lags from 5 to 6 ms or 1 ms before the monosynaptic connection latency windows) (**Figure** **6**A,B).^[^ ^60^ ^]^ We measured connection strength during periods of exploring novel and familiar objects. The strength of inhibitory connections between interneurons and pyramidal neurons was lower in 5xFAD mice than in WT mice during novelty exploration, and TNEA significantly improved this reduction in the strength of inhibitory monosynaptic connections in 5xFAD mice (Figure 6C,D). During periods of familiar exploration, a trend toward reduced strength of inhibitory connections was observed in 5xFAD mice compared to WT mice, and TNEA also alleviated this trend (Figure 6E,F).

In contrast to INT to PYR connections, the strength of PYR to INT connections did not significantly differ among the three groups of mice (Figure 6G–J). Inhibitory synaptic transmission is a fundamental mechanism that regulates the dynamics of neural circuits, ensuring proper network function and oscillatory activity. Disruption of inhibition, as seen in AD, can lead to a cascade of effects, including network hyperexcitability, disrupted oscillations, and cognitive impairments. Understanding the complex relationship between inhibitory transmission and neuronal dynamics is crucial for developing therapeutic strategies for diseases.

### 2.8 TNEA Treatment May Enhance Theta Oscillation in 5xFAD Mice by Strengthening the Inhibitory Monosynaptic Connections During NORT

**Figure 7:**
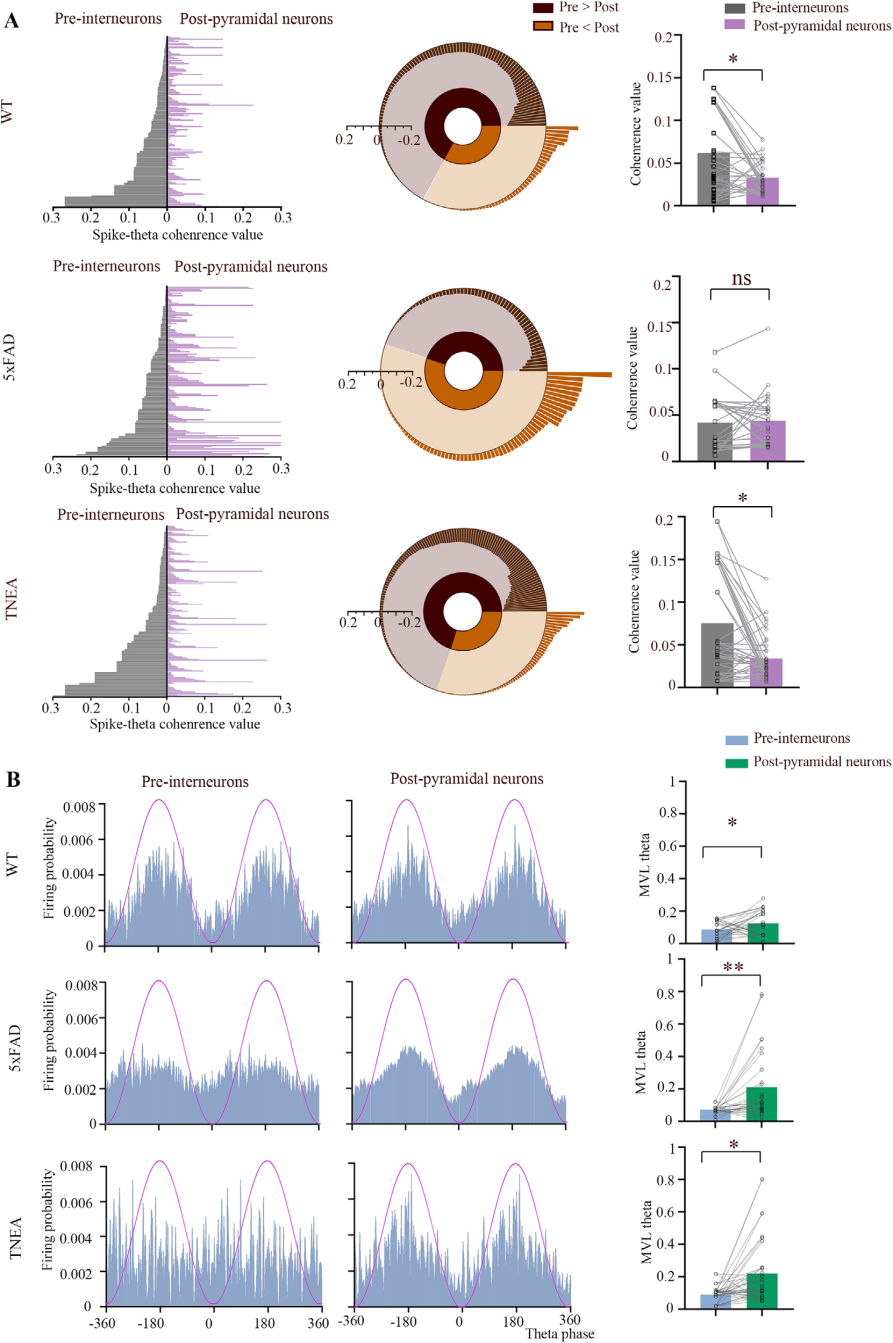
TNEA treatment enhanced theta oscillation in 5xFAD mice by strengthening INT to PYR connections during NORT. A) Correlation between the rate histogram of every IN‐to‐PYR cell pair with theta oscillation in the mice of WT, 5xFAD and TNEA groups (left). Correlation difference of every INT‐to‐PYR cell pair. Black: postsynaptic pyramidal neurons with theta oscillation have a greater coherence value than presynaptic interneurons with theta oscillation; yellow: postsynaptic pyramidal neurons with theta oscillation have a greater coherence value than the presynaptic interneurons with theta oscillation (middle). Mean coherence value of each group of cell pairs with theta oscillation (Right). B) Representative plots of firing of INT to PYR connections cell pairs distribute along different phases of theta oscillation in mice of WT, 5xFAD, and TNEA groups.(left and middle). MVL values of the putative presynaptic interneurons compared to the putative pyramidal neurons in each group during novel‐object exploration (Right). (n = 32 pairs for WT, n = 28 pairs for 5xFAD, n = 41 pairs for TNEA). Statistical significance was set at *p < 0.05, paired t test ns: not significant. All data are expressed as mean ± s.e.m.

To explore this relationship, we calculated the coherence between the spike firing of neurons and the power of oscillation.^[^ ^61^ ^]^ We assessed the coherence value of presynaptic interneurons with theta oscillation and postsynaptic pyramidal neurons with theta oscillation. We found that presynaptic interneurons exhibited a greater coherence value than postsynaptic pyramidal neurons in WT mice. In contrast, in 5xFAD mice, there was no change in the coherence value of presynaptic interneurons and postsynaptic pyramidal neurons with theta oscillations. However, TNEA could improve this abnormal state of coherence in the 5xFAD group (**Figure** **7**A).

We then questioned whether TNEA only affects firing rates. We calculated the firing phase over theta oscillation and assessed MVL for spike and theta oscillations. The presynaptic interneurons had a lower coherence value than postsynaptic pyramidal neurons across all three groups of mice (Figure 7B). This suggests that TNEA changes only the rate histogram of presynaptic interneurons and does not affect the firing phase over theta oscillations.

Additionally, The coherence value between the rate histogram of each presynaptic pyramidal neuron‐to‐postsynaptic interneuron cell pair with theta oscillation has no significant difference in WT and 5xFAD mice. In TNEA mice, postsynaptic interneurons with theta oscillation had a greater coherence value than presynaptic pyramidal neurons with theta oscillation (Figure S10A, Supporting Information). TNEA may rescue theta oscillations by enhancing inhibitory synaptic activity. However, we still needed to demonstrate whether TNEA could improve cognition simply by altering theta oscillations, so we applied a chemogenetic approach to inhibit interneurons.

### 2.9 Chemogenetic Inhibition of PV^+^ Interneurons in the Hippocampal CA1 Reversed the Effects of TNEA on the Cognitive Function of 5xFAD Mice

**Figure 8:**
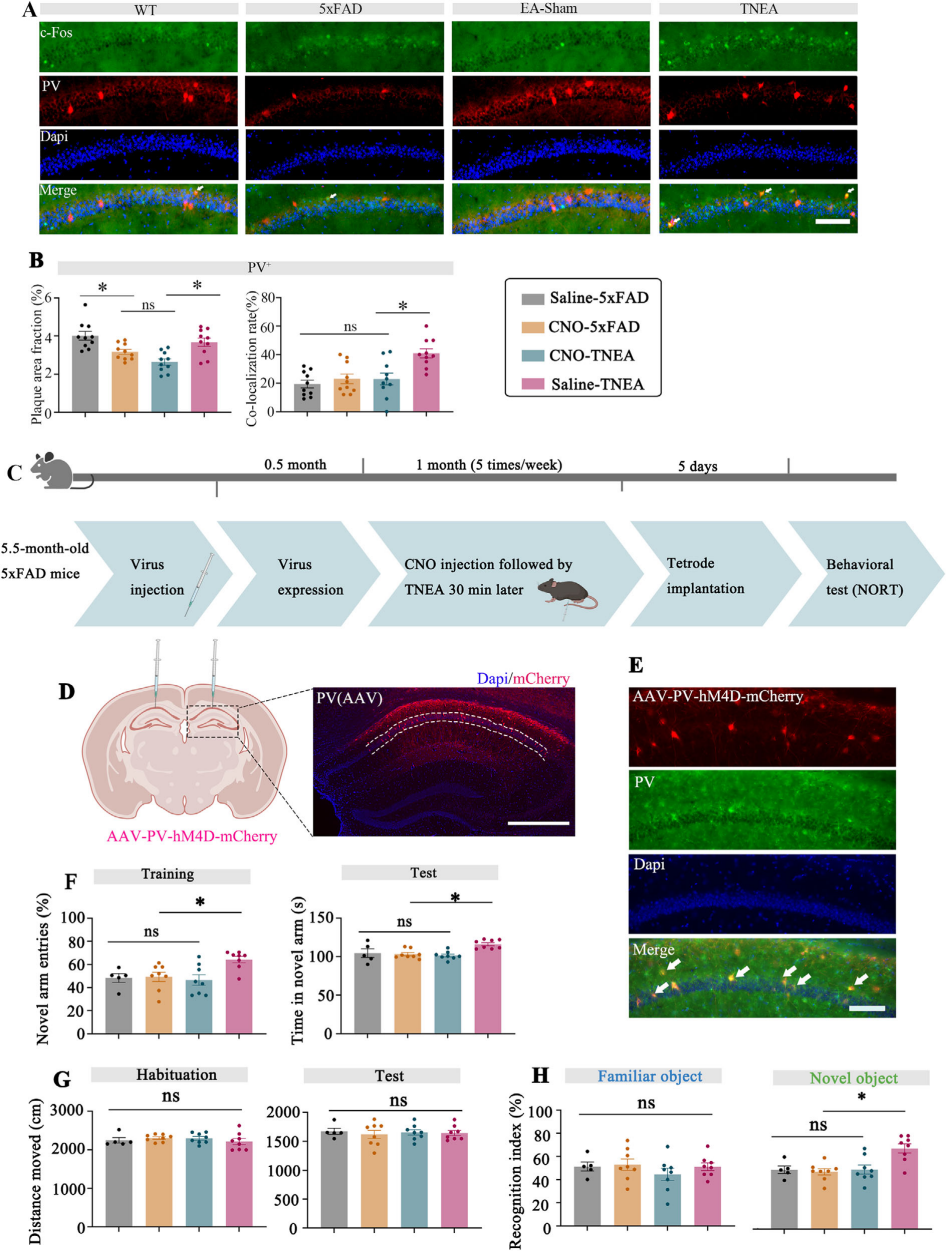
TNEA improved the cognitive function in 5xFAD mice via PV^+^ cells. A) Shown are representative images for immunohistochemical staining of c‐Fos (first row; green) staining in PV^+^ neurons (second row; red), Dapi (the third row, blue) and their colocalization (last row) in the CA1 region of 4 groups. B) Quantification of PV^+^ fluorescence intensity (left) and the percent colocalization of the c‐Fos with PV^+^ in the CA1. (n = 5 per group) C) Experimental procedures. D) Expression of hM4D‐mCherry in the CA1 of a mouse. Scale bar represents 500 µm. E) Shown are representative images for expression of AAV‐PV‐hM4D‐mCherry (first row; red) immunohistochemical staining in PV^+^ neurons (second row; green), Dapi (the third row, blue) and their colocalization (last row) in the CA1 region of 4 groups. Scale bar represents 100 µm F) The percentage of new arm entries in the Y maze (left) and the time spent in the new arm of the Y maze (right). (n = 5 for Saline 5xFAD n=8 for CNO‐5xFAD, TNEA, and Saline‐TNEA per group). G) Distance traveled during the habituation and test sessions during NORT. (n = 5 for Saline 5xFAD n=8 for CNO‐5xFAD, TNEA, and Saline‐TNEA per group) H) The recognition index of 4 groups was detected by NORT (n = 5 for Saline 5xFAD n=8 for CNO‐5xFAD, TNEA, and Saline‐TNEA per group). Statistical significance was set at *p < 0.05, one‐way ANOVA with Tukey's multiple comparisons test, ns: not significant. All data are expressed as mean ± s.e.m.

We wondered whether the activation of interneurons is required for TNEA treatment to be effective. To identify the neuronal subtypes modulated by TNEA, we employed c‐Fos immunofluorescence combined with cell‐type‐specific markers. Our results indicated that TNEA predominantly activated PV^+^ interneurons, as evidenced by a significant increase in c‐Fos expression colocalized with PV^+^ (**Figure** **8**A,B). In contrast, there was no difference observed in c‐Fos expression colocalized with pyramidal neurons (CaMKII^+^) (Figure S11A,B, Supporting Information), suggesting a selective regulatory effect of TNEA on PV^+^ neurons over pyramidal neurons. We used chemogenetic techniques to selectively modulate the activity of PV^+^ interneurons. AAV‐PV‐hM4D‐mCherry was injected into the CA1 region of the hippocampus to selectively express PV^+^ interneuron (Figure 8D,E). The behavioral and intracerebral changes associated with TNEA were observed after intraperitoneal injections of Clozapine‐N‐oxide (CNO). As shown in NORT, TNEA treatment failed to increase the RI in the 5xFAD mice with specific inhibition of the PV^+^ interneuron. In contrast, the RI was improved in the saline group during the test (Figure 8E). A similar finding to the NORT results was found in the Y‐maze, where TNEA treatment increased both entries and time spent in new arms in the saline group with TNEA treatment, but not in the CNO group (Figure 8C,D). Notably, specific inhibition of the PV^+^ interneuron prevented the improvement of cognitive function in 5xFAD mice by TNEA, indicating that the activation of PV^+^ interneurons was crucial for TNEA treatment.

Chemogenetic inhibition of PV^+^ interneurons in the hippocampal CA1 did not influence the effects of TNEA on Aβ accumulation and microglial phenotypic transition of 5xFAD mice. According to the results of Thio‐S staining in the hippocampal CA1, TNEA treatment (Saline or CNO injection) would reduce the Aβ plaque deposits significantly in 5xFAD mice (Figure S12A,D, Supporting Information). In the IF of Iba‐1 and CD68/Arg‐1, we were able to confirm that the CNO was not preventing microglial morphology changes and phenotype transitions. As the results illustrated, in the CNO‐EA and Saline‐TNEA 5xFAD mice group, we both noticed a decrease in CD68 staining (Figure S12B,E, Supporting Information), an increase in Arg‐1 staining (Figure S12C,F, Supporting Information), as well as an increase in microglial total branch lengths (Figure S12G, Supporting Information) and endpoints (Figure S12H, Supporting Information). These results confirmed that the effects of TNEA treatment on Aβ accumulation and microglial phenotypic switching in 5xFAD mice were unrelated to the activation of PV^+^ interneurons in the hippocampal CA1.

### 2.10 Chemogenetic Inhibition of PV^+^ Interneurons Reversed the Effects of TNEA on Gamma Oscillations of 5xFAD Mice

**Figure 9:**
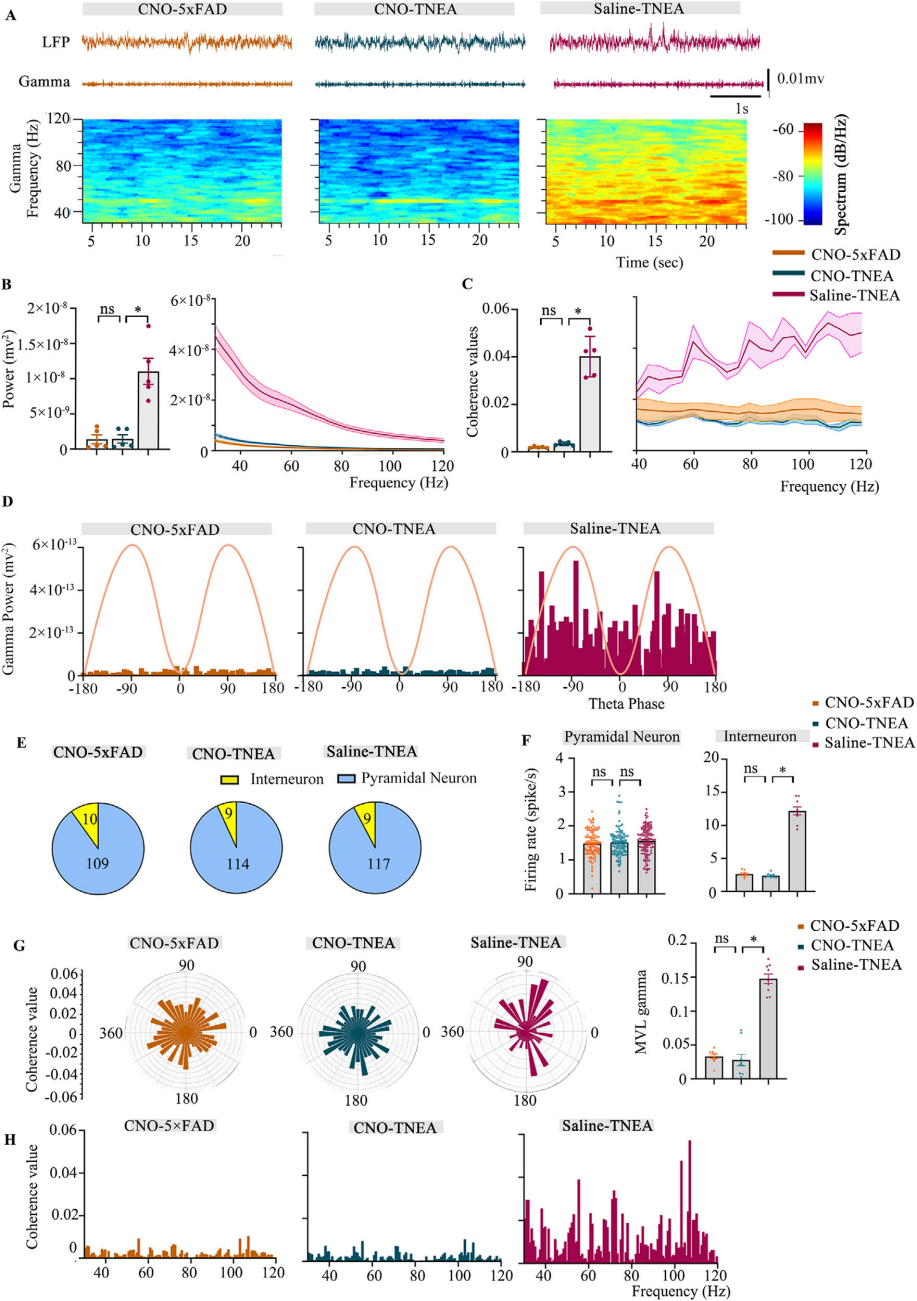
TNEA enhanced the gamma oscillations in 5xFAD mice by activating interneurons in the hippocampal CA1. A) Representative 1D view of LFP filtered gamma oscillations (top panel) and power spectrogram of gamma oscillations (lower panel) from 3 groups. B) PSD of gamma oscillations from 3 groups. (n = 5 mice per group). C) Coherence values of high gamma and theta oscillation of 3 groups. (n = 5 mice per group). D) Phase‐amplitude coupling of gamma power over theta phases of 3 groups. E) The number of putative pyramidal neurons and putative interneurons recorded in hippocampal CA1 of from 3 groups. F) Left: The firing rate of putative pyramidal neurons (left) and putative interneurons (right) from 3 groups. (putative pyramidal neurons: n = 109 for CNO‐5xFAD, n = 114 for CNO‐TNEA, n = 117 for Saline‐TNEA, putative interneurons: n = 10 for CNO‐5xFAD, n = 9 for CNO‐TNEA, n = 9 for Saline‐TNEA). G) Left: representative polar plots of putative interneurons’ spikes distributions along the gamma phase from 4 groups. Right: MVL of 3 groups. (n = 10 for CNO‐5xFAD, n = 9 for CNO‐TNEA, n = 9 for Saline‐TNEA). H) Coherence values of putative interneurons’ spikes and gamma oscillations of 3 groups. Statistical significance was set at *p < 0.05, one‐way ANOVA with Tukey's multiple comparisons test, ns: not significant. All data are expressed as mean ± s.e.m.

The LFP in vivo recording in the hippocampal CA1 showed that the reduction in gamma oscillations was the same irrespective of whether EA was conducted or not after the inhibition of PV^+^ interneurons in 5xFAD mice. However, the decline in gamma oscillations was relieved in the 5xFAD group that received saline‐TNEA (**Figure** **9**A,B). According to the present results, CA1 PV^+^ interneurons dysfunction was sufficient to disrupt the effect of TNEA on the coherence value in terms of high gamma and theta oscillations, with the increased coherence values shown only in the Saline‐TNEA group (Figure 9C). A further finding was that the phase‐amplitude coupling between theta and gamma in both CNO groups (with or without EA) decreased significantly compared with Saline‐TNEA group (Figure 9D). It was obvious that TNEA could only increase the phase‐amplitude coupling between theta and gamma in the Saline‐TNEA group, while no change was observed in the CNO‐TNEA group. Therefore, we proposed that PV^+^ interneurons might play a crucial role in TNEA‐induced gamma oscillations in the hippocampal CA1 of 5xFAD mice.

### 2.11 Chemogenetic Inhibition of PV^+^ Interneurons Impaired the Effects of TNEA on Increasing Putative Interneurons’ Firing Rates and Firing Phase Over Gamma Oscillation

There was also no difference among the three groups in terms of the percentage of interneurons since 10 of the total recorded neurons (10/109) in the CNO group, 9 of the total recorded neurons (9/114) in the CNO‐TNEA group, and 10 of the total recorded neurons (10/112) in the Saline‐TNEA group were sorted as interneurons (Figure 9E). Moreover, compared with Saline‐TNEA group, a significant decrease was observed in the firing rates of interneurons in both CNO and CNO‐TNEA group. We found TNEA could increase the firing rates of interneurons in the saline 5xFAD group, while the interneurons inhibited by CNO were unable to be activated by TNEA in the hippocampal CA1 (Figure 9F). Next, we observed that the lower coherence values between spikes of interneurons and gamma oscillations in 5xFAD mice treated with CNO could not be restored by TNEA, nevertheless, in the saline group, the increase could be clearly observed after TNEA treatment (Figure 9H). In addition, the impaired MVL, which measured the phase‐locking of interneuron spike firing and gamma oscillations, was elevated in the saline group. Still, not in the CNO group even received TNEA treatment (Figure 9G). In contrast, MVL of pyramidal neurons’ spike firing with gamma oscillations was no such phenomenon described above was observed (Figure S13, Supporting Information). TNEA may exert its effects through the activation of PV^+^ interneurons.

### 2.12 Chemogenetic Inhibition of PV^+^ Interneurons in the Hippocampal CA1 Reversed the Effects of TNEA in Improving the Theta Oscillation Power in 5xFAD Mice

**Figure 10:**
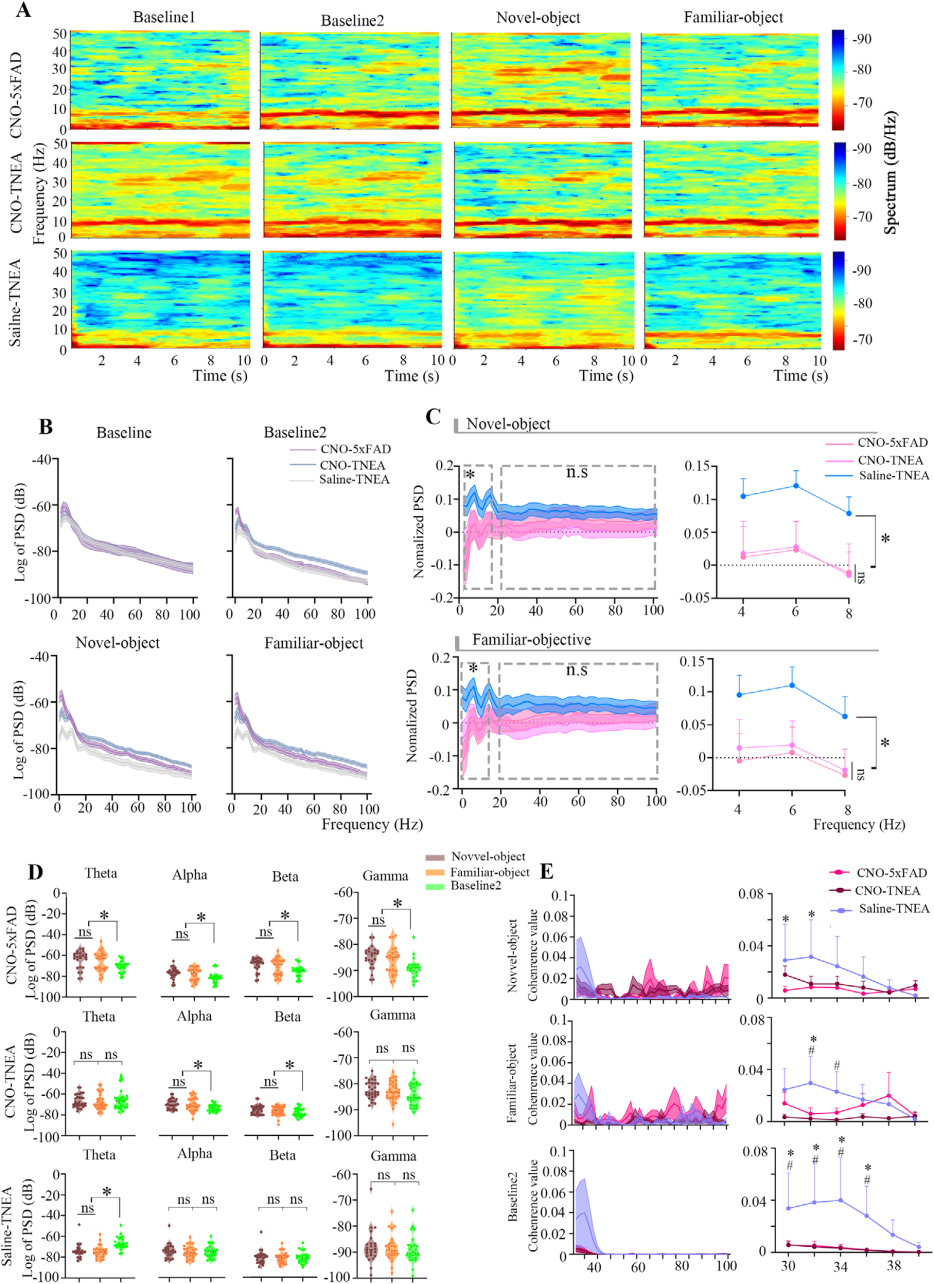
TNEA increased the power spectral of theta oscillation in the hippocampal CA1 of 5xFAD mice during the NORT by activating PV^+^ neurons. A) Schematic diagram of time‐frequency analysis of LFP signals in CNO‐5xFAD (upper), CNO‐TNEA (middle), and Saline‐TNEA mice (lower). (B) PSD analysis of LFP in the habituation stage (baseline1), in the arena instead of the novel or familiar object (baseline2), in the arena of novel object, and familiar object. C) Normalized PSD (Calculated by the mean deviation of the PSD of each mouse when it explored novel/familiar object relative to Baseline 1) (n = 5 mice per group) (upper left). Normalized theta oscillation PSD during novel objects exploration. (n = 5 mice per group) (upper right). Normalized LFP PSD during familiar objects exploration. (n = 5 mice per group) (lower left). Normalized theta oscillation PSD during familiar objects exploration. (n = 5 mice per group) (lower right). D) PSD of each group during exploring novel object, familiar object, and other arenas. (n = 5 mice per group). E) The coherence values between theta and gamma oscillations. Statistical significance was set at *p < 0.05, two‐way ANOVA with Tukey's multiple comparisons test, ns: not significant. All data are expressed as mean ± s.e.m.

We recorded the LFP signals of three groups of mice (CNO‐5xFAD, CNO‐TNEA, Saline‐TNEA) during four exploration stages, with a cumulative total of 10 s for each stage. The inhibition of PV^+^ neurons for TNEA during the exploration of the novel and familiar objects no longer increased the power of theta oscillations in the 5xFAD mice (**Figure** **10**A). Additionally, we performed a power spectral analysis of the LFP signals from the three groups of mice across four time stages (Figure 10B). Using Baseline1 as a reference, inhibiting PV^+^ neurons for TNEA treatment did not enhance the theta band power in the 5xFAD mice during the exploration of novel and familiar objects (Figure 10C).

Moreover, we found that after inhibiting PV^+^ neurons and administering TNEA, there was no decrease in the power of any frequency band of oscillation in the 5xFAD mice during the exploration of novel and familiar objects (Figure 10D). Figure 10E illustrates the Pearson correlation between theta and gamma powers, demonstrating that the coherence between theta and gamma powers is reduced in the CNO‐TNEA and Saline‐TNEA groups compared to the CNO‐5xFAD group during the exploration stages of novel and familiar objects. TNEA may rescue theta oscillations by activating PV^+^ interneurons.

### 2.13 TNEA May Prevent the PV^+^ Interneurons’ Loss and Upregulate BDNF Via the cAMP/PKA/CREB Pathway

TNEA prevents the degeneration of PV^+^ interneurons, preserving their population during the progression of AD in the 5xFAD mice (Figure S14A,B, Supporting Information). However, TNEA was not able to increase the number of PV^+^ cells when they were inhibited with CNO (Figure S14A,B, Supporting Information). Furthermore, 5xFAD mice showed low levels of cAMP (Figure S14C, Supporting Information), p‐PKA, p‐CREB, and BDNF (Figure S14D, Supporting Information). However, it should be noted that the CNO application blocked activation, whereas the opposite occurred for the CNO group with no change in cAMP, p‐PKA, p‐CREB, and BDNF levels. Furthermore, we performed fluorescence colocalization analysis to assess the relationship between BDNF expression and PV^+^ neurons. While an overall increase in BDNF levels was observed in TNEA group, the degree of colocalization between BDNF and PV^+^ neurons remained consistent across all four experimental groups, indicating no significant alteration (Figure S15A, Supporting Information). These results suggested that TNEA may not activate BDNF via the cAMP/PKA/CREB pathway in the hippocampus of 5xFAD mice by activating PV^+^ interneurons. The underlying mechanisms responsible for this phenomenon remain to be elucidated.

## 3 Discussion

Our results indicate that theta and gamma oscillation were reduced in 5xFAD mice during quiescence or NORT. There is a deficit in inhibitory synaptic strength in AD mice during NORT, suggesting a close link between synaptic dysfunction and interneuron deficits. The activity of PV^+^ interneurons is necessary for the therapeutic effects of TNEA. TNEA is leading to an increase in gamma oscillations in 5xFAD mice during the quiescent state, as well as enhancing the power of theta oscillations, possibly by strengthening the presynaptic PV^+^ interneurons during NORT. Consequently, cognitive function in the mice improved across all measures.

There is strong evidence that neuroinflammation is a critical factor in the development of AD.^[^ ^62^, ^63^ ^]^ Microglia play a significant role in triggering inflammatory reactions within the central nervous system,^[^ ^64^ ^]^ while the deposit of Aβ in the AD brain directly damages neural cells and activates microglia.^[^ ^65^ ^]^ In previous studies, other investigators have provided substantial evidence for the therapeutic effects of acupuncture in neurodegenerative diseases.^[^ ^43^, ^66^, ^67^ ^]^ One of these studies has shown that TNEA attenuates Aβ pathology and enhance cognitive function in 5xFAD mice.^[^ ^43^ ^]^ Moreover, TNEA inhibited Aβ‐induced activation of NLRP3 inflammatory vesicles in the hippocampus of 5xFAD mice.^[^ ^68^ ^]^ However, we incorporated chemogenetic inhibition of PV^+^ neurons and observed that, Contrary to the expected changes in Aβ deposition and microglial activation, there was also a significant improvement in neuroinflammation. Also, Our behavioral studies demonstrated that TNEA improved cognitive function in AD mice, This suggests that the resolution of neuroinflammation and the restoration of network dynamics by TNEA may occur through alternate pathways, further supporting the complexity of neuroinflammation regulation in AD. But, our study demonstrated that TNEA significantly alleviated Aβ deposition and prevented abnormal activation of microglia in the hippocampus of 5xFAD mice; thus, TNEA has potent anti‐inflammatory effects. However, the neurobiological mechanisms by which TNEA stimulation modulates cognitive function and underline neural activity in the brain remain unclear.

**Figure 11:**
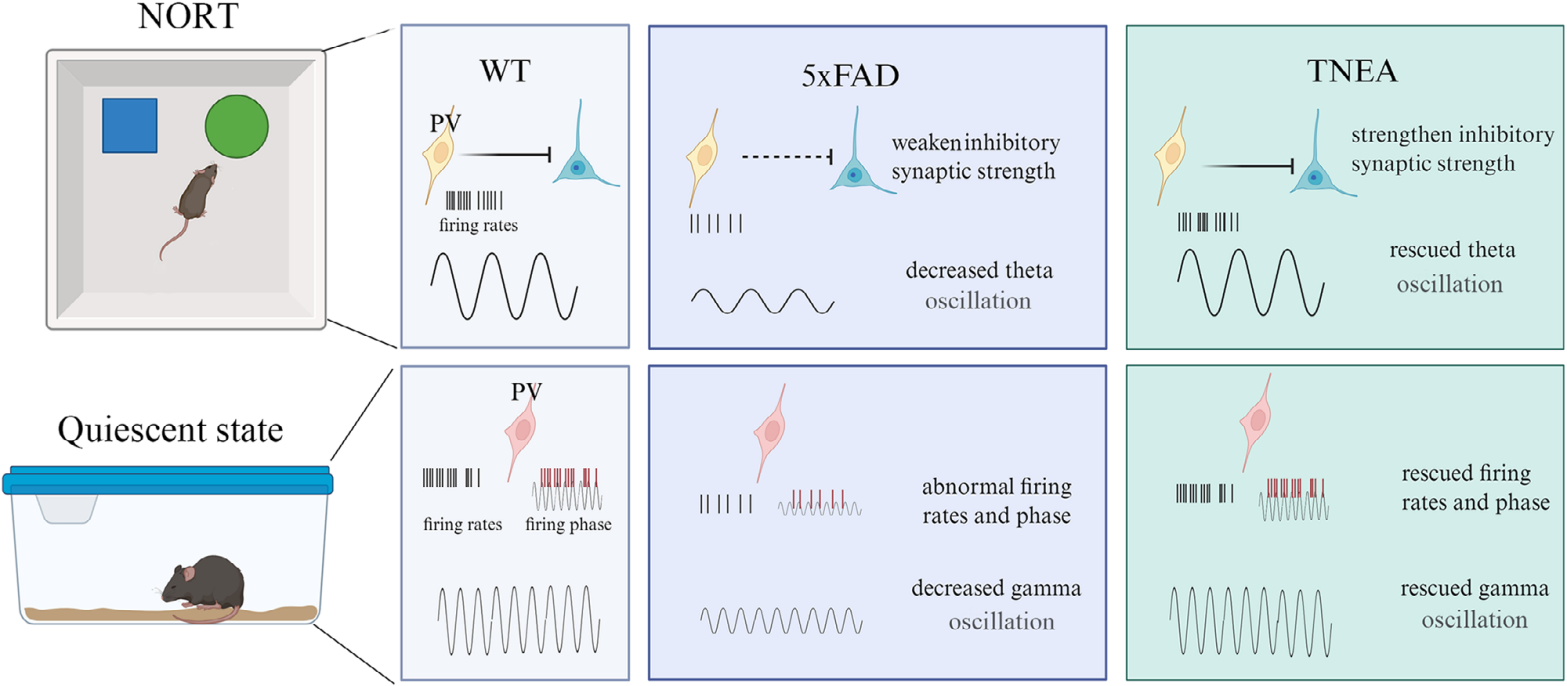
Mechanism of TNEA regulated AD. A schematic model proposing how TNEA rescued cognitive function in 5xFAD mice by activating PV^+^ neuron in quiescent state and strengthening presynaptic inhibitory interneurons involved in monosynaptic connections.

Our previous research, along with that of others showed an overall reduction in theta oscillation during quiescent or NORT in 5xFAD mice.^[^ ^69^, ^70^ ^]^ Additionally, in state of anesthesia, 6‐month‐old 5xFAD mice exhibited differential effects on LFP gamma oscillation, showing reduced power in the CA1 region.^[^ ^71^ ^]^ Our findings align with those of previous studies, indicating that both theta and gamma oscillations are reduced in AD mice during the quiescent states, and the theta power in 5xFAD mice decreases when they explore novel/familiar objects. In contrast, TNEA treatment appears to increase gamma oscillation power during quiescent and enhance theta oscillation during the NORT. However, the cause of these abnormalities in theta and gamma oscillations is much debated, leaving the mechanism for TNEA‐induced enhancement of oscillations in both frequency bands and the resulting cognitive improvement unresolved. (**Figure** **11**).

Synaptic dysfunction in AD has garnered significant interest due to the strong correlation between synaptic loss and cognitive impairment in AD.^[^ ^26^ ^]^ Previous studies indicate that spatial memory impairments in 5xFAD mice are associated with molecular markers of synaptic degradation, as evidenced by decreased levels of presynaptic markers (such as syntaxin and synaptic vesicle proteins) and postsynaptic markers (like PSD‐95).^[^ ^72^, ^73^, ^74^ ^]^ Moreover, synaptic dysfunction is critical in various neurodevelopmental and neurodegenerative disorders, and inhibitory synapses are essential for maintaining network oscillations, including theta synchronization.^[^ ^75^, ^76^, ^77^ ^]^ Previous studies examining pyramidal cell spine density in 5xFAD mice have found significant spine loss in somatosensory cortex, prefrontal cortex, and the hippocampus.^[^ ^22^, ^78^, ^79^ ^]^ While there have been a large number of previous studies on the role of Aβ on synaptic activity in vitro^[^ ^80^, ^81^ ^]^ or in vivo in anesthetized animals,^[^ ^82^, ^83^ ^]^ To investigate these synaptic changes, current studies utilize advanced techniques in awake, behaving animal models.

Our study recorded electrical activity from several individual neurons in the CA1 area of the hippocampus and showed that the 5xFAD mice had weak inhibitory synaptic connections. We discovered that the presynaptic interneurons involved in inhibitory synaptic connections were more linked to theta power in WT mice, but the situation was different for the synaptic connections in 5xFAD mice. TNEA rescued this loss of synaptic efficacy in 5xFAD mice. Interestingly, however, TNEA rescued theta oscillations only by increasing firing rates without altering the firing phase in theta oscillation in 5xFAD mice (Figure 11).

Neuronal activity is closely associated with oscillations, with the specific phase of oscillation being influenced by the type of activated cell.^[^ ^84^ ^]^ In the context of AD, inhibitory interneuron dysfunction has been widely reported in mouse models. Among these, PV^+^ neurons—the largest subclass of GABAergic neurons, which collectively account for ≈20% of neurons play a particularly critical role.^[^ ^85^ ^]^ PV^+^ neurons are highly interconnected via gap junctions, enabling synchronized firing that is modulated by pyramidal neurons.^[^ ^86^ ^]^ This synchronized firing significantly enhances the precision and consistency of action potentials in pyramidal neurons, which in turn results in oscillatory activity.^[^ ^75^ ^]^ Several studies have demonstrated a loss of PV^+^ inhibitory interneurons in 5xFAD mice.^[^ ^87^, ^88^ ^]^ In ApoE4 mice, which carry a major genetic risk factor for late‐onset sporadic AD, the loss of these interneurons have been associated with deficits in learning and memory.^[^ ^23^ ^]^ These findings collectively indicate widespread impairment of inhibitory function across multiple brain regions and AD mouse models. Previous research in mice has indicated that gamma synchronous modulation among PV^+^ interneurons within the parahippocampal prefrontal cortex is crucial for behavioral adaptation.^[^ ^89^ ^]^ Additionally, in nonhuman primates, changes in theta oscillation dynamics in the parahippocampal cortex are broadly correlated with gamma oscillation during tests involving working memory.^[^ ^90^ ^]^

We selectively inhibited PV^+^ neurons in CA1 through intraperitoneal injection of CNO followed by TNEA. We found that TNEA no longer improved the power of gamma oscillations during quiescence, suggesting that one of the mechanisms by which TNEA enhances gamma oscillations is through the activation of PV^+^ interneurons. Similarly, when we inhibited PV^+^ neurons in CA1, TNEA no longer increased the power of theta oscillations during the exploration of novel and familiar objects in 5xFAD mice (Figure 11). The generation of oscillations is influenced by a multitude of factors, including neuronal firing rates, spike timing,^[^ ^91^ ^]^ and the specific architecture of the local microcircuitry^[^ ^49^ ^]^ in which PV^+^ interneurons are embedded. In the present study, the enhancement of oscillations induced by TNEA likely involves specific components of this complex system. Future studies need to fully elucidate the precise contributions of these diverse elements to theta oscillation generation, The precise mechanism by which PV^+^ neurons mediate the effects of TNEA remains a central question for future investigation. particularly employing techniques such as electrophysiological recordings in brain slices to address the central question of the precise mechanism by which PV^+^ neurons mediate the effects of TNEA.

Of course, no mouse model reproduces AD completely.^[^ ^92^ ^]^ For example, the 5xFAD mouse models used in this study do not mimic all of the symptoms of human AD, most notably their lack of neurogenic fiber tangles, using p‐tau181 as a biomarker, which is a key feature of the disease.^[^ ^29^ ^]^ In addition, the 5xFAD mouse model is a relatively aggressive model with rapid disease progression.^[^ ^93^ ^]^ A shared susceptibility of inhibitory interneurons is evident across multiple mouse models of AD, despite their differing genetic pathogenesis.

It is important to note that measuring synaptic connectivity in vivo is a complex test, spike sorting alone cannot definitively distinguish all interneuron subtypes without molecular confirmation, our approach is consistent with established in vivo electrophysiology practices.^[^ ^94^ ^]^ And the main limitation of our findings is that interconnection measurements can only indirectly detect monosynaptic connectivity and connection strength. It cannot accurately detect connections in cells with low firing rates, and it can only quantify the transmission of action potentials, not postsynaptic potentials. In addition, the total number of connections detected was limited. We cannot detect all synaptic connections; even considering these limitations, this method of detecting single synaptic connections and measuring synaptic strength is well established, has been used in many other studies, and has been validated by optogenetics and paracellular stimulation.^[^ ^58^, ^95^, ^96^ ^]^

Overall, TNEA appears to be a promising treatment for AD that can simultaneously target the improvement of cognitive function in mice in a less invasive manner. Further studies are needed to fully understand its mechanisms and broader clinical applications in AD. Future work utilizing ex vivo approaches will be essential to determine if TNEA directly enhances the excitability and activity of PV^+^ interneurons or acts through an alternative intermediary mechanism that ultimately contingent upon PV^+^ cell function.

## 4 Experimental Section

### Animal Grouping

The 5xFAD mouse model is an APP/PS1 double transgenic line that expresses the 695‐amino‐acid isoform of human amyloid precursor protein (APP695) carrying the Swedish (K670N/M671L), Florida (I716V), and London (V717I) familial AD mutations, together with human presenilin 1 (PSEN1) containing the M146L and L286V mutations. Heterozygous mice used in this study were generated by crossing homozygous 5 x FAD mice with C57BL/6J mice. All animals were obtained from the Model Animal Research Center of Nanjing University (Nanjing, China) —Nanjing Institute of Biomedicine (No. 201 400 975) and maintained at 22 ± 2 °C and 60 ± 10% relative humidity with free access to standard food and water. Mice were genotyped as previously described.^[^ ^97^ ^]^ All mice were aged 5 months before the start of experiments. Mixed male and female 5XFAD mice, along with age and sex matched WT littermates, were utilized for electrophysiology experiments, behavioral experiments, and immunohistochemical analyses. The animal care and experimental protocols were approved by the Animals Research Ethics Committee of the Shanghai University of Traditional Chinese Medicine (No. PZSHUTCM200821019). Experimenters conducting behavioral tests, histological analyses, and molecular assays were blinded to mouse genotypes.

### Electroacupuncture Treatment

EA was conducted according to previous research.^[^ ^43^ ^]^ The mice gas anesthetized with 1% isoflurane (Sigma, St. Louis, MO) were placed on a temperature‐controlled heating pad (Sider Technology, China) for maintaining the animal's core temperature. It is recommended that the skin directly surrounding each acupoint should be cleaned with alcohol swabs prior to the insertion of stainless‐steel needles (0.16 mm in tip diameter, 15 mm in length, Huatuo Brand, Suzhou, China) into the GV24 and bilateral GB13 acupoints at a depth of 10 mm. GV24 is situated 1.3 mm directly above the midpoint between the mouse's eyes, while GB13 is located 2 mm bilaterally from GV24. Both acupoints are found on the scalp, near the frontal pole. Electrical stimulation pulses (0.3 mA, 2 Hz) with 500 µm continuous wave were applied for 15 min per day (5 days per week, 4 weeks) by an electrical stimulator (Wuxi Jiajian Medical Instrument Co., Ltd., China). For the Sham‐EA condition, we inserted the needles into the skin at the surface of the acupoints (GV24 and bilateral GB13) without performing any electrical stimulation. For the Non‐TN‐EA condition, the needles were inserted into a location 1 mm away from the acupoint, specifically at a non‐acupoint site, while still maintaining proximity to the targeted acupoint (GV24 and bilateral GB13) with electrical stimulation.

### The Novel Object Recognition Test

NORT was conducted as previously described,^[^ ^98^ ^]^ the cognitive performance was assessed by allowing mice to freely explore novel versus familiar objects in a standardized arena. Briefly describe as follows, it was conducted in 3 stages: habituation, familiarization and testing. For the habituation phase, mice were allowed to explore the open‐field (30 cm × 30 cm) for 10min. For the familiarization phase, mice were permitted to explore two objects in an open‐field area for 5 min. During the testing phase, which was 1 h after the training, the mice were put back to explore in this arena for another 5 min, where one of the objects was replaced by a novel object of different shape and color. The animals were compared based on the recognition index (RI) (%), which is computed as the ratio of time spent investigating novel object over the total time spent in exploring both of them. As part of the investigation of an object, the mice sniffed, touched, or climbed onto it, all of which were analyzed and recorded by the EthoVision video tracking system (Noldus Information Technology, Netherlands).

### Y‐Maze Behavioral Tests

We assessed the animals’ cognitive ability by employing the Y‐maze behavioral paradigm.^[^ ^99^ ^]^ In brief, the mice were allowed to run freely in the two open arms of the maze for 5 min with an arm closed. After 30 min, the mice were placed again in this Y‐maze with all arms open for 5 min. A video tracking system, EthoVision (Noldus Information Technology, Netherlands), was used to analyze and record the time spent in the novel arm and the times of all the arm entries.

### Thioflavin S Staining

Thio‐S staining was performed according to our previously published protocols.^[^ ^100^ ^]^ The brain tissue was sectioned at 15 µm and mounted on glass coverslips. Air‐dried brain sections were stained for 8 min in the dark with 1% thioflavin S (1326‐12‐1, Sigma, St. Louis, MO) in 50% ethanol (Sigma, USA) and then dehydrated for 5 min in three ethyl alcohol solutions (80%, 90%, and 100% alcohol), followed by fluorescent mounting medium (345 789, Sigma, St. Louis, MO). The observation and photography were conducted using a fluorescence microscope (Axio Observer 5, Zeiss, Oberkochen, Germany). 3 brain slices from each mouse (5 mice in each group) were analyzed. As mentioned before,^[^ ^53^ ^]^ the amount of Aβ plaques was counted in the hippocampal CA1 for quantification. The percentage of positive signals in the selected area, relative to the total area, was calculated using ImageJ software.

### Immunofluorescence

In PBS, the brain sections were blocked in 0.3% TritonX‐100 and 5% bovine serum albumin (BSA) for 1 h at room temperature (RT). Then the sections were incubated with the primary antibodies: rabbit anti c‐fos (1:400; Santa, USA), in combination with either mouse anti‐PV (PV3144, 1:200; Invitrogen, USA), or mouse anti‐CaMKIIα(ab22609, abcam, USA). anti‐BDNF(ab108319, abcam, USA), Rabbit anti‐Iba‐1 (019‐19741, 1:500; Wako, Japan) mixed with mouse anti‐CD68 (YM3050, 1:200, Immunoway, USA) or rat anti‐Arg‐1 (SG‐271430, 1:200; Santa, USA). NeuN (1:200, MAB377, Sigma, USA), in blocking buffer overnight at 4 °C. Afterward, the sections were cultured in the dark with the secondary antibodies: goat anti‐rabbit Alexa Fluor 488 (1:1000, A11034, Thermo Fisher Scientific, USA) mixed with goat anti‐mouse Alexa Fluor 488 (1:1000, No. A21151, Thermo Fisher Scientific, USA), goat anti‐rabbit Alexa Fluor 555 (1:1000, A21428, Thermo Fisher Scientific, USA), goat anti‐rabbit Alexa Fluor 555 (1:1000, A21428, Thermo Fisher Scientific, USA), mixed with goat anti‐mouse Alexa Fluor 488 (1:1000, No. A21151, Thermo Fisher Scientific, USA) or goat anti‐rat Alexa Fluor 488 (1:1000, No. A11006, Life Technologies, USA) for 2h at RT. Following each of the above steps, the sections were washed three times with PBS. The nuclei were counterstained by DAPI (No. C1006, Beyotime, China) and finally mounted with fluorescent mounting medium (No. P0126, Beyotime, Shanghai, China). Confocal images were captured using a laser‐scanning confocal microscope (Leica TCS SP2, Germany). According to previous research,^[^ ^44^ ^]^ the number of branching endpoints and the total length of branches of each microglia were calculated. For fluorescence staining analysis: 2 brain slices from each mouse (5 mice in each group) were analyzed in PV Co‐localization experiment. 3 brain slices from each mouse (5 mice in each group) were analyzed in Iba 1 Co‐localization experiment. The percentage of positive signals in the selected area, relative to the total area, was calculated using ImageJ software.

### Cyclic Adenosine Monophosphate ELISA

A direct competitive ELISA (ab65355, Abcam, USA) was used to measure cAMP levels in the hippocampus. Based on the manufacturer's instructions, we first weighed the brain tissue and added five times the volume of 0.1M HCl. Afterward, the tissue was homogenized and centrifuged for 5 min. After loading the supernatant into 96‐well plates, the cAMP antibody was incubated for 1 h. Subsequently, we added cAMP‐HRP to each well and incubated it for another hour. The samples were washed three times with washing buffer, followed by an incubation of 1 h with the HRP developer. After stopping the reaction with 1 M HCl, the color developed was measured using an ELx800 plate reader (BioTek, USA).

### Western Blot

The radioimmunoprecipitation (RIPA) assay buffer containing protease and phosphatase inhibitor mixture was used to extract the protein from the hippocampus. The homogenization, sonication, and centrifugation of the hippocampus cells at 14,000g and 4 °C for 15 min should then be performed. Then the BCA Protein Assay kit (Thermo Fisher Scientific, USA) was used to determine the protein concentration. Afterward, proteins were boiled for 10 min in glycerol‐based Eagle's medium containing SDS at 100 °C. As soon as the proteins were separated by 12.5% SDS‐PAGE (Yeasen Biotech, Shanghai, China), the proteins were transferred to nitrocellulose membranes (Yeasen Biotech, China). We blocked the membranes for 1 h with 5% BSA in a Tris‐Buffered Saline Tween‐20 (TBST) buffer (10 mmol of Tris, pH 7.5; 100 mmol of NaCl; 0.1% Tween 20). Afterward, the membranes were incubated overnight at 4 °C with specific primary antibodies (1:1000 dilution in 5% nonfat milk in the TBST buffer) of rabbit anti‐PKA (No. ab108385, Abcam, USA), rabbit anti‐p‐PKA (Cat. No. ab75991, Abcam, USA), rabbit anti‐CREB (3955, CST, USA), rabbit anti p‐CREB (ab32096, Abcam, USA), rabbit anti‐BDNF (ab223354, CST, USA), mouse anti‐β‐Tubulin (No. 10 094, Proteintech, USA). On the second day, the primary antibody was detected with anti‐rabbit (or anti‐mouse) secondary antibodies (1:1000; No. 32 460, Life Technologies, Carlsbad, CA;), and the immunoreactive bands were visualized with an enhanced chemiluminescence kit (MA0186, Meilunbio, Dalian, China). In the end, the band density was quantified with Image J software.

### Tetrode Electrode Implantation Surgery and Data Collecting

The animals were gas anesthesia by isoflurane (1%) and then positioned on a stereotactic frame (RWD, Shenzhen, China) for tetrode implantation surgery. During electrophysiological studies, a small hole was drilled and the dura mater was removed above the place of interest. Self‐made movable bundles of the 32‐channel tetrode electrode^[^ ^97^, ^101^ ^]^ consisting of 8 independently movable tetrodes, targeting the mice's hippocampal CA1 (AP:−2 mm, ML:±1.8 mm, and DV:−1.1 mm), cortex (AP:−2 mm, ML:±1mm, and DV:−0.5mm), and DG (AP:−2 mm, ML:±1.6mm, and DV:−2.5mm) Four stainless steel screws were used as ground screws above the cerebellum and parietal cortex. Following the implantation of tetrodes at a depth of 0.7 mm, the electrodes were secured on the scull using dental cement. Electrodes were made of wires (California Fine Wire Company, CA) and electrodes tips to an impedance of 0.5 – 1 MΩ (Measured by Nano Z) on the day of implantation. Movable microdrivers were advanced ≈70 µm, 1.1 mm depth was reached during habituation to the test equipment 4 to 7 days before the first recording session was conducted. For multi‐electrode recordings of CA3 and CA1 activity, Self‐made movable bundles of the 64‐channel tetrode electrode^[^ ^89^, ^90^ ^]^ consisting of 8 independently movable tetrodes, targeting the mice's hippocampal CA1 (AP:−2 mm, ML:±1.6 mm, and DV:−1.1 mm) and CA3 (AP:−2 mm, ML:±2.35 mm, and DV:−2.35 mm)

After surgery, mice were allowed for recovery at least 1 week before recording. Neural signals were collected by the Plexon OmniPlex Neural Data Acquisition System (Plexon, USA) and analyzed by NeuroExplorer Version 5 (Nex Technologies, USA). The local field potentials (LFP) value refers to the real‐time potential under the sampling frequency of 1000 Hz.

After recording, all animals were deeply anesthetized, and the recording sites were outlined using a direct current (20 mA for 10 s) through the electrode. Then their brains were sectioned to verify electrode positions histologically according to the lesion created by the current.

### Stereotaxic Injection

Animals using stereotaxic intracranial injections, recombinant adeno‐associated viruses (rAAV) were delivered in vivo target in CA1 (AP:−2 mm, ML:±1.8 mm, DV: −1.1mm)/CA3 (AP:−2 mm, ML: ±2.35 mm, DV:−2.35 mm), including AAV2/9‐PV‐hM4D‐mCherry‐WPREpA (titer: 1.6 × 10^12^), AAV2/9‐PV‐hM3D‐GFP‐WPRE‐pA (titer: 2.45 × 10^12^) (purchased from Taitool Shanghai, China), The rAAV was injected into the target zone of the gas anesthetized (isoflurane (1%)) mice using a 10 µL Hamilton microsyringe (Hamilton Co.) with a micro‐injection needles (diameter of 0.06 mm) purchased from RWD technology company, on a stereotactic frame at a constant speed of 30 nL min^−1^ (PUMP 11 ELITE Nanomite; Harvard Apparatus). 2 weeks later, clozapine (CNO) (sigma, USA) was administrated intraperitoneally for chemogenetic suppression.

### Statistical Analysis

Behavioral data acquisition

A camera (30 – 80 fps) was placed above the animals to record the timing of animals’ sat quietly (quiescence state) in home cage or during the NORT. The recording software synchronize the video timestamp and neural activity automatically. By using the above methods, accurate timestamps of animals’ touching the object were captured on video. The spike and LFP were recorded during the home cage and NORT.

For analysis, recording epochs were calculated based upon a time period of mice’ quiescence or touching the novel/familiar object. Sessions in which the data were collected from damaged electrodes were discarded.

### LFP Analysis

This work conducted the analysis of time‐frequency data (TFR) by using a Fourier transform with multipliers. A time‐frequency spectrum was computed for a latency range of 0 to 25 s and a frequency range of 1 to 100 Hz during quiescence. In the NORT, this work focused on the time‐frequency spectrum within a 0 to 10 s latency window.

In the analysis of specific frequency bands, this work applied a FIR Hann filter for frequency band extraction.

This work also analyzed the power of LFP at various frequency bands, including theta (4–8 Hz), alpha (8–12 Hz), beta (15–29 Hz), low gamma (30–60 Hz), and high gamma (60–100 Hz). For each selected continuous variable, this work calculated either a standard or multi‐taper power spectrum, utilizing the Welch periodogram method in both cases.

Furthermore, this work employed NeuroExplorer to measure coherence, which indicates the degree of relationship between two time series as a function of frequency. In this analysis, coherence was calculated for theta and gamma bands during both quiescence and the NORT. The fast Fourier transforms (FFTs) of the theta and gamma bands' variable values were computed following specified preprocessing and the application of the designated tapering window. Individual and cross‐densities were subsequently calculated: Pxx = FFT(X) * Conj(FFT(X)), Pyy = FFT(Y) * Conj(FFT(Y)), and Pxy = FFT(X) * Conj(FFT(Y)). Here, Conj(z) represents the complex conjugate of z. The Pxx, Pyy, and Pxy values were averaged across all intervals, and coherence values were derived from the formula Mean(Pxy) * Mean(Pxy) / (Mean(Pxx) * Mean(Pyy))^[^ ^61^ ^]^

This work analyzed the peak frequency as previously described^[^ ^53^ ^]^ using NeuroExplorer. First, the power spectra of the continuous data were selected. Then, the theta and gamma bands were filtered using an FIR Hann window filter. Finally, summary data were exported and the peak frequency value was identified. This work analyzed the spectral half‐width as previously described^[^ ^52^ ^]^ using NeuroExplorer based on the continuous power spectra. The procedure was performed as follows: 1. The theta and gamma bands were filtered using an FIR Hann window followed by Gaussian smoothing. 2. The peak amplitude (Pmax) was identified. The half‐amplitude threshold was defined as Pmax/2. 3. The two frequency points at which the amplitude equaled to Pmax/2 were located. 4. The half‐width was calculated as the difference between these two frequency points.

### Spike Analysis

Spike sorting involved separating neurons into putative excitatory and inhibitory cells based on the firing properties (rise, decay, and half‐width of spikes) as well as the autocorrelograms. Briefly, spikes were sorted by using offline Sorter and custom‐made Matlab and followed by manual adjustment using the software MClust 4.4 program (A. D. Redish, http://redishlab.neuroscience.umn.edu/ MClust/MClust.html).

This work analyzed firing rates during epochs of mouse quiescence or during the NORT. Using a paired‐sample t‐test, this work compared the firing rates of neurons within 500 ms after touching the novel or familiar object to the firing rates prior to the touching onset, aiming to identify neurons whose firing rates were modulated by the touching of novel or familiar objects. The results revealed three types of responses: excitatory responses (significantly higher firing rates post‐touching than pre‐touching), no responses (no difference between pre‐touching and post‐touching), and inhibitory responses (lower firing rates post‐touching than pre‐touching). An analysis of the proportion of neurons exhibiting different types of responses was presented in Figure 5.

A normalized firing rate was calculated by dividing the firing frequency of each neuron at each time bin by its maximum firing rate (Figures 5 and 6). The coefficient of variation (CV) represents the variability in the spiking patterns of individual neurons.^[^ ^102^ ^]^ We computed the CV as the mean firing rate pre‐touching minus the mean firing rate post‐touching.

### Monosynaptic Connections‘ Strength Analysis

Using NeuroExplorer, the crosscorrelogram displays the conditional probability of a spike at time t0+t, given a reference event at time t0. The crosscorrelogram also allows for the use of shift predictors. This work calculated the crosscorrelogram during novel and familiar object explorations, normalizing it by the geometric mean firing rate and presenting it as the difference from the baseline (connection strength). Connection strength^[^ ^54^ ^]^ was measured as the minimum value within the 1‐ to 4‐ms window, while the baseline corresponds to the lags from 5 to 6 ms or 1 ms before the monosynaptic connection latency windows.

### Coherence Analysis

This work assessed the spike‐field coherence (SFC) between spiking activity and gamma‐band oscillations. Coherence was defined only for continuously recorded signals; therefore, spike series must be converted into continuously recorded signals for coherence calculation.

To calculate the neuron's spike phase preference in NORT, this work utilized the LFP from the channel exhibiting the highest theta/gamma amplitude for all subsequent calculations. First, this work filtered segments of the LFP signal using the Find Oscillations option to extract theta/gamma band oscillations during periods of quiescence or NORT. Next, we added a new variable to the file: LFP_theta/gamma_ZeroPhase, which is an event variable containing the timestamps of the oscillation periods.

In the second step, this work employed the firing phase option using the following algorithm: this work selected the timestamps of the zero‐phase events for each interval of the oscillation period variable. The spike pulses occurring in each neuron during these theta/gamma periods were considered, and the spike pulse phase was calculated as 360*(spike_timezero_phase[i]) / (zero_phase[i+1]‐zero_phase[i]). To determine whether spike firing is uniformly distributed along the theta/gamma oscillation, this work generated a polar plot to display the probability distribution of the corresponding theta/gamma phase of the spikes. Phase locking was quantified using the mean vector length (MVL) of the phase distribution, ranging from 0 to 1. A MVL value of 1 indicates precise phase synchronization, while a value of 0 indicates no phase synchronization.

The Mean Vector Length was calculated as:

(1) $$\bar{R}=\left|\frac{1}{n}\sum_{s=1}^{n}Z_{S}\right|=\left|\frac{1}{n}\sum_{s=1}^{n}\exp\left(i\varphi_{s}\right)\right|$$

(2) $$Z=\cos\left(\theta \right)+\mathrm{isin}\left(\theta \right)=\exp\left(i\theta \right)$$

This work confirmed that Rayleigh's test for circular uniformity was indeed rigorously applied to assess the statistical significance of phase‐locking for each individually sorted unit,^[^ ^51^ ^]^ This work used the following formula to perform the Rayleigh test for assessing the significance of spike‐phase locking:

(3) $$Z=n{\bar{R}}^{2}$$

(4) $$P\approx e^{-Z}\left(1+\frac{2Z-Z^{2}}{4n}\right)$$

### Statistical Analysis

A mean and standard error of the mean (s.e.m.) are expressed for all data. All statistical analyses were performed using the Prism (version 9.03) software package. Normality was tested using the Shapiro‐Wilk test. For normal distribution data, equal variance was assessed using Bartlett's test. For not fully satisfy the normality assumption, equal variance was assessed using Brown‐Forsythe test. The data were compared using unpaired Student t‐tests in two experimental groups. multiple groups were analyzed by one‐way ANOVA. Two‐Way ANOVA with repeated measures for both factors between factors in two experimental conditions was conducted.

## Conflict of Interest

The authors declare no conflict of interest.

## Author Contributions

Z.G., H.N., Y.L., and Z.C., contributed equally to this work. D.W. and S.C. designed the study; Z.G. and H.N. wrote the manuscript and performed the study; Y.L., Z.C., Z.Z., and Y.W. analyzed the data; X.W., C.X., M.X., L.D., Y.Y., S.S., K.W., and Z.W. performed comments on the whole process of this study; and all authors have reviewed and approved the final version of the manuscript.

## Supporting information



Supporting Information

Supplemental Figure 1

Supplemental Figure 2

Supplemental Figure 3

Supplemental Figure 4

Supplemental Figure 5

Supplemental Figure 6

Supplemental Figure 7

Supplemental Figure 8

Supplemental Figure 9

Supplemental Figure 10

Supplemental Figure 11

Supplemental Figure 12

Supplemental Figure 13

Supplemental Figure 14

Supplemental Figure 15

Supplemental Figure 16

## Data Availability

The data that support the findings of this study are available from the corresponding author upon reasonable request.
