# Correction to “TNEA Regulates Hippocampal Oscillation by Improving Inhibitory Synaptic Plasticity to Ameliorates Cognitive Impairment in Alzheimer's disease”

**DOI:** 10.1002/advs.202521837

**Published:** 2025-11-20

In the original submitted manuscript, we found that the **examples of the heatmap** in **Figure 4A** were improperly used during sub‐figure assembly. The corrected figure is presented below. Upon meticulous review of the original records, we are confident that the correction does not impact the overall findings and conclusions of this paper.

https://doi.org/10.1002/advs.202510885

Corrected Figure 4A:


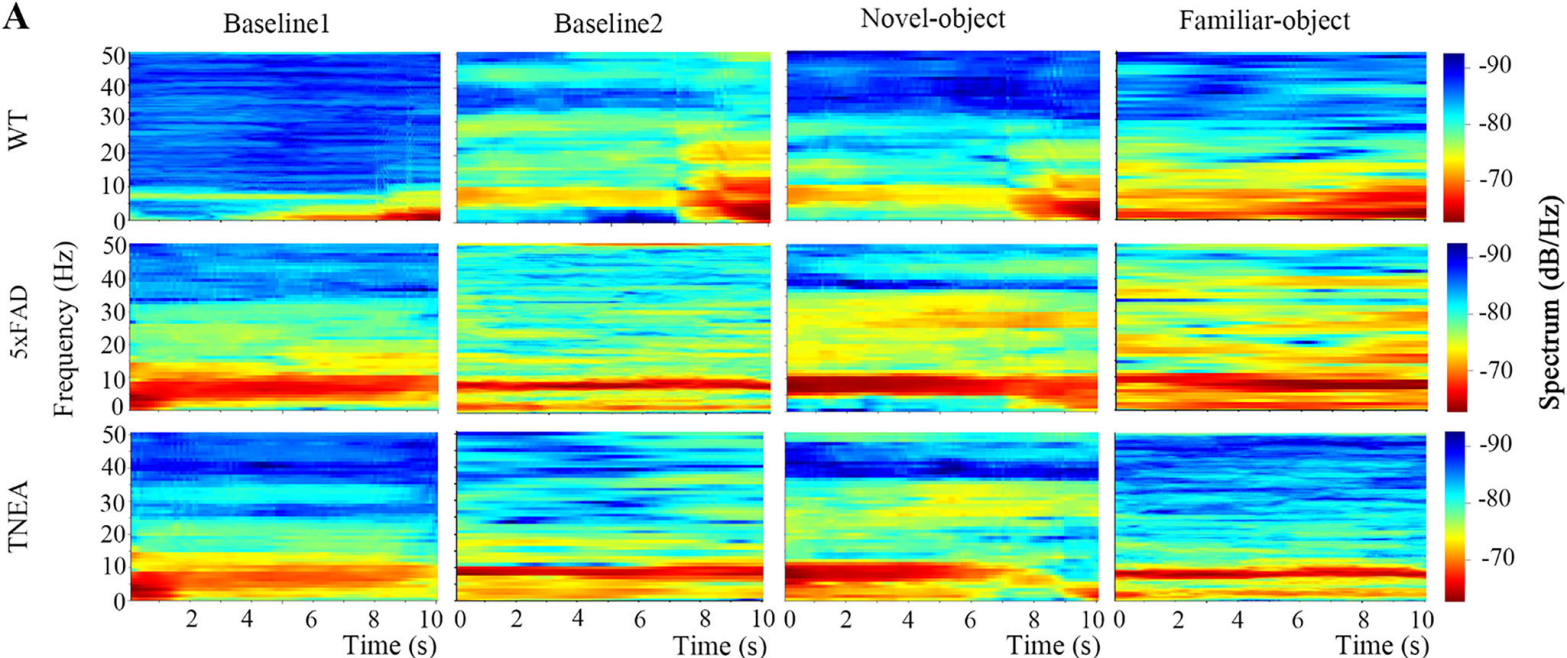


We apologize for this error.

