# Supplementary material for: TNEA Regulates Hippocampal Oscillation by Improving Inhibitory Synaptic Plasticity to Ameliorates Cognitive Impairment in Alzheimer's Disease
Source: Adv Sci (Weinh). 2025 Nov 11;13(3):e10885. doi: 10.1002/advs.202510885 (PMC12806385; doi:10.1002/advs.202510885)
Supplement: Supplementary file 1 — Supporting Information [file ADVS-13-e10885-s004.docx]

**TNEA Regulates Hippocampal Oscillation by Improving Inhibitory Synaptic Plasticity to Ameliorates Cognitive Impairment in Alzheimer’s Disease**

Zhongzhao Guo^1,2,3#^, Hong Ni^2,4#^, Yu Lu^2#^, Zhengyu Cui^6#^, Yixing Wang^6^, Zilu Zhu^2^, Xinyu Wei^2^ Chenyi Xia^2^, Ming Xu^2^, Lixia Du^2^, Yufang Yang^2^, Shi Shu^2^, Ke Wang^4^, Zhifei Wang^2^, Chunlei Shan^1,3,7*^, Deheng Wang^2,5*^

*1.* *Rehabilitation Center, Tongren Hospital, Shanghai Jiao Tong University School of Medicine, 200336, Shanghai, China.*

*2.* *School of Integrative Medicine, Shanghai University of Traditional Chinese Medicine, 201203, Shanghai, China.*

*3. Yuanshen Rehabilitation Institute, Shanghai Jiao Tong University School of Medicine, 200025, Shanghai, China.*

*4.* *Yueyang Hospital of Integrated Traditional Chinese and Western Medicine, Shanghai University of Traditional Chinese Medicine, 201203, Shanghai, China.*

*5.* *Engineering Research Center of Traditional Chinese Medicine Intelligent Rehabilitation, Shanghai University of Traditional Chinese Medicine, Shanghai 201203, China.*

*6. Department of Traditional Chinese Medicine, Shanghai East Hospital, Tongji University School of Medicine, Shanghai 201203, China.*

*7. Shanghai Key Laboratory of Flexible Medical Robotics, Tongren Hospital, Institute of Medical Robotics, Shanghai JiaoTong University, Shanghai, China*

^#^These authors contributed equally to this work.

*Corresponding authors.

**Correspondence:**

Deheng Wang, Ph.D

E-mail: [wangdeheng@shutcm.edu.cn](mailto:wangdeheng@shutcm.edu.cn)

**
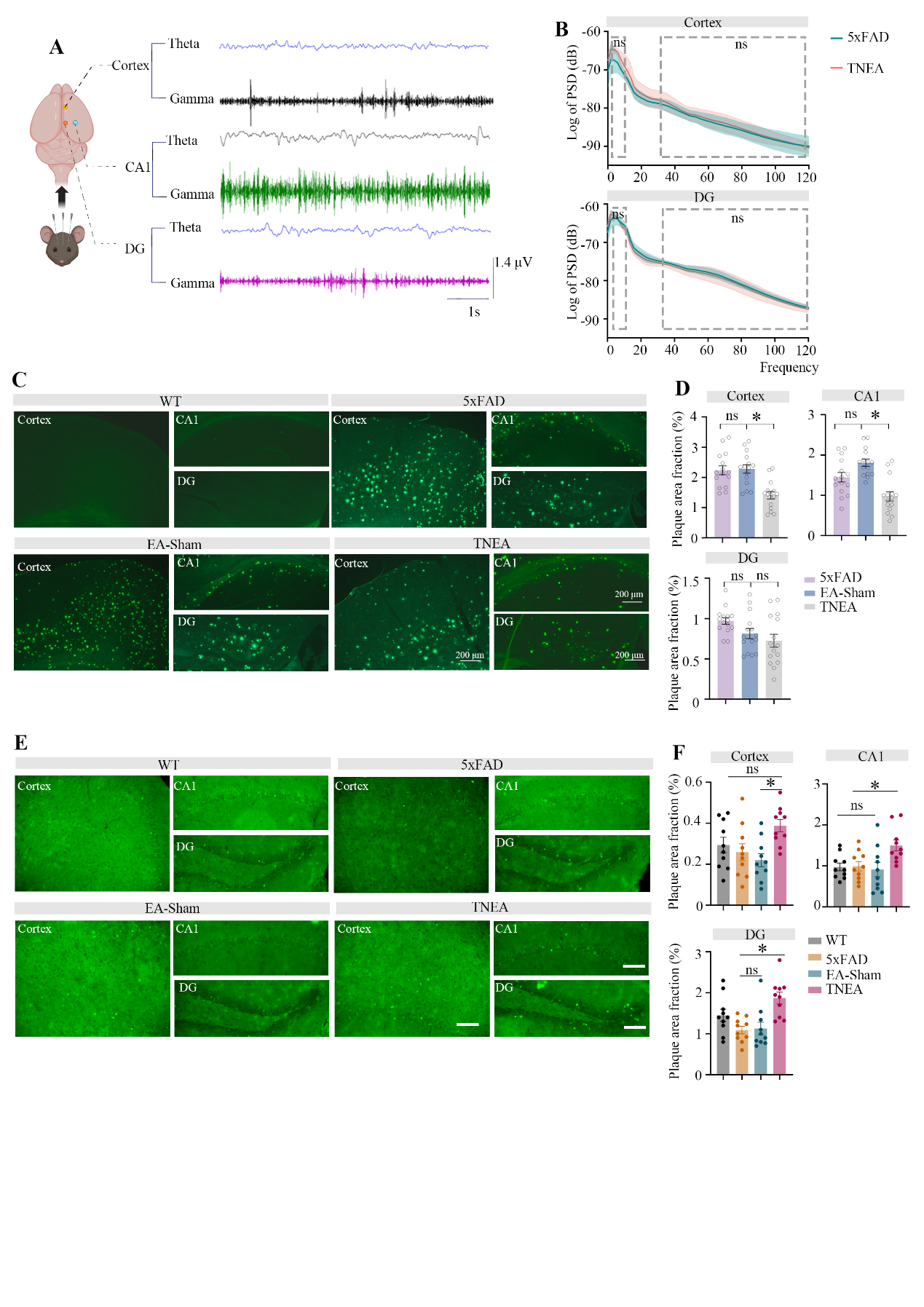
Figure S1**

**Figure S1.** **TNEA enhances cognitive function through its action in the CA1 region of the hippocampus.**

(A) Electrophysiological recording sites (including Cortex, CA1 and DG) and examples of representative theta and gamma oscillations. (B) Power spectral density of LFP from 2 groups. (C) Amyloid plaques labeled with thioflavin-S in the cortex, CA1 and DG of WT, 5×FAD, EA-Sham and TNEA mice. (D) Quantification of amyloid plaques in the cortex, CA1 and DG regions of WT, 5×FAD, EA-Sham and TNEA mice. (n = 10 slices per group) (E) Representative of c-Fos positive cells after NORT in the cortex, CA1 and DG regions. (F) Quantification of c-Fos positive cells in the cortex, CA1 and DG regions of WT, 5×FAD, EA-Sham and TNEA mice (n = 10 slices per group). Statistical significance was set at **P* < 0.05, one-way ANOVA with Tukey's multiple comparisons test, ns: not significant. All data are expressed as mean ± s.e.m.

**Figure S2**

**
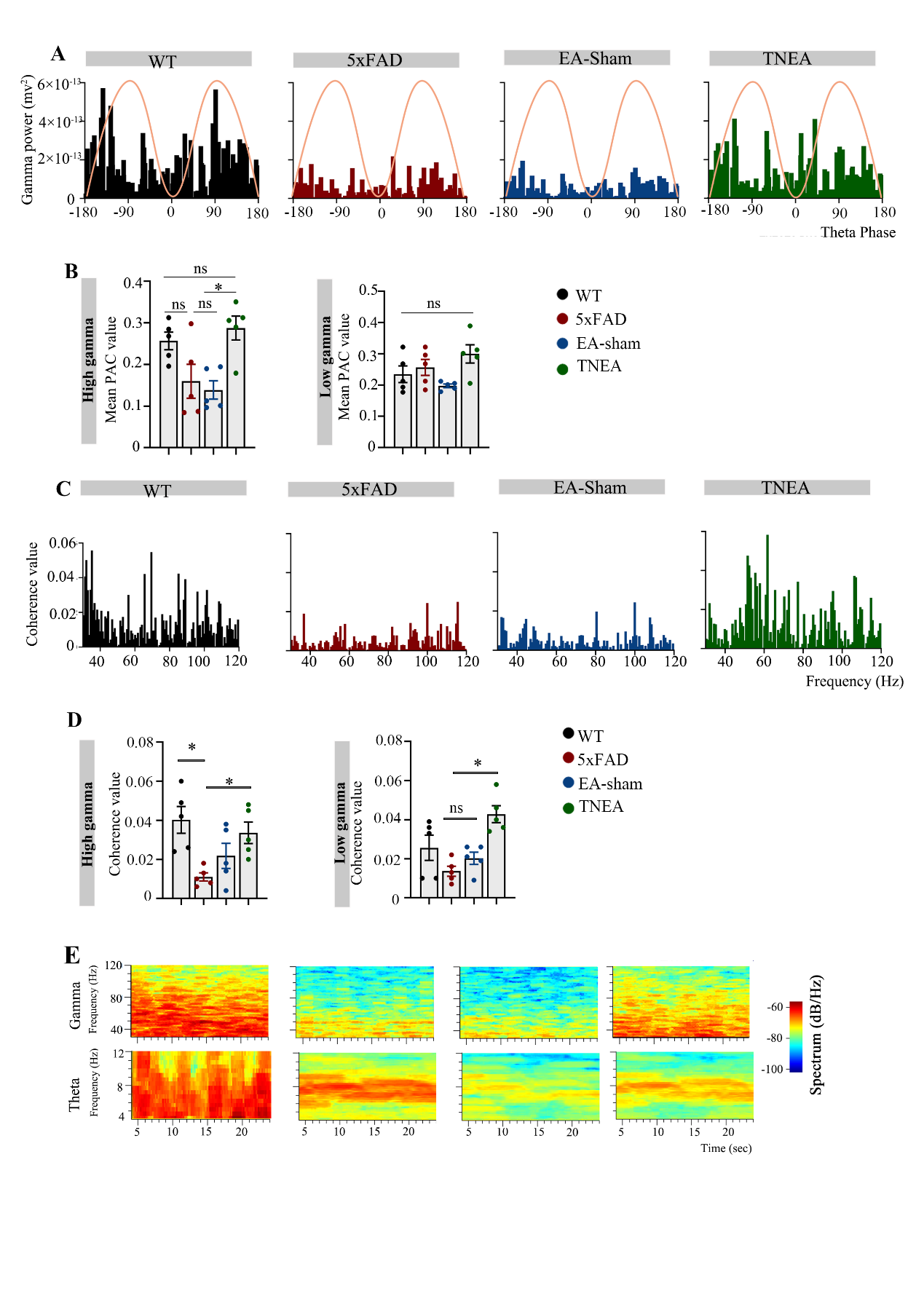
**

**Figure S2. Coherence changes in CA1 region**

(A) Phase-amplitude coupling (PAC) of gamma power over theta phase of 4 groups. (B) Mean PAC values of high (left)/low (right) gamma power over theta phase from 4 groups (n = 5 per group) (C) Coherence values of putative interneurons’ spikes and gamma oscillations of 4 groups. (D) Statistic analysis of coherence values between putative interneurons’ spikes and gamma oscillations in 4 groups (n = 5 per group). (E) Representative power spectrogram of gamma and theta oscillation from 4 groups. Statistical significance was set at **P* < 0.05, one-way ANOVA with Tukey's multiple comparisons test, ns: not significant. All data are expressed as mean ± s.e.m.

**Figure S3**

**
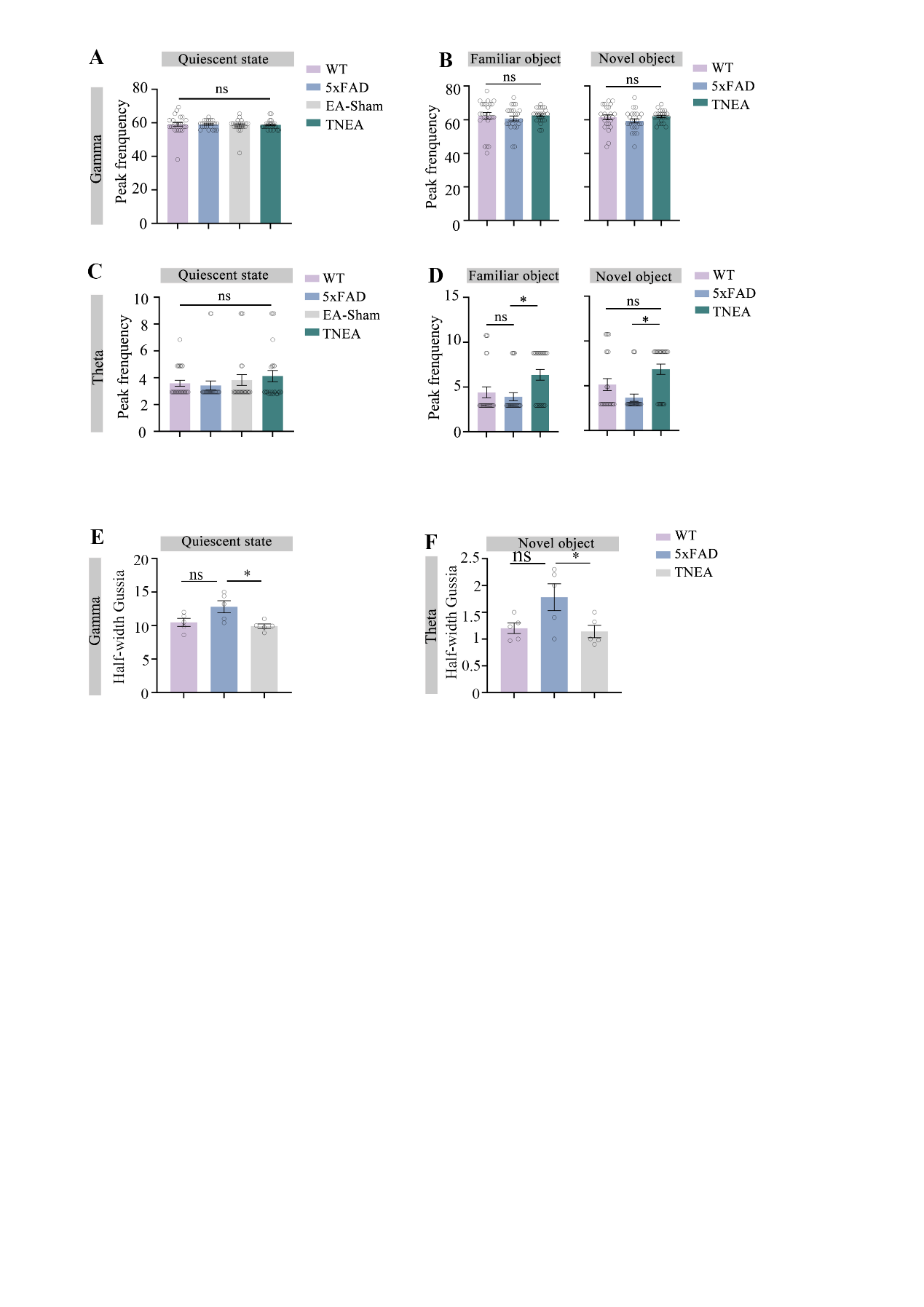
**

**Figure S3. Peak frequency and half-width changes in CA1 region.**

(A) Peak frequency of gamma oscillation from 4 groups during quiescent state (n = 3 per group) (B) Peak frequency of gamma oscillation from 4 groups during familiar object (left)/novel object (right) exploration (n = 3 per group). (C) Peak frequency of theta oscillation from 4 groups during quiescent state (n = 3 per group). (D) Peak frequency of theta oscillation from 4 groups during familiar object (left)/novel object (right) exploration (n = 3 per group). (E) Gamma oscillation’s half-width values of the Gaussian fitting from 3 groups during quiescent state (n = 5 per group). (F) Theta oscillation’s half-width values of the Gaussian fitting from 3 groups during novel object exploration (n = 5 per group). All data are expressed as mean ± s.e.m. Statistical significance was set at **P* < 0.05, one-way ANOVA with Tukey's multiple comparisons test, ns: not significant

**Figure S4**

**
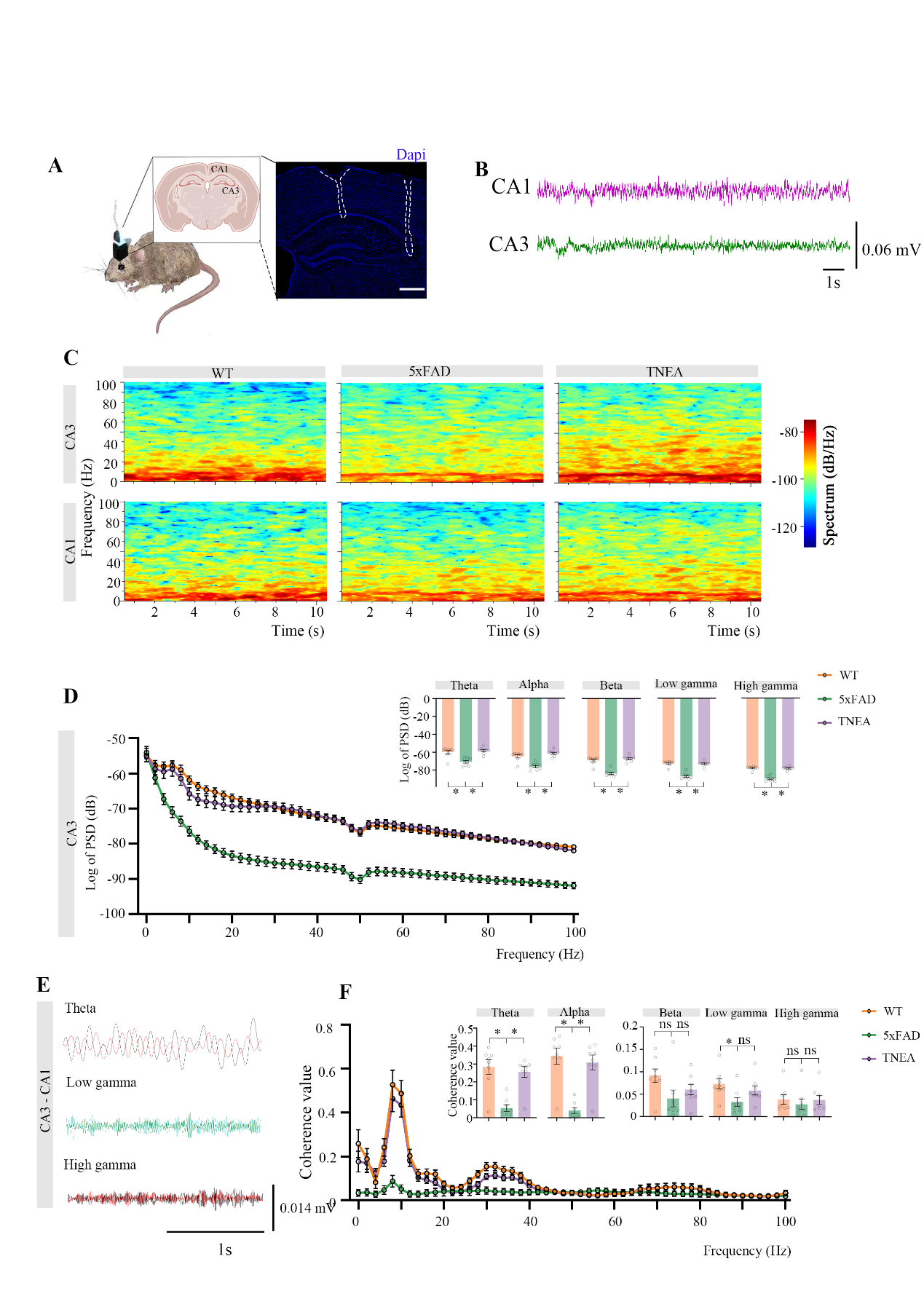
**

**Figure S4. TNEA improves the synchrony between the CA3 and CA1 brain regions**

(A) Tetrode insertion site (CA1 and CA3) of a mouse. Scale bar represents 500 μm. (B) Representative 1D view of LFP from CA1 and CA3. (C) Representative power spectrogram of broad-band oscillation from 3 groups. (D) PSD of broad-band oscillations from 3 groups in the CA1 region and PSD across frequency bands during quiescent state (upper panel) from 3 groups (n = 3 per group). (E) Examples of theta filtered CA3 and CA1 LFP recordings recorded during quiescent state. (F) Oscillation synchrony for CA3-CA1 LFPs during quiescent state (n = 3 per group). Statistical significance was set at **P* < 0.05, one-way ANOVA with Tukey's multiple comparisons test. ns: not significant. All data are expressed as mean ± s.e.m.

**Figure S5**

**
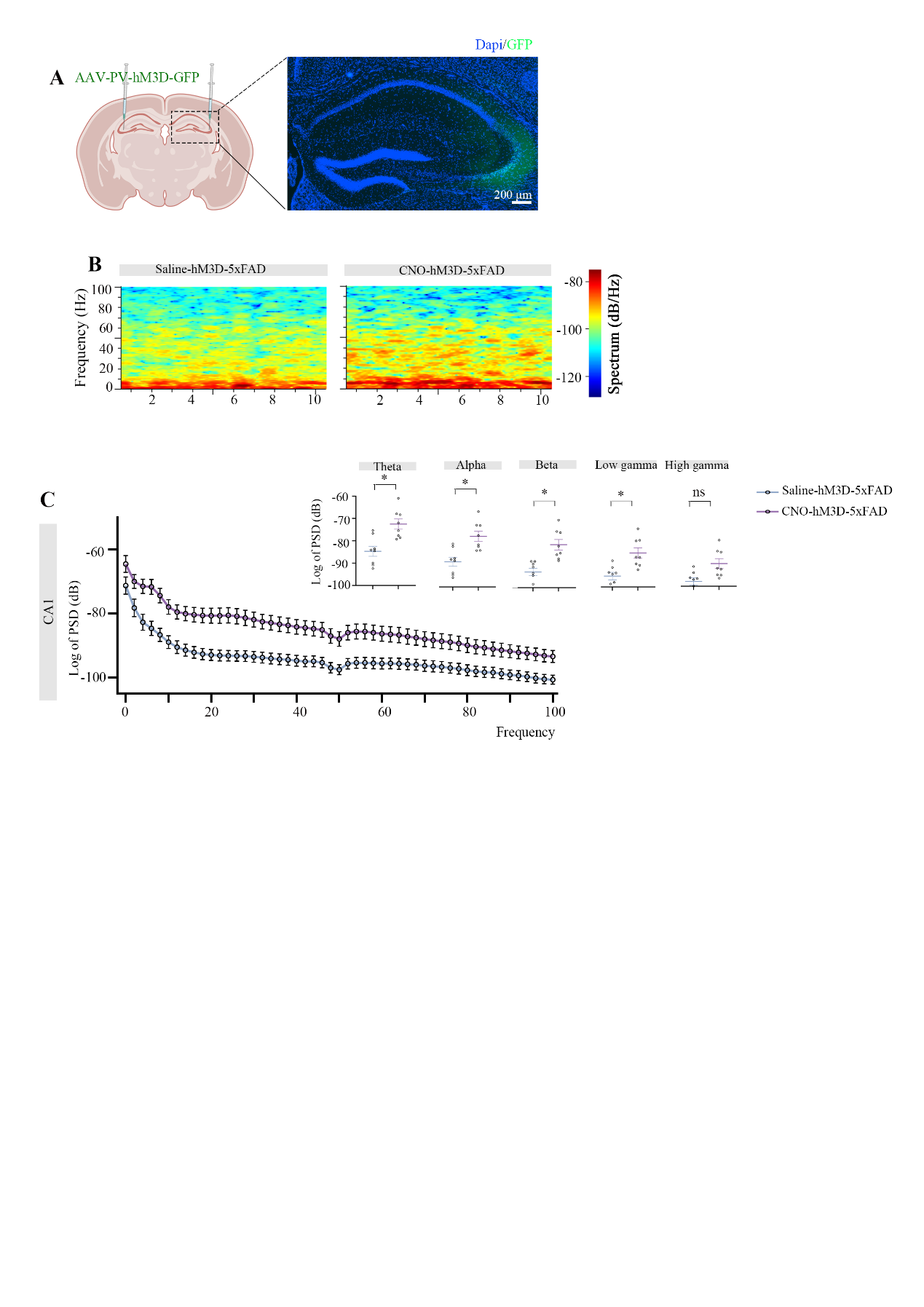
**

**Figure S5 Chemogenetic activation of the CA3 region enhances oscillatory power in the CA1 region.**

(A) Expression of hM3D-GFP in the CA3 of a mouse. Scale bar represents 200 μm. (B) Representative power spectrogram of broad-band oscillation from 2 groups. (C) PSD of broad-band oscillations from 2 groups in the CA1 region and PSD across frequency bands during quiescent state (upper panel) from 2 groups (n=3 per group). Statistical significance was set at **P* < 0.05, two tailed unpaired t-test, ns: not significant. All data are expressed as mean ± s.e.m.

**Figure S6**

**
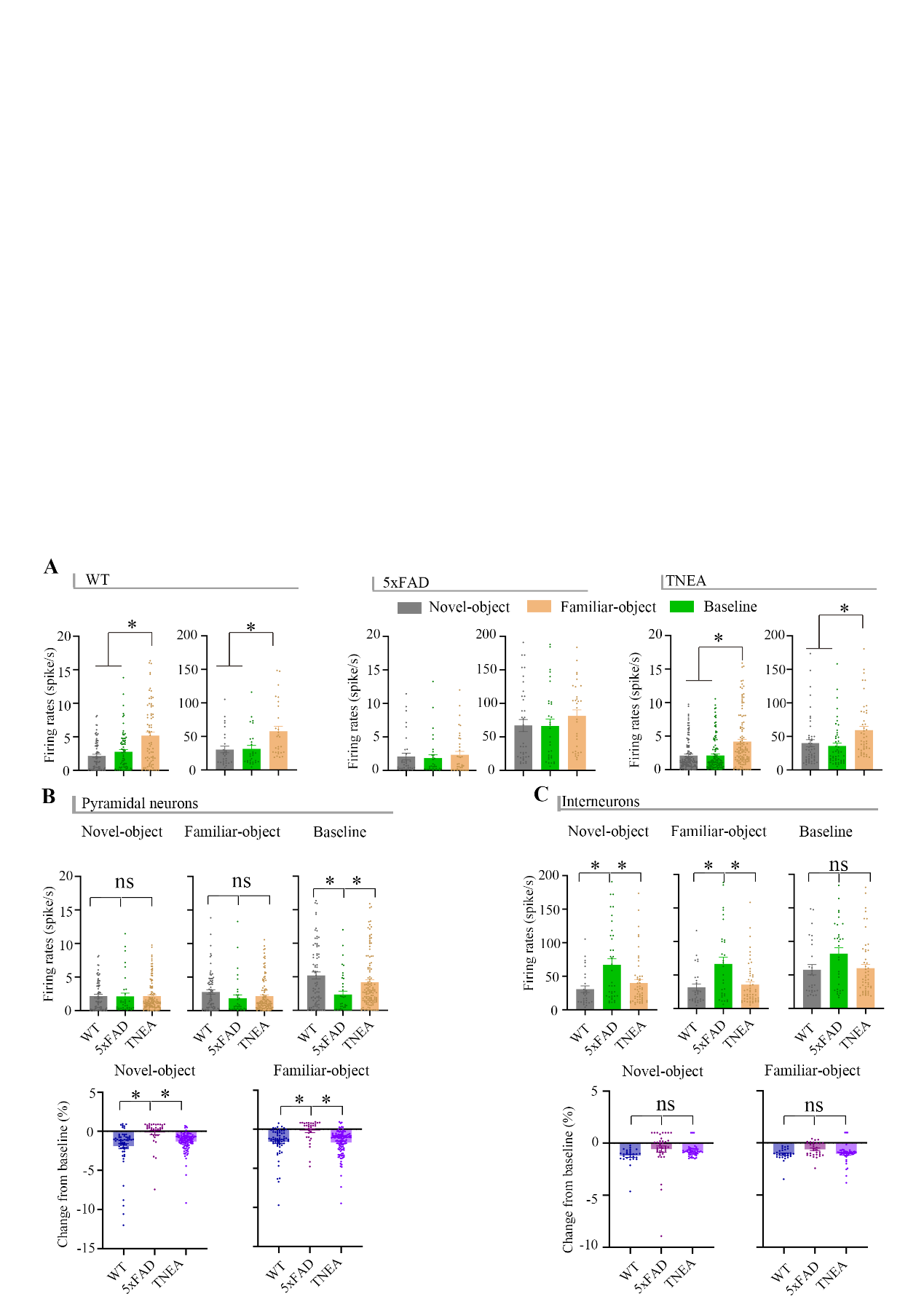
**

**Figure S6. Firing rates changes in putative interneurons and pyramidal neurons in WT, 5xFAD, and TNEA group**

(A) Mean firing rates of pyramidal neurons during habituation stage (baseline1), the stage of exploring novel object, and the stage of exploring familiar object (top). Changes of firing rates in pyramidal neurons in each group during the stage of exploring novel and familiar object, and the stage of exploring familiar object (bottom). (n = 61 for WT, n = 34 for 5xFAD, n=116 for TNEA). (B) Mean firing rates of interneurons during habituation stage (baseline1), the stage of exploring novel object, and the stage of exploring familiar object (top). Changes of firing rates in interneurons in each group during the stage of exploring novel object (novel), and the stage of exploring familiar object (familiar) (bottom) (n = 28 for WT, n = 31 for 5xFAD, n = 49 for TNEA). (C) Mean firing rates of neurons in the WT 5xFAD and TNEA groups of mice during the stage of exploring novel object, the stage of exploring familiar object and the stage of exploring other arena(baseline2). (pyramidal neurons: n = 61 for WT, n = 34 for 5xFAD, n = 116 for TNEA, interneurons: n = 28 for WT, n = 31 for 5xFAD, n = 49 for TNEA). All data are expressed as mean ± s.e.m. Statistical significance was set at **P* < 0.05, one-way ANOVA with Tukey's multiple comparisons test, ns: not significant.

**Figure S7**


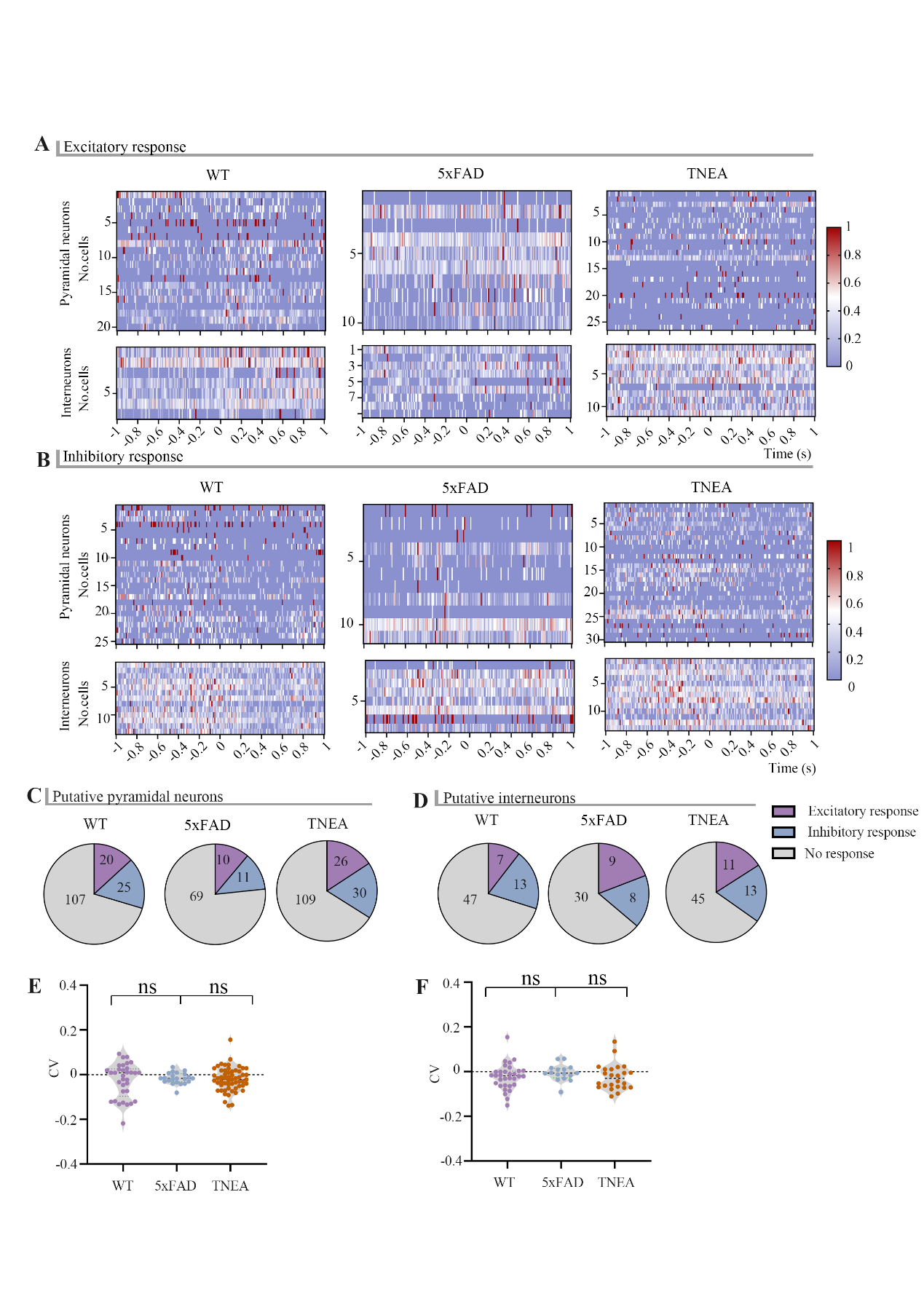


**Figure S7. Changes in putative interneurons and pyramidal neurons in WT, 5xFAD, and TNEA group**

(A) Representative firing rates of excitatory responses in WT, 5xFAD, and TNEA groups before and after the exploration onset (normalized firing rates were calculated by dividing the maximum firing rate by the firing rate for each unit). Top: putative pyramidal neurons, bottom: putative interneurons. Color codes are used to indicate low (blue) to high (red) firing activity. (B) Representative firing rates of inhibitory responses in the WT, 5xFAD, and TNEA groups before and after the exploration onset (normalized firing rates were calculated by dividing firing rates by the maximum firing rate of each unit). Top: putative pyramidal neurons, bottom: putative interneurons. Color-coded to indicate low (blue) to high (red) firing activity. (C-D) Number of neurons that have different responses to familiar-object exploring in each group. (C: pyramidal neurons, D: interneurons). Excitatory, no response, and inhibitory response are indicated in purple, blue, and gray, respectively. (E-F) The rate of change (CV) of the mean firing rate in neurons recorded before and after the exploration onset in three groups of mice. The value of each neuron has been normalized (Norm.) to its maximum firing rate (E: putative pyramidal neurons, F: putative interneurons). Data are mean ± SEM (putative pyramidal neurons: n = 152 for WT, n = 90 for 5xFAD, n = 165 for TNEA, putative interneurons: n = 67 for WT, n=47 for 5xFAD, n = 69 for TNEA). All data are expressed as mean ± s.e.m. Statistical significance was set at **P* < 0.05, one-way ANOVA with Tukey's multiple comparisons test, ns: not significant.

**Figure S8**


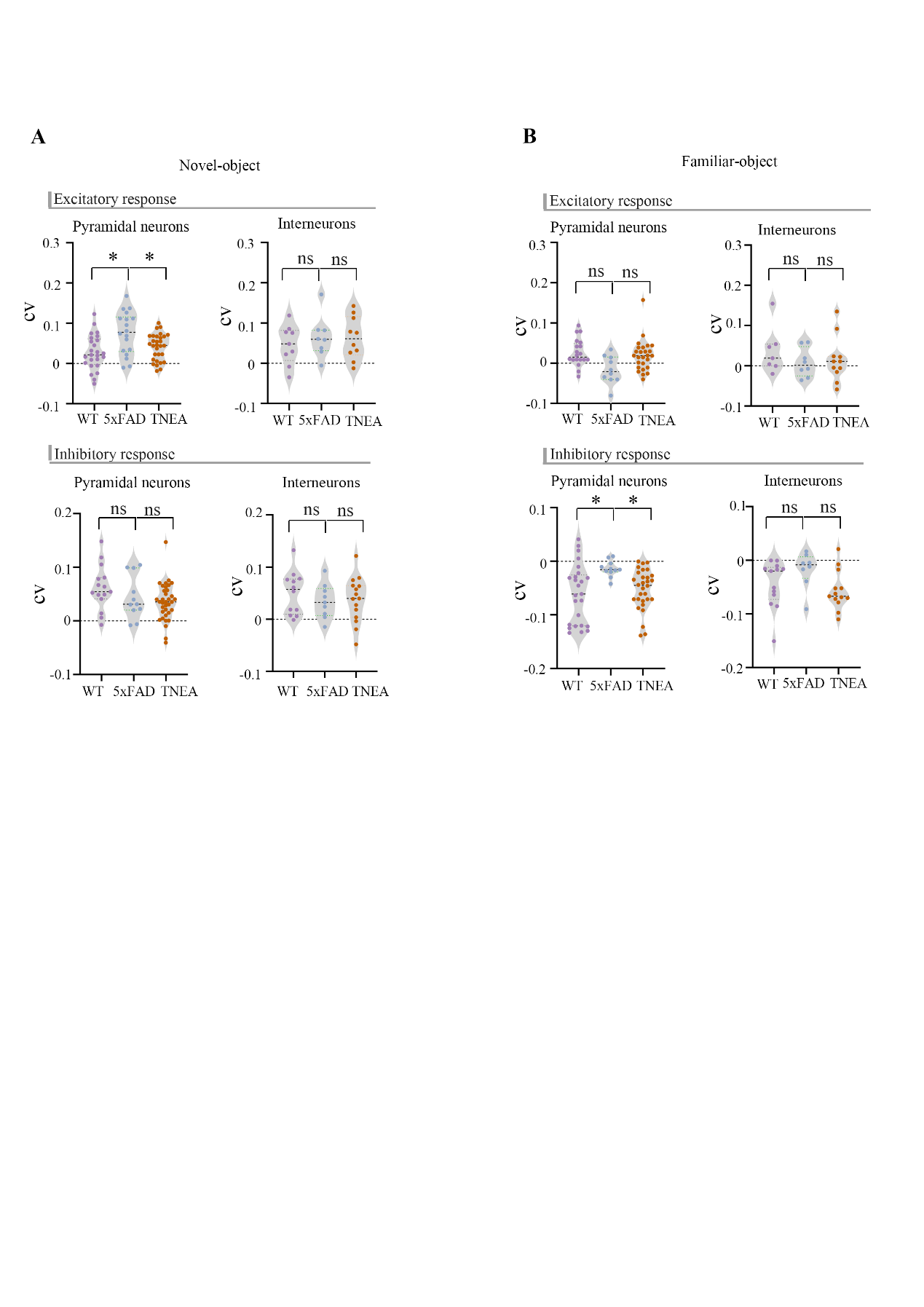


**Figure S8.** **Firing rates changes in putative interneurons and pyramidal neurons in WT, 5xFAD, and TNEA group of mice**

(A) Coefficient of variation (CV) in the mean neuronal firing rate of excitatory (top)/inhibitory (bottom) responses recorded before and after the onset of novel object exploration in three groups of mice. The values of each neuron have been normalized (Norm.) to its maximum firing rate (Top: putative pyramidal neurons: n = 25 for WT, n = 18 for 5xFAD, n = 30 for TNEA, putative interneurons: n = 9 for WT, n = 8 for 5xFAD, n = 10 for TNEA Bottom: putative pyramidal neurons: n = 15 for WT, n=11 for 5xFAD, n = 35 for TNEA, putative interneurons: n = 12 for WT, n = 9 for 5xFAD, n = 15 for TNEA). (B) The rate of change (CV) in the mean neuronal firing rate of excitatory (top) /inhibitory (bottom) responses recorded before and after the onset of familiar object exploration in three groups of mice. The values of each neuron have been normalized (Norm.) to its maximum firing rate. Data are mean ± SEM (Top: putative pyramidal neurons: n = 20 for WT, n = 10 for 5xFAD, n = 26 for TNEA, putative interneurons: n = 7 for WT, n = 9 for 5xFAD, n = 11 for TNEA. Bottom: putative pyramidal neurons: n = 25 for WT, n = 11 for 5xFAD, n = 30 for TNEA, putative interneurons: n = 13 for WT, n = 8 for 5xFAD, n = 13 for TNEA). All data are expressed as mean ± s.e.m. Statistical significance was set at **P* < 0.05, one-way ANOVA with Tukey's multiple comparisons test, ns: not significant.

**Figure S9**


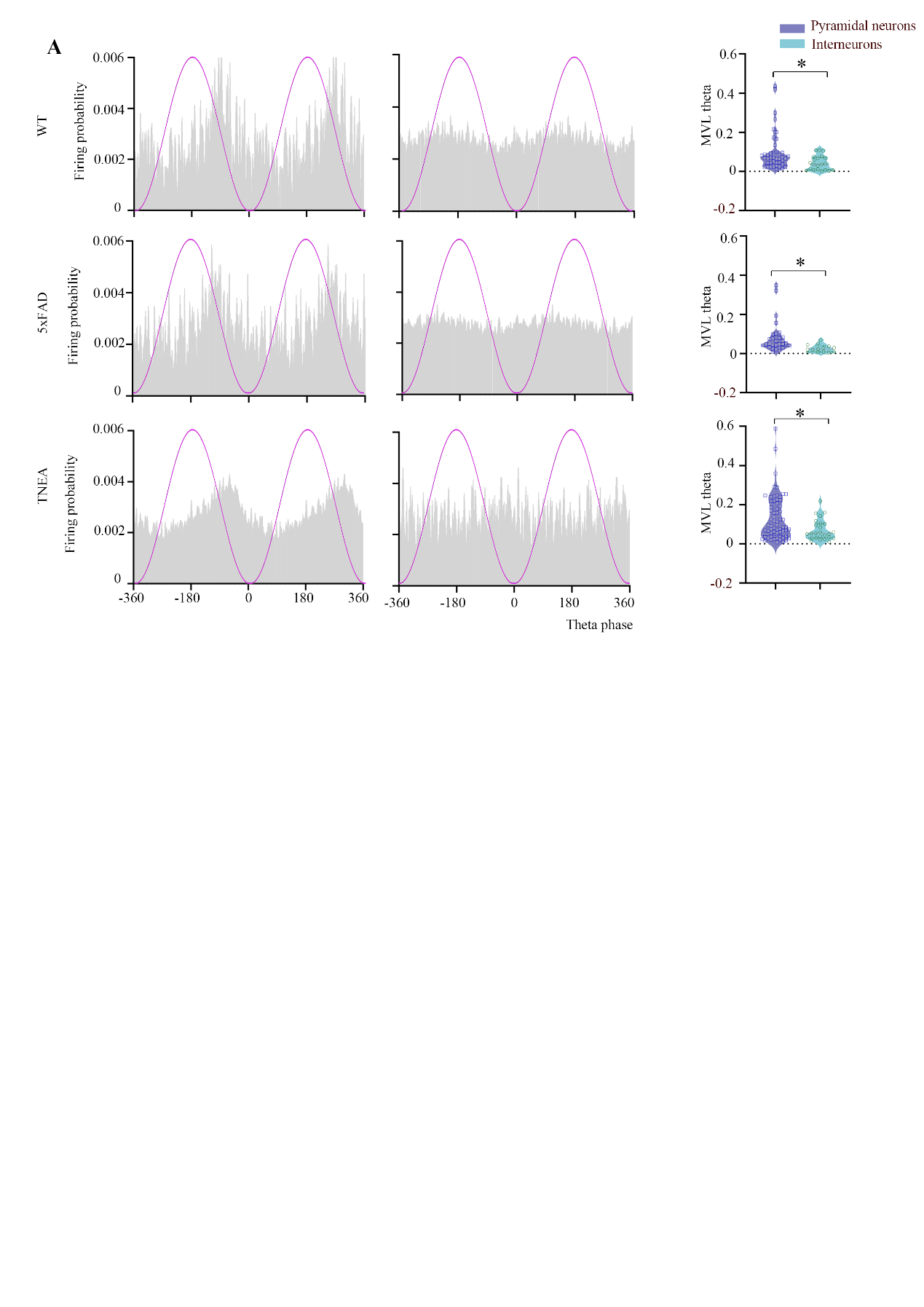


**Figure S9. Firing dynamics in 5xFAD mice are normal**

(A) Representative plots of the spike distribution of putative pyramidal neurons and interneurons with different phases of theta oscillation in three groups of mice (Left and middle panels). Plots of the mean vector length (MVL) of the putative pyramidal neurons compared to the putative interneurons in three groups of mice (putative pyramidal neurons: n = 40 for WT, n = 29 for 5xFAD, n = 65 for TNEA, putative interneurons: n = 21 for WT, n = 14 for 5xFAD, n = 25 for TNEA). All data are expressed as mean ± s.e.m. Statistical significance was set at **P* < 0.05, Unpaired t test, ns: not significant.

**Figure S10**


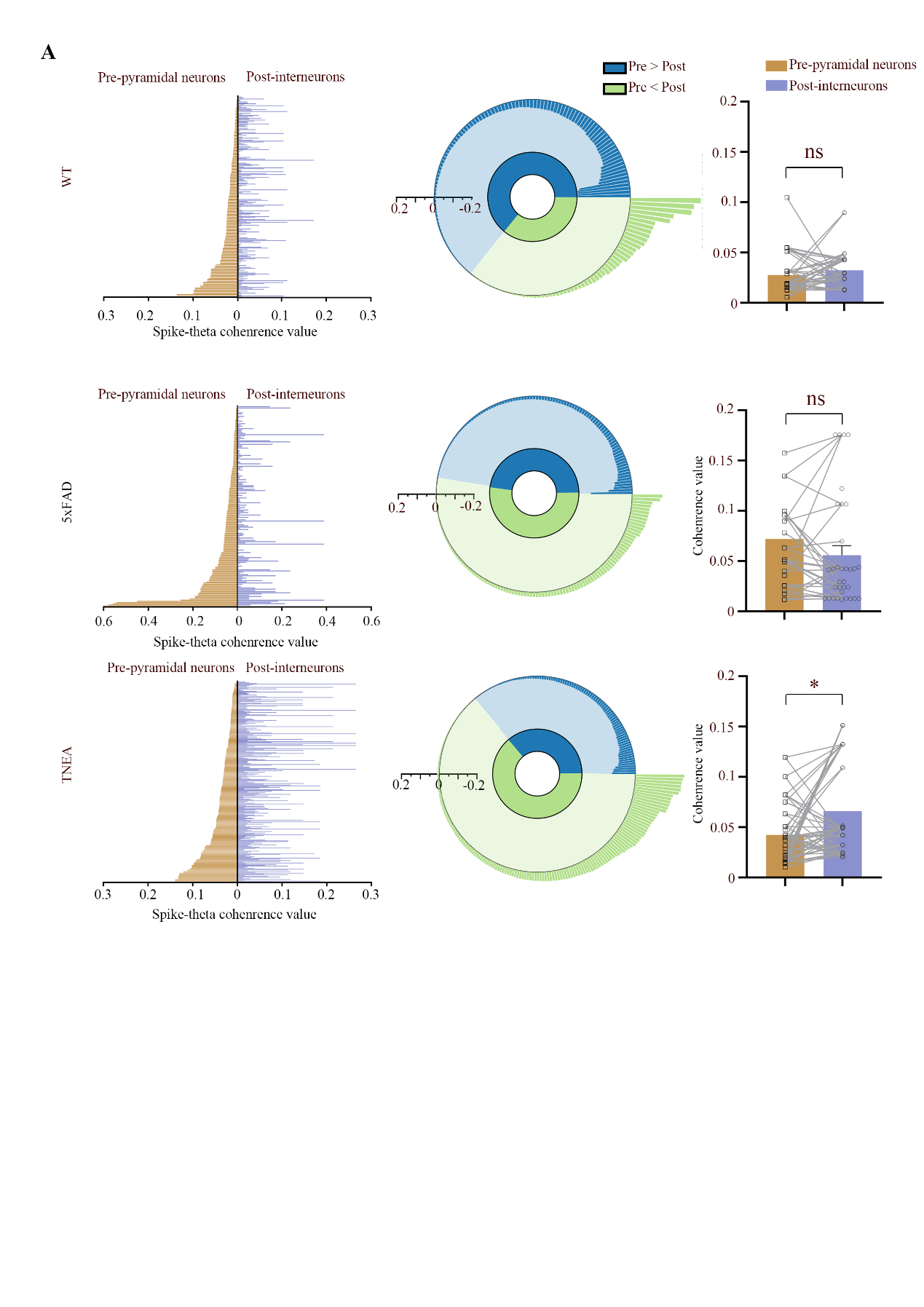


**Figure S10. TNEA treatment has no effect on excitatory monosynaptic connections during NORT** (A) correlation between the rate histogram of every PY-to-IN cell pair with theta oscillation in mice of WT, 5xFAD, TNEA groups (left). Correlation difference of every presynaptic pyramidal neurons - postsynaptic interneurons cell pairs, blue: postsynaptic interneurons with theta oscillation have a greater coherence value than presynaptic pyramidal neurons with theta oscillation, green: postsynaptic interneurons with theta oscillation have a greater coherence value is greater than the presynaptic pyramidal neurons with theta oscillation (middle). Mean coherence value of each group of cell pairs with theta oscillation (Right) (n = 44 pairs for WT, n = 42 pairs for 5xFAD, n = 51 pairs for TNEA). All data are expressed as mean ± s.e.m. Statistical significance was set at **P* < 0.05, paired t test, ns: not significant.

**Figure S11**

**
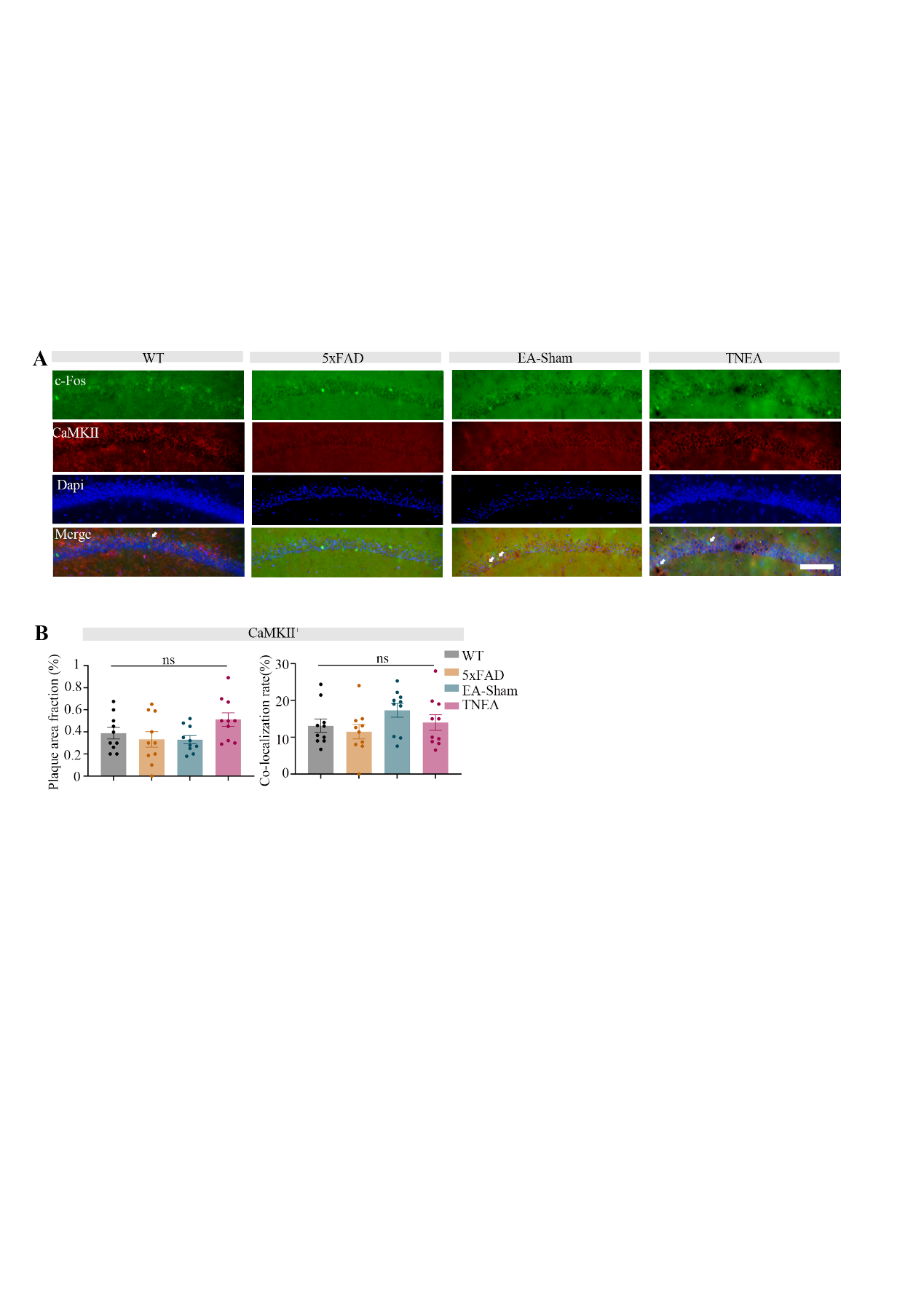
**

**Figure S11 TNEA's effects are independent of pyramidal neurons.**

**(**A) Shown are representative images for immunohistochemical staining of c-Fos (first row; green) staining in CaMKⅡ+ neurons (second row; red), Dapi (the third row, blue) and their colocalization (last row) in the CA1 region of 4 groups. (B) Quantification of CaMKⅡ+ fluorescence intensity (Left) and the percent colocalization of the c-Fos with PV in the CA1(n = 10 slices per group). All data are expressed as mean ± s.e.m. Statistical significance was set at **P* < 0.05, one-way ANOVA with Tukey's multiple comparisons test, ns: not significant.

**Figure S12**

**
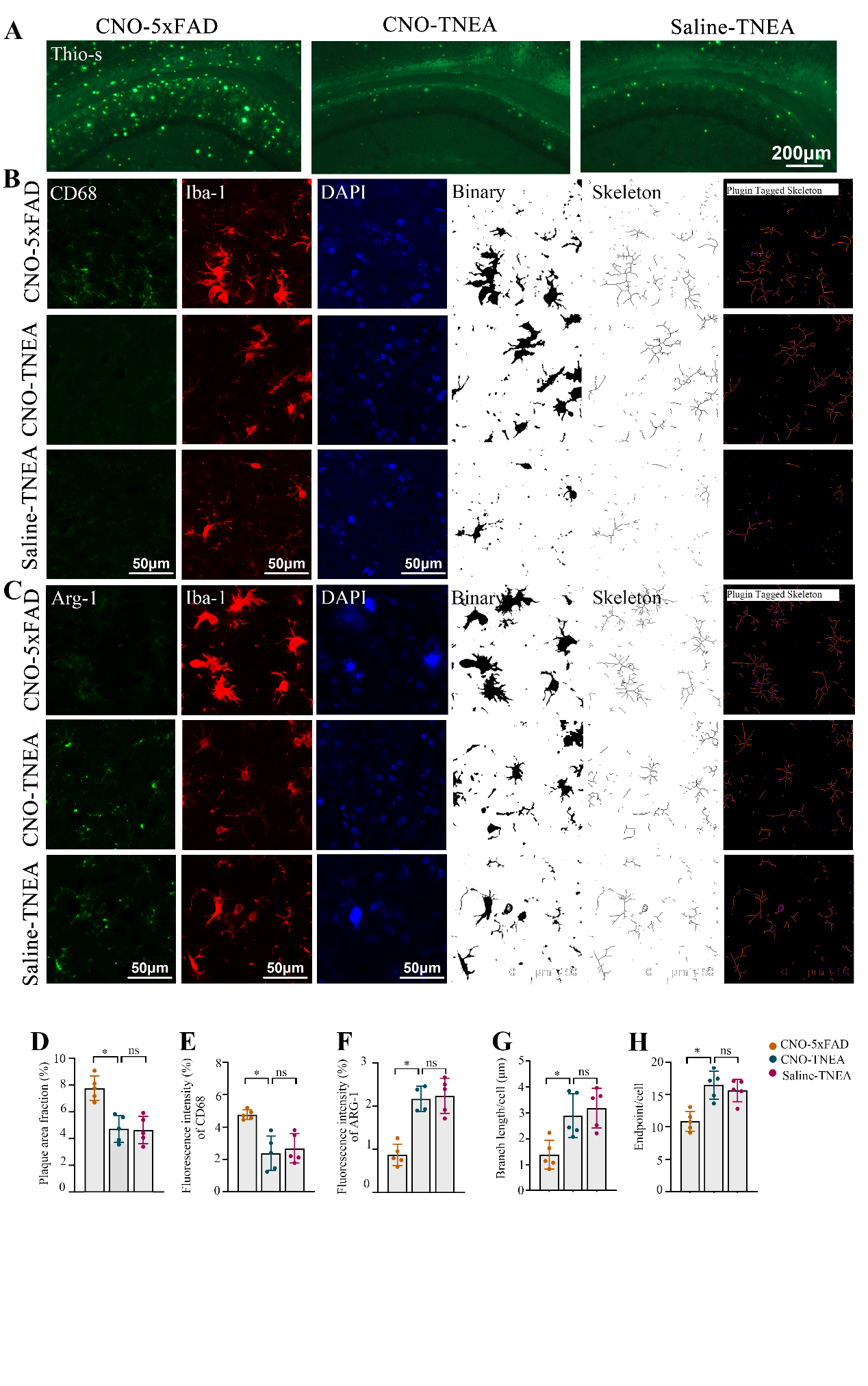
**

**Figure S12. PV interneuron did not influence the effects in relieving Aβ deposit and promoting microglial phenotypic transition in the hippocampal CA1 of 5xFAD mice by TNEA**

(A) Representative micrographs of amyloid plaques labeled with thioflavin S in the hippocampal CA1 of 3 groups. (B) Representative double immunofluorescence micrographs of Iba-1(red) and CD68 (green) in the hippocampal CA1 of different groups and skeleton analysis of Iba-1^+^ microglia morphologies. (C) Representative double immunofluorescence micrographs of Iba-1(red) and Arg-1 (green) in the hippocampal CA1 of different groups and skeleton analysis of Iba-1^+^ microglia morphologies. (D) Quantification of amyloid plaque (n = 5 mice per group). (E) Quantification of CD68^+^ fluorescence intensity (n = 5 mice per group). (F) Quantification of Arg-1^+^ fluorescence intensity (n = 5 mice per group). (G) Quantitative analysis of microglia process length/cell (n = 5 mice per group). (H) Quantitative analysis of microglia endpoints/cells in the hippocampus of different groups (n = 5 per group). All data are expressed as mean ± s.e.m. Statistical significance was set at **P* < 0.05, one-way ANOVA with Tukey's multiple comparisons test, ns: not significant

**Figure S13**

**
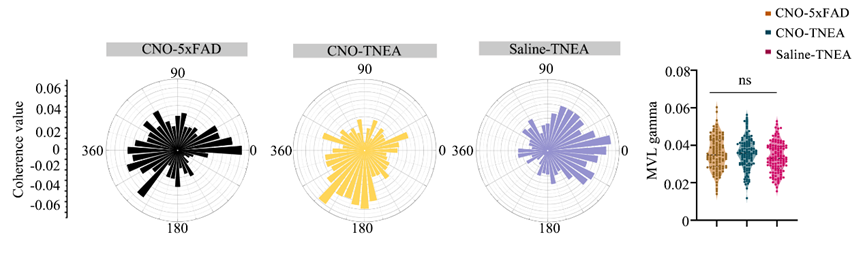
**

**Figure S13**

(A) Left: representative polar plots of putative pyramidal neurons’ spikes distributions along the gamma phase from 4 groups. Right: MVL of 3 groups (n = 109 for CNO-5xFAD, n = 114 for CNO-TNEA, n = 117 for Saline-TNEA). Statistical significance was set at **P* < 0.05, one-way ANOVA with Tukey's multiple comparisons test, ns: not significant. All data are expressed as mean ± s.e.m.

**Figure S14**

**
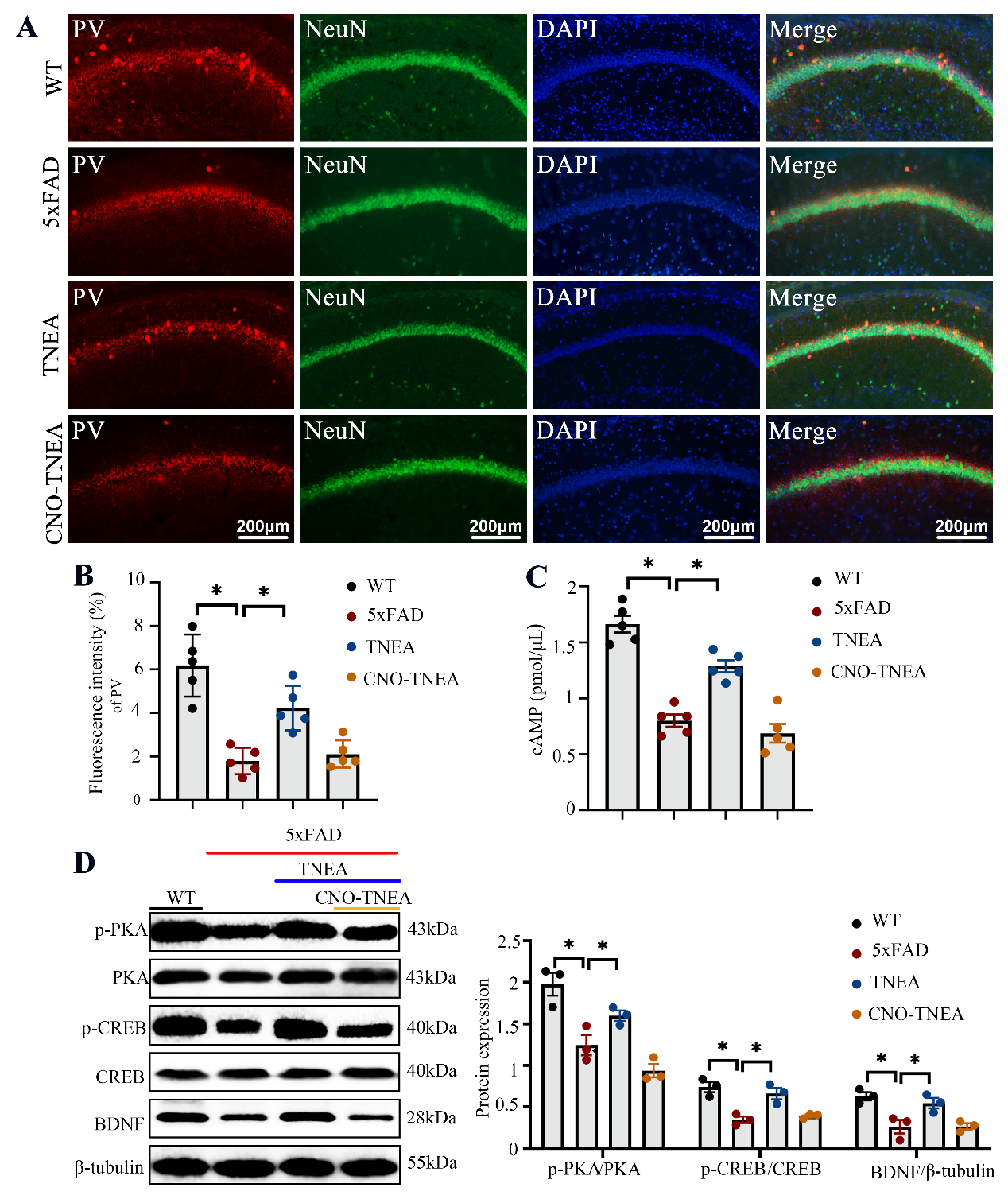
**

**Figure S14.** **TNEA rescued the PV interneurons loss and activated BDNF by cAMP/PKA/CREB pathway in the hippocampus of 5xFAD mice**

(A) Representative double immunofluorescence micrographs of PV interneurons (red) and NeuN (green) in the hippocampal CA1 of different groups. (B) Quantification of PV interneurons (+) fluorescence intensity (n = 5 mice per group). (C) cAMP levels in the hippocampus of different groups tested by ELISA (n = 5 mice per group **P* < 0.05). (D) p-PKA, PKA, p-CREB, 964 CREB, and BDNF proteins expression in the hippocampus of different groups detected by Western Blot (n = 3 mice per group). All data are expressed as mean ± s.e.m. Statistical significance was set at **P* < 0.05, one-way ANOVA with Tukey's multiple comparisons test t, ns: not significant.

**Figure S15**


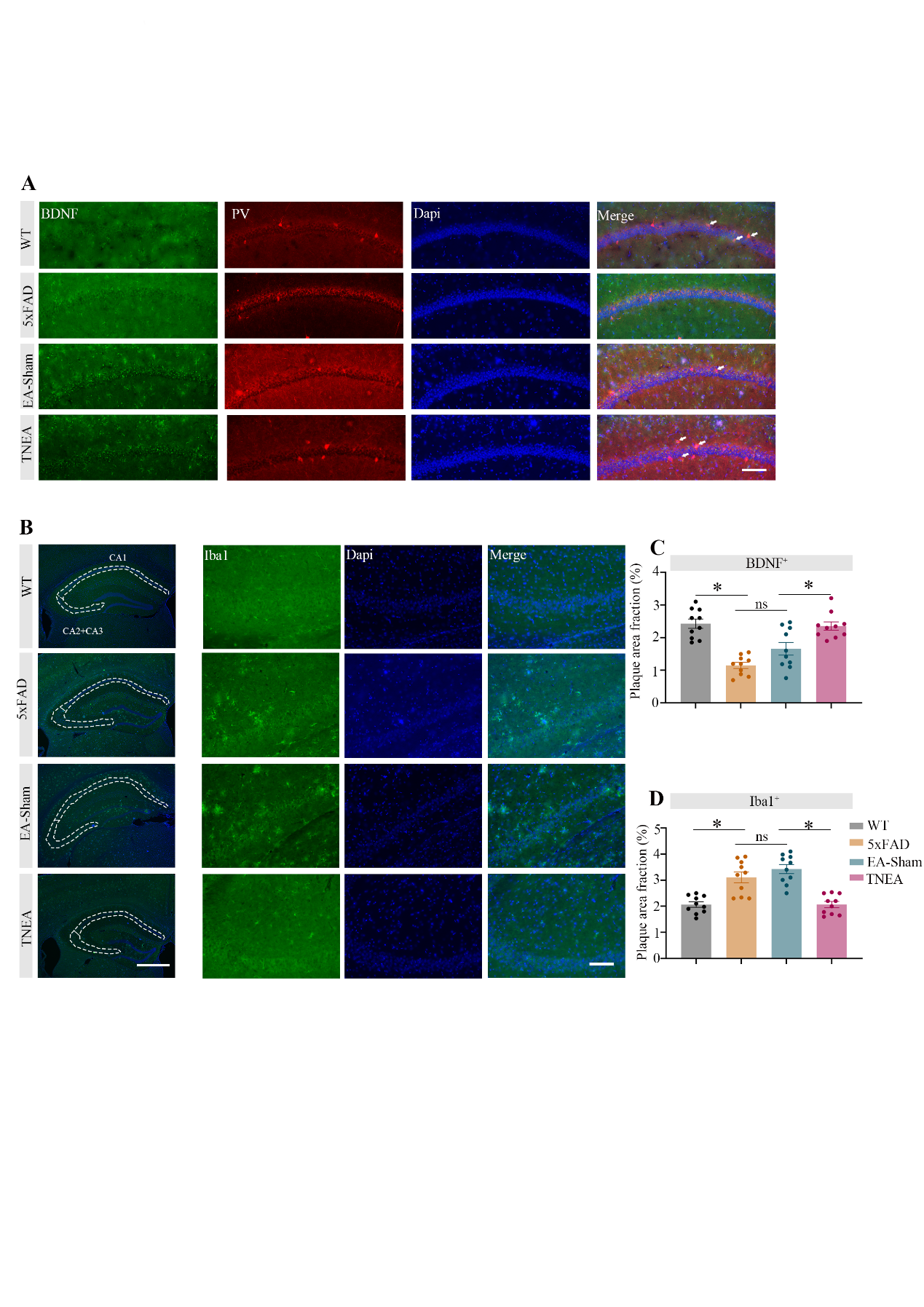


**Figure S15. TNEA activated BDNF in CA1 region and inhibited microglia activition in the CA3 region.**

(A) Shown are representative images for immunohistochemical staining of BDNF (first column; green) staining in PV-positive neurons (second column; red), Dapi (the third column, blue) and their colocalization (last column) in the CA1 region of 4 groups. (B) Shown are representative images for immunohistochemical staining of Iba1 (second column; green), Dapi (the third column, blue) and their colocalization (last column) in the CA3 region of 4 groups. (C) Quantification of BDNF+ fluorescence intensity in the CA3 (n = 5 mice per group). (D) Quantification of Iba1+ fluorescence intensity in the CA3 (n = 5 mice per group). Statistical significance was set at **P* < 0.05, one-way ANOVA with Tukey's multiple comparisons test, ns: not significant. All data are expressed as mean ± s.e.m

**Figure S16**


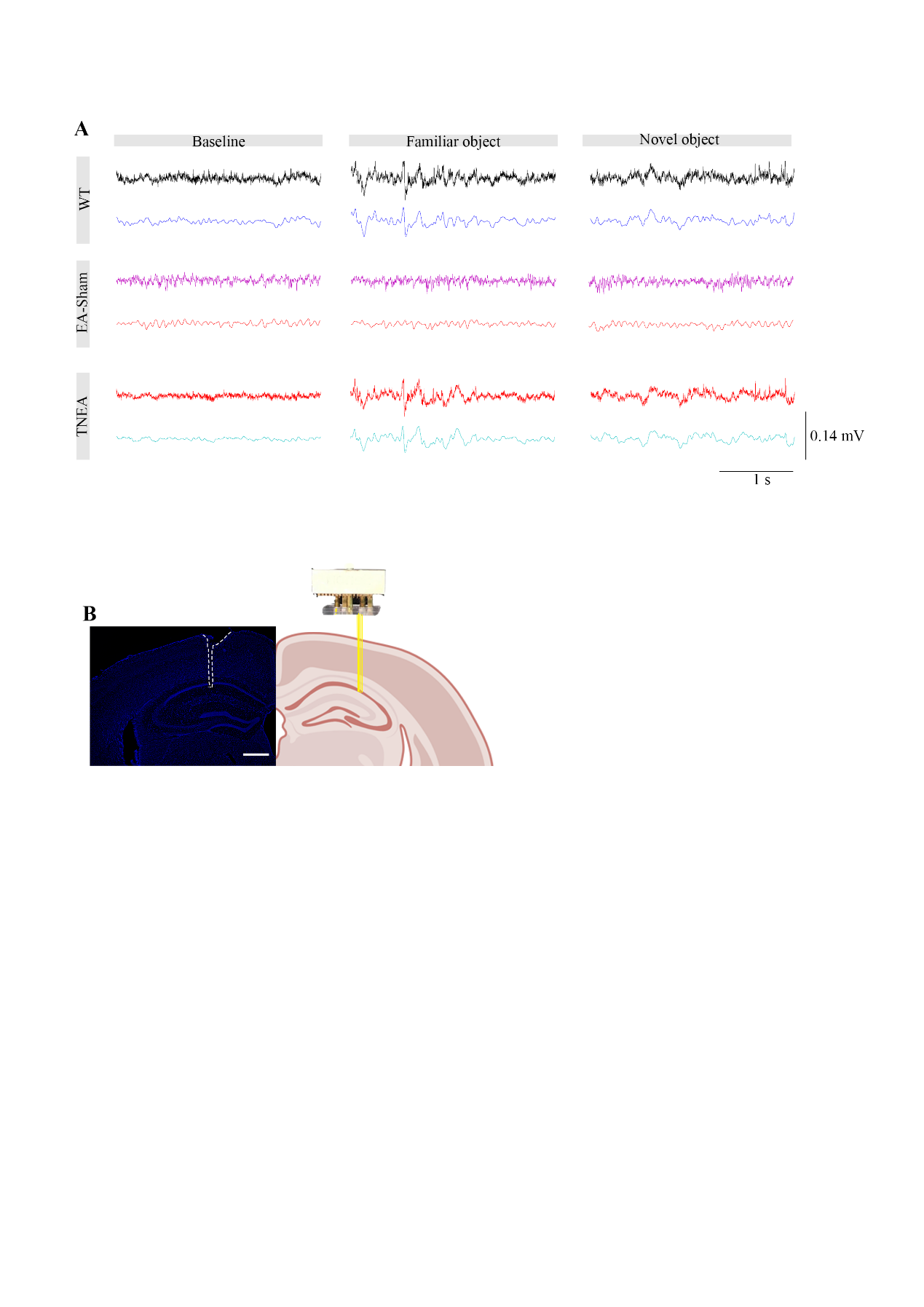


**Figure S16**

(A) Representative 1D view of LFP filtered gamma and theta oscillation from 3 groups during NORT. (B) Tetrode insertion site (CA1) of a mouse. Scale bar represents 500 μm.
