# Supplementary figures and images for: TNEA Regulates Hippocampal Oscillation by Improving Inhibitory Synaptic Plasticity to Ameliorates Cognitive Impairment in Alzheimer's Disease
Source: Adv Sci (Weinh). 2025 Nov 11;13(3):e10885. doi: 10.1002/advs.202510885 (PMC12806385; doi:10.1002/advs.202510885)

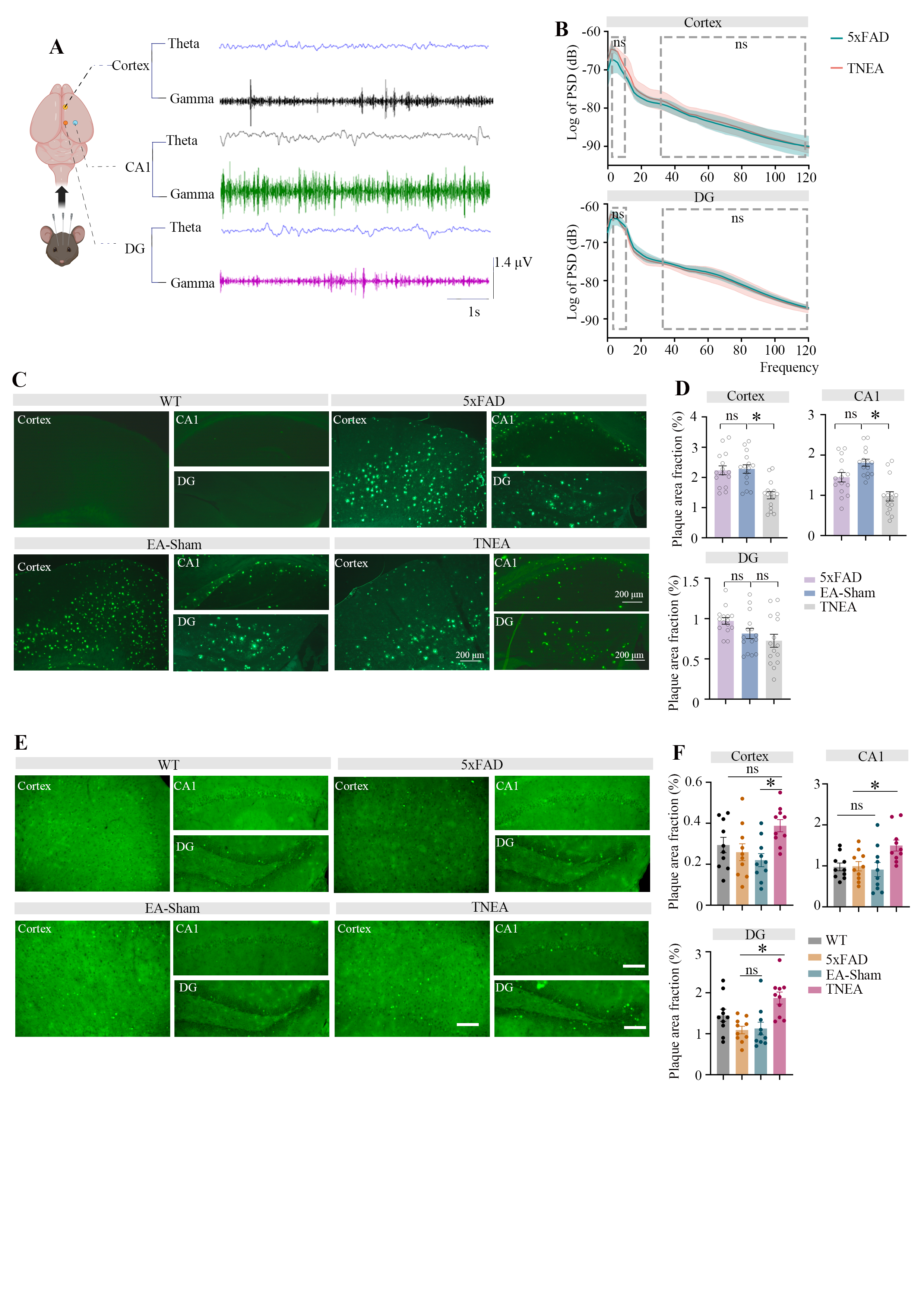

Supplement: Supplementary file 2 — Supplemental Figure 1 [file ADVS-13-e10885-s011.tif]

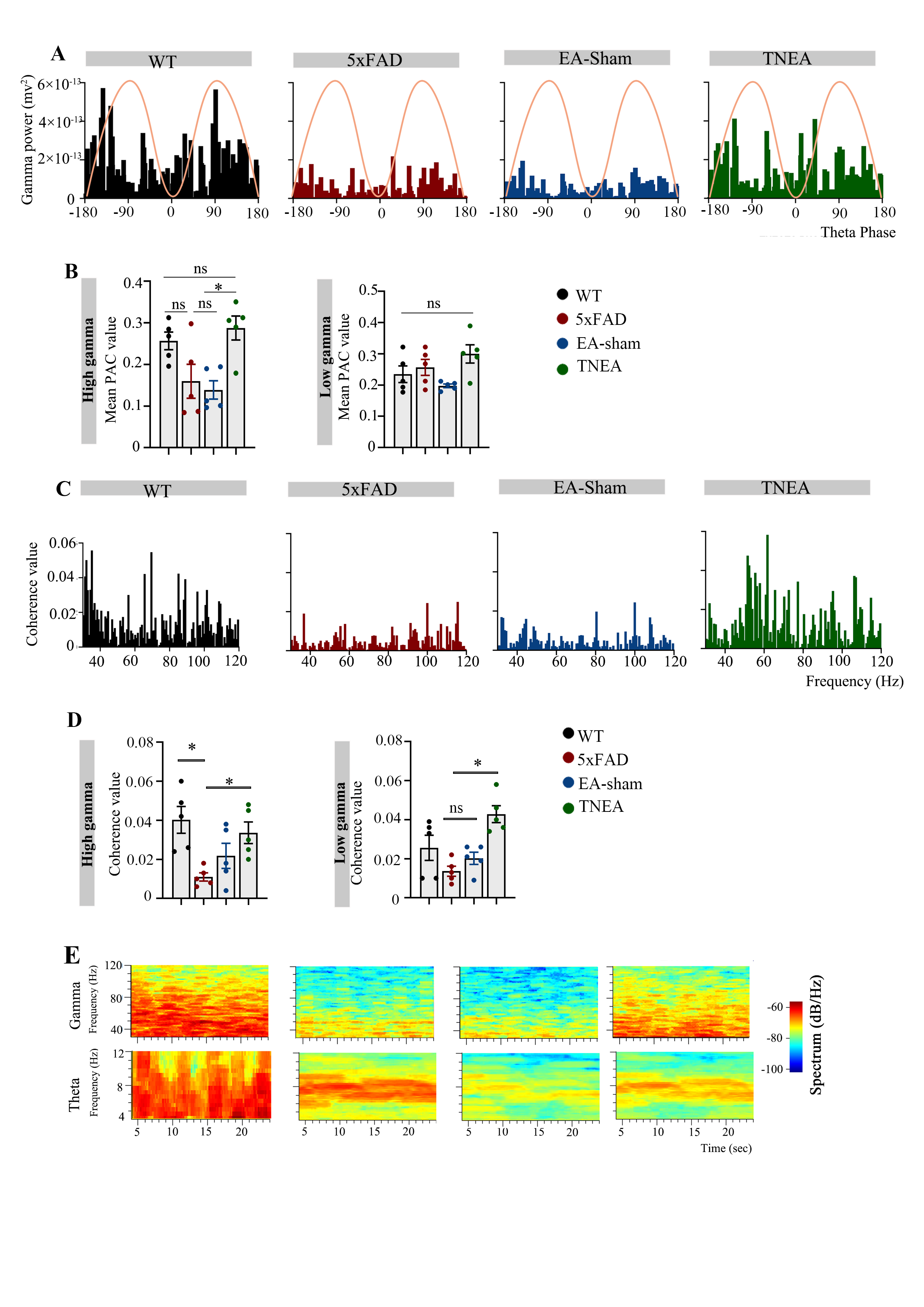

Supplement: Supplementary file 3 — Supplemental Figure 2 [file ADVS-13-e10885-s001.tif]

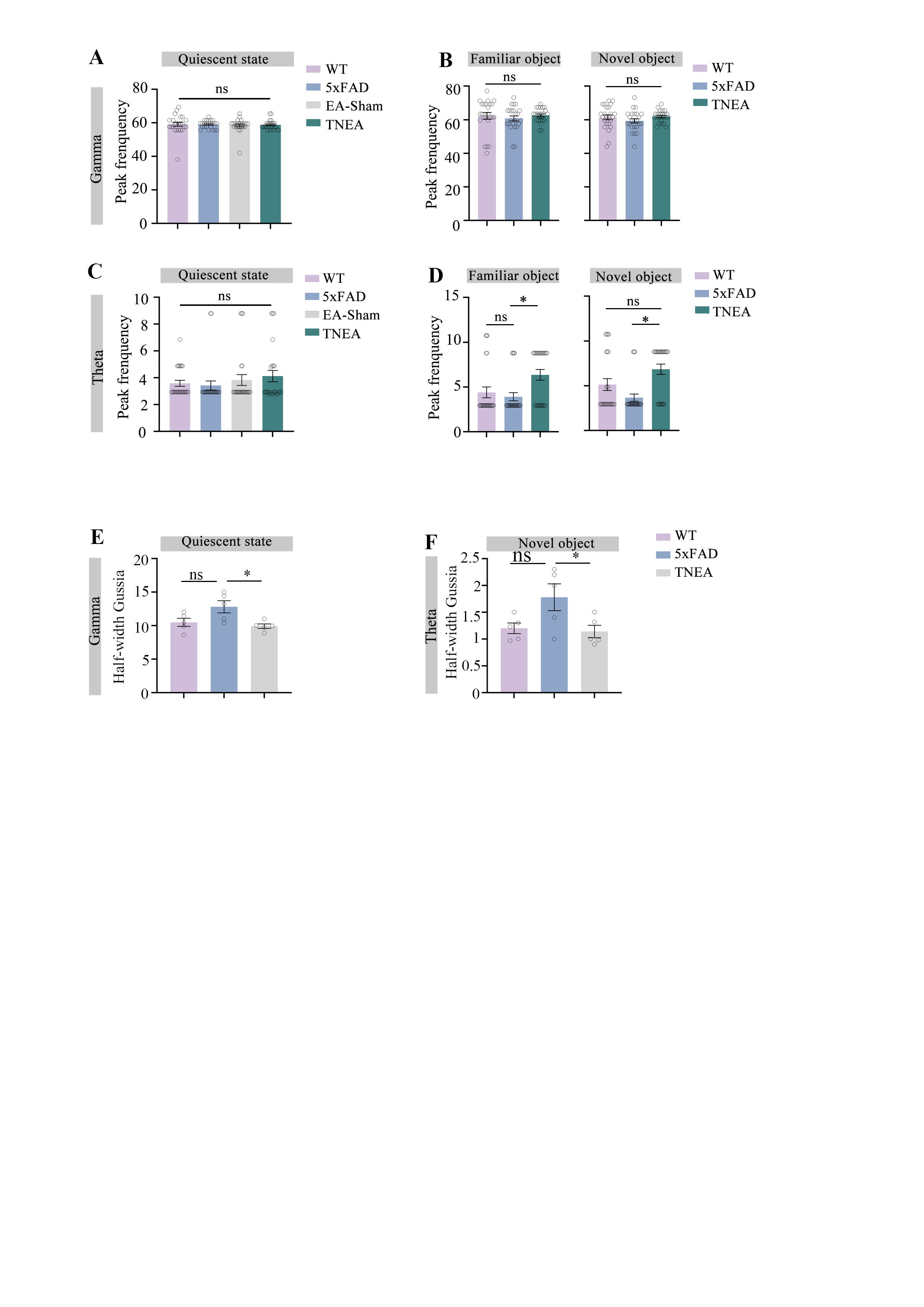

Supplement: Supplementary file 4 — Supplemental Figure 3 [file ADVS-13-e10885-s006.tif]

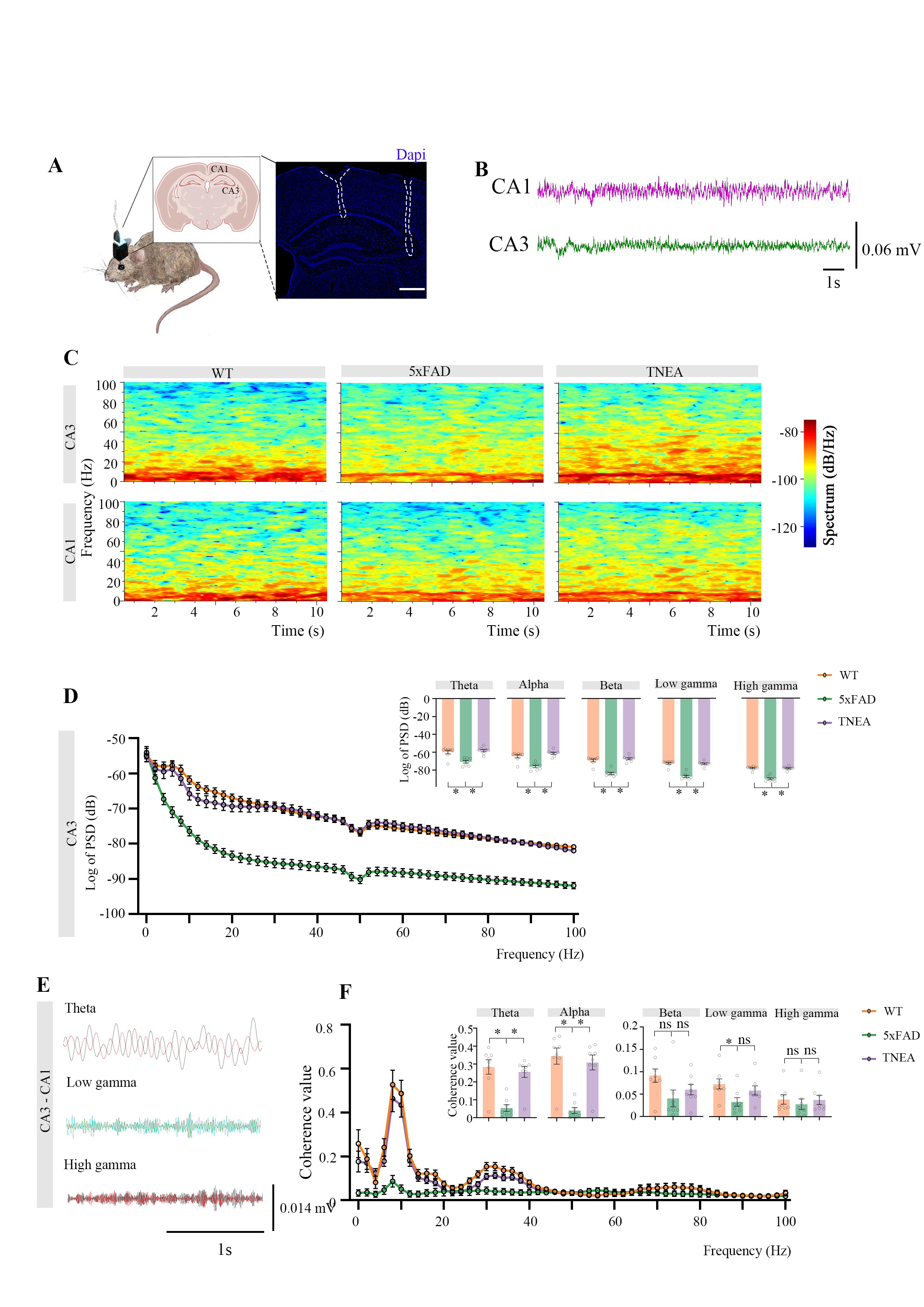

Supplement: Supplementary file 5 — Supplemental Figure 4 [file ADVS-13-e10885-s005.tif]

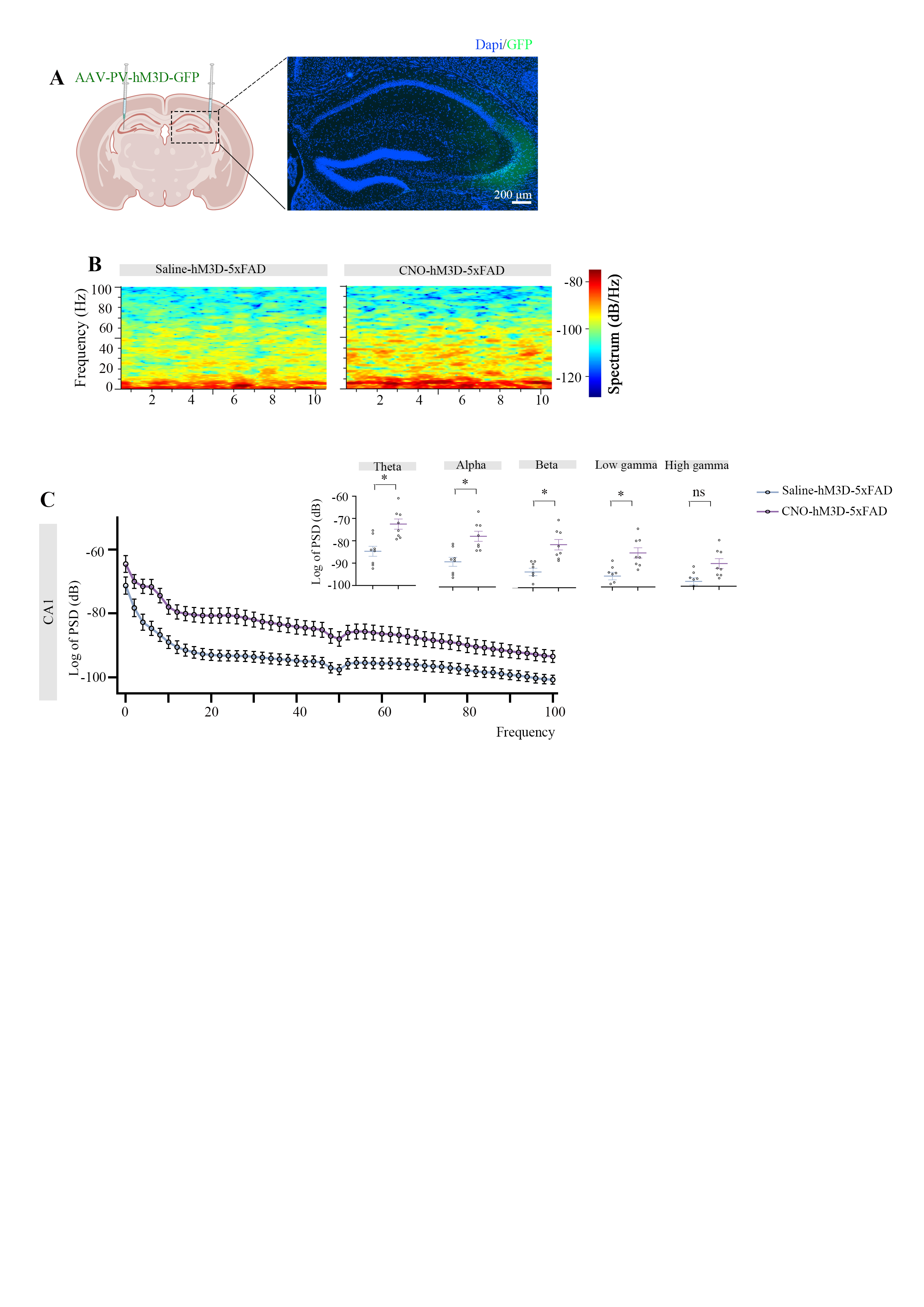

Supplement: Supplementary file 6 — Supplemental Figure 5 [file ADVS-13-e10885-s003.tif]

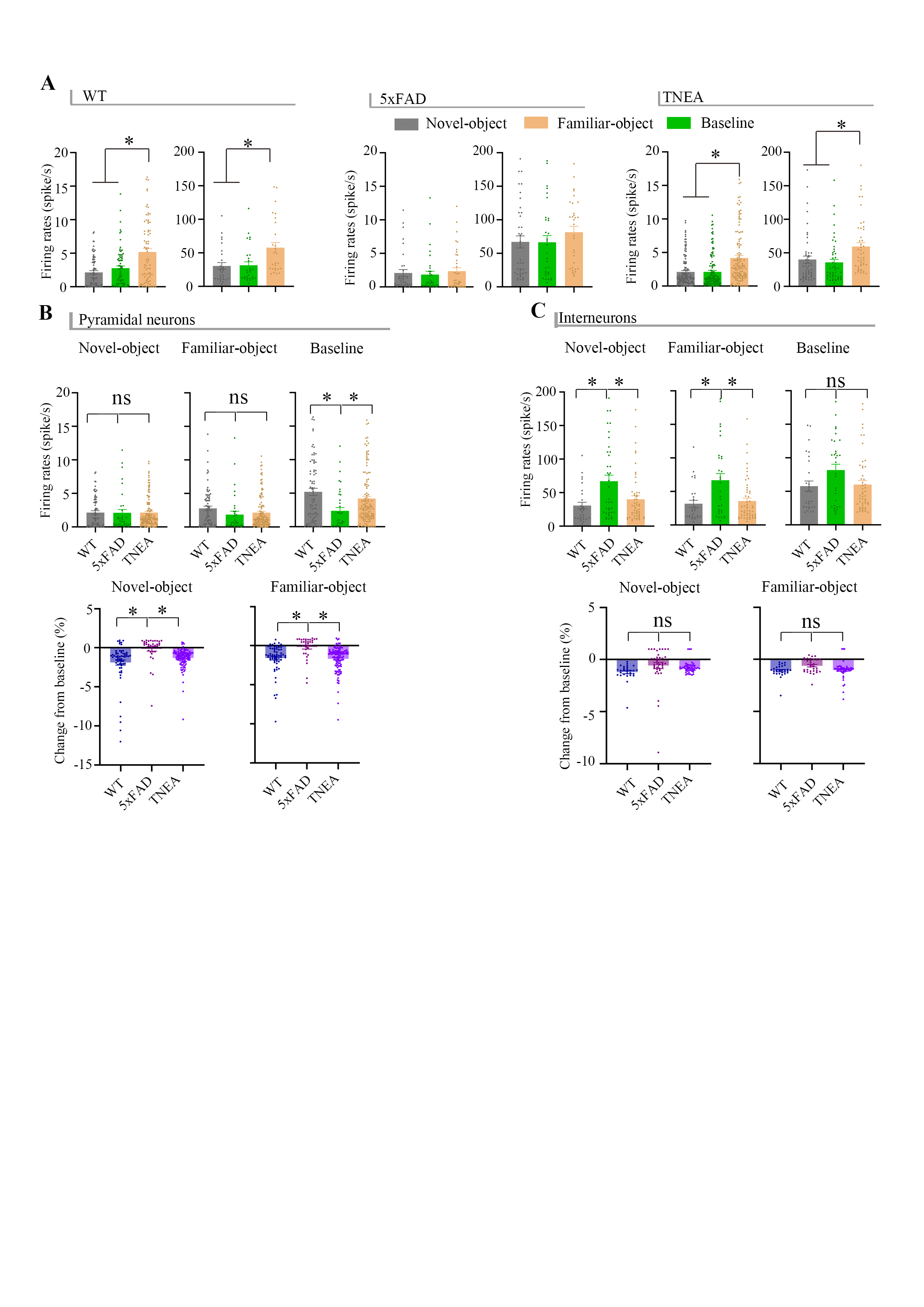

Supplement: Supplementary file 7 — Supplemental Figure 6 [file ADVS-13-e10885-s012.tif]

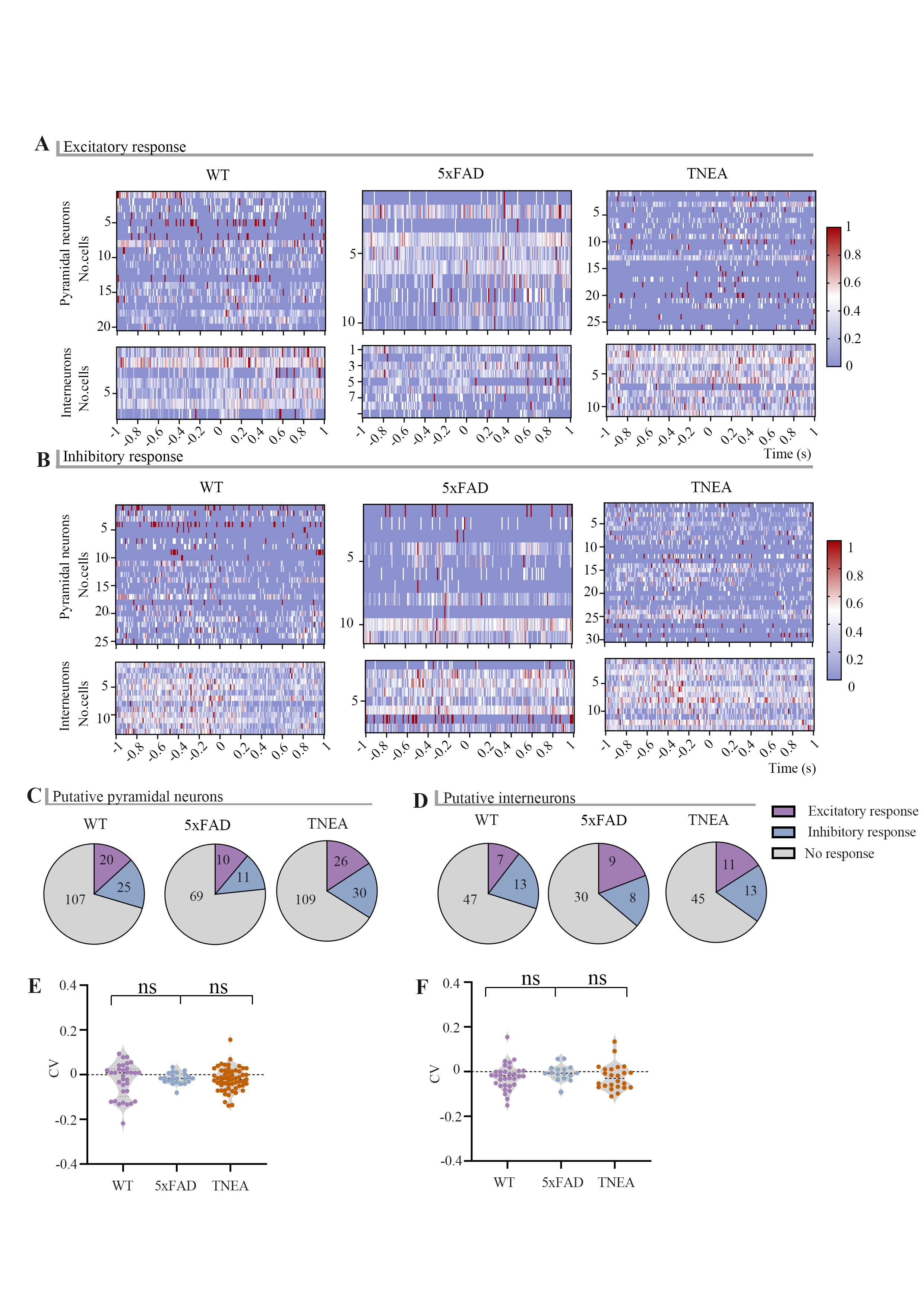

Supplement: Supplementary file 8 — Supplemental Figure 7 [file ADVS-13-e10885-s014.tif]

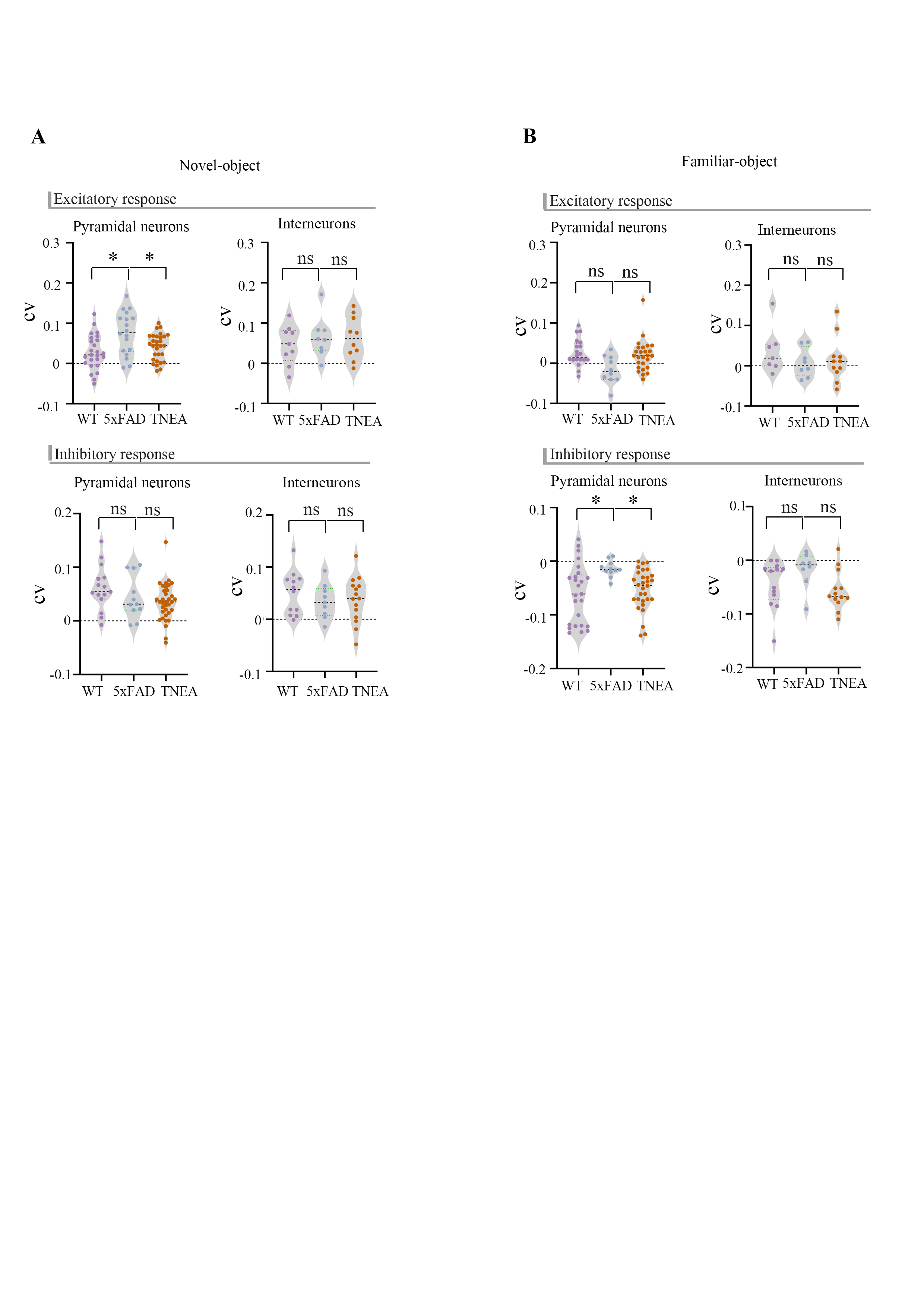

Supplement: Supplementary file 9 — Supplemental Figure 8 [file ADVS-13-e10885-s007.tif]

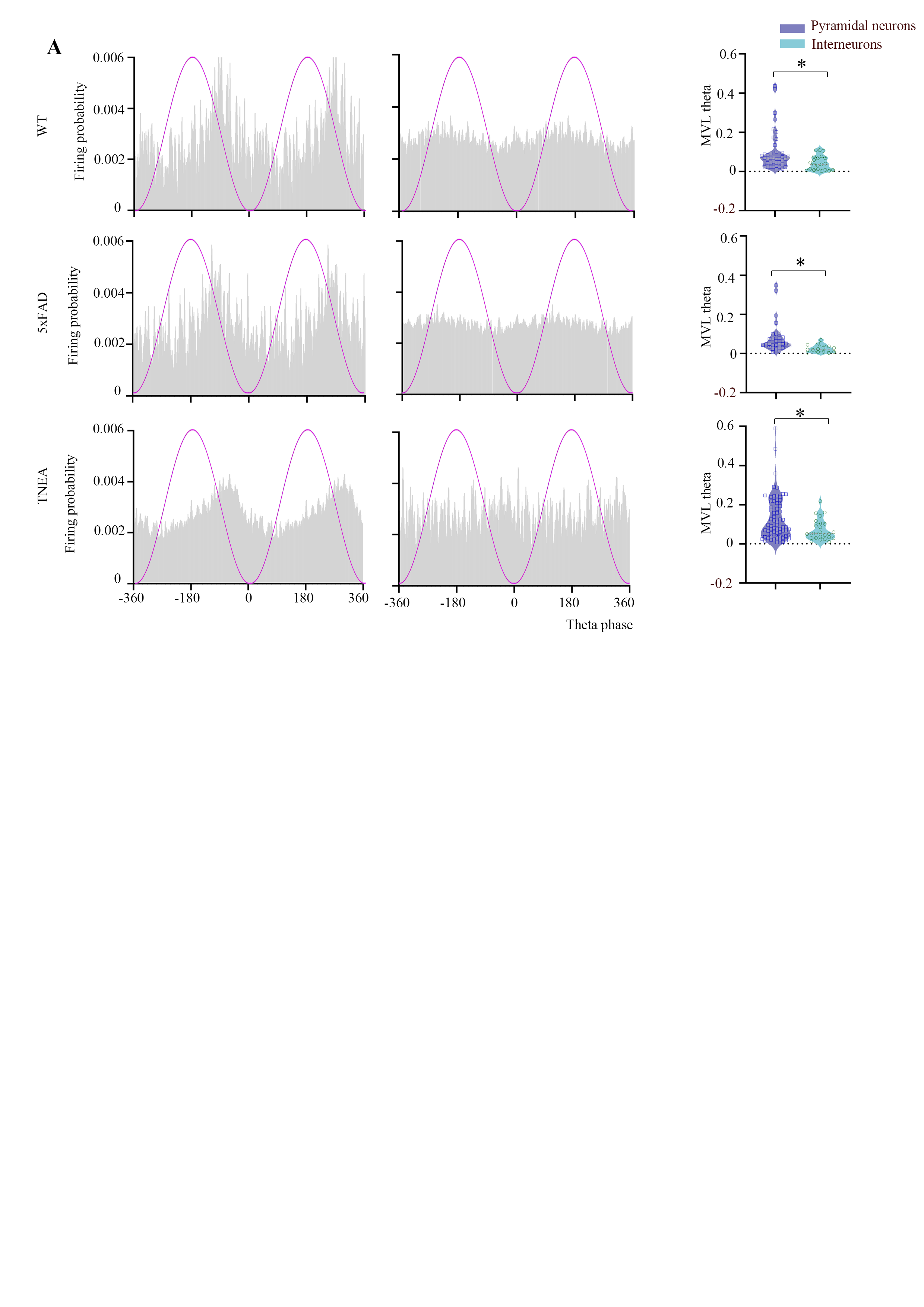

Supplement: Supplementary file 10 — Supplemental Figure 9 [file ADVS-13-e10885-s010.tif]

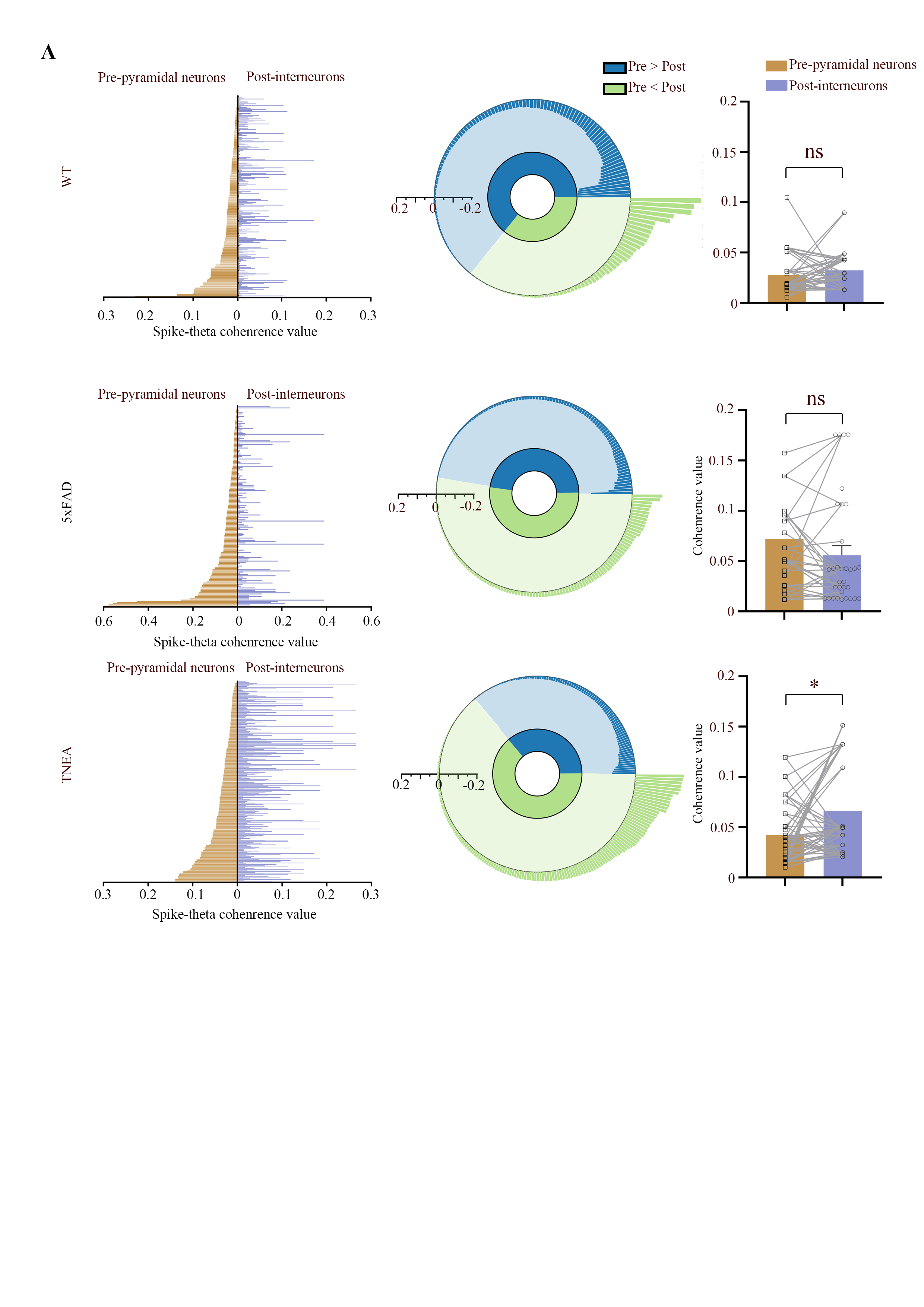

Supplement: Supplementary file 11 — Supplemental Figure 10 [file ADVS-13-e10885-s002.tif]

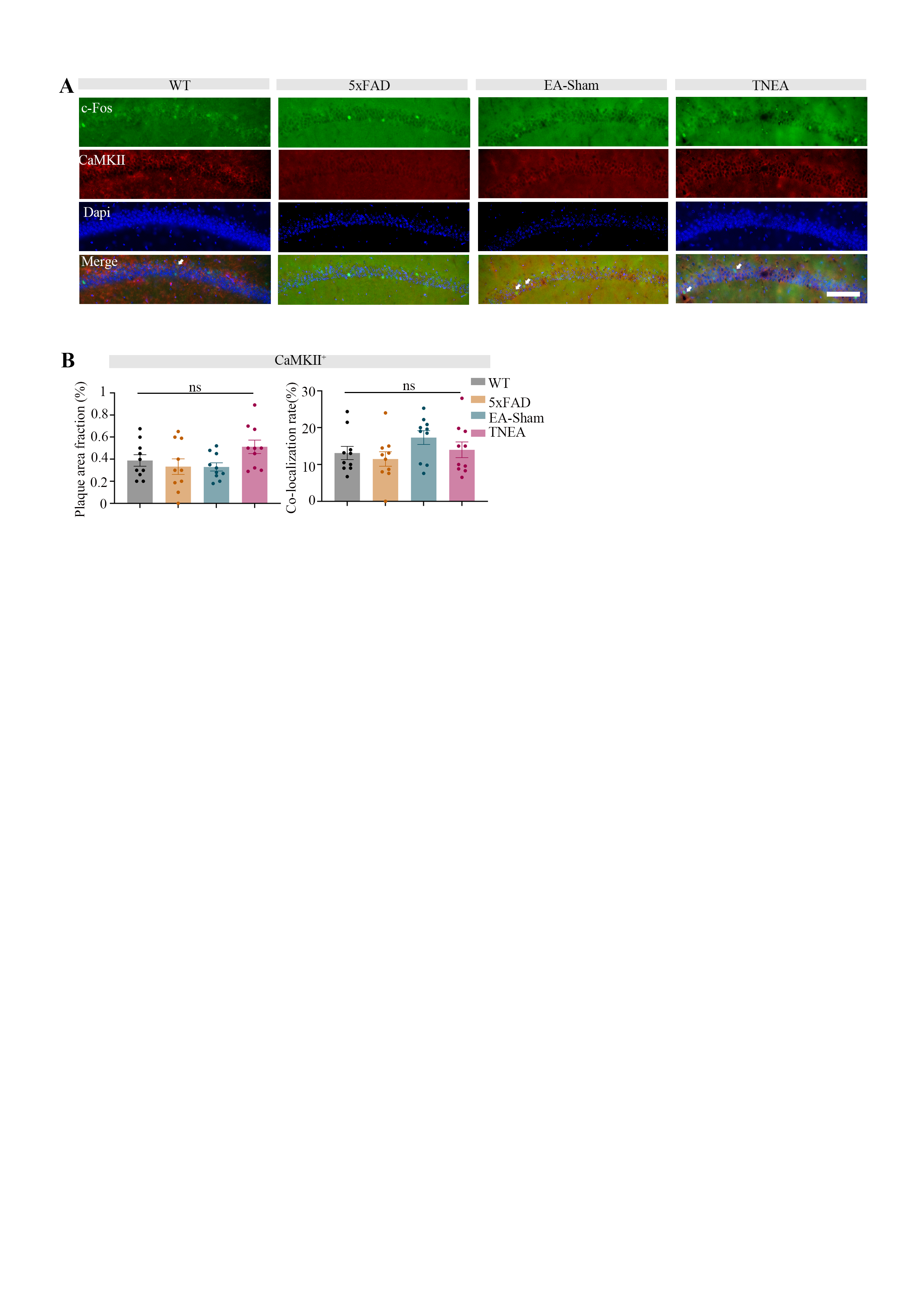

Supplement: Supplementary file 12 — Supplemental Figure 11 [file ADVS-13-e10885-s017.tif]

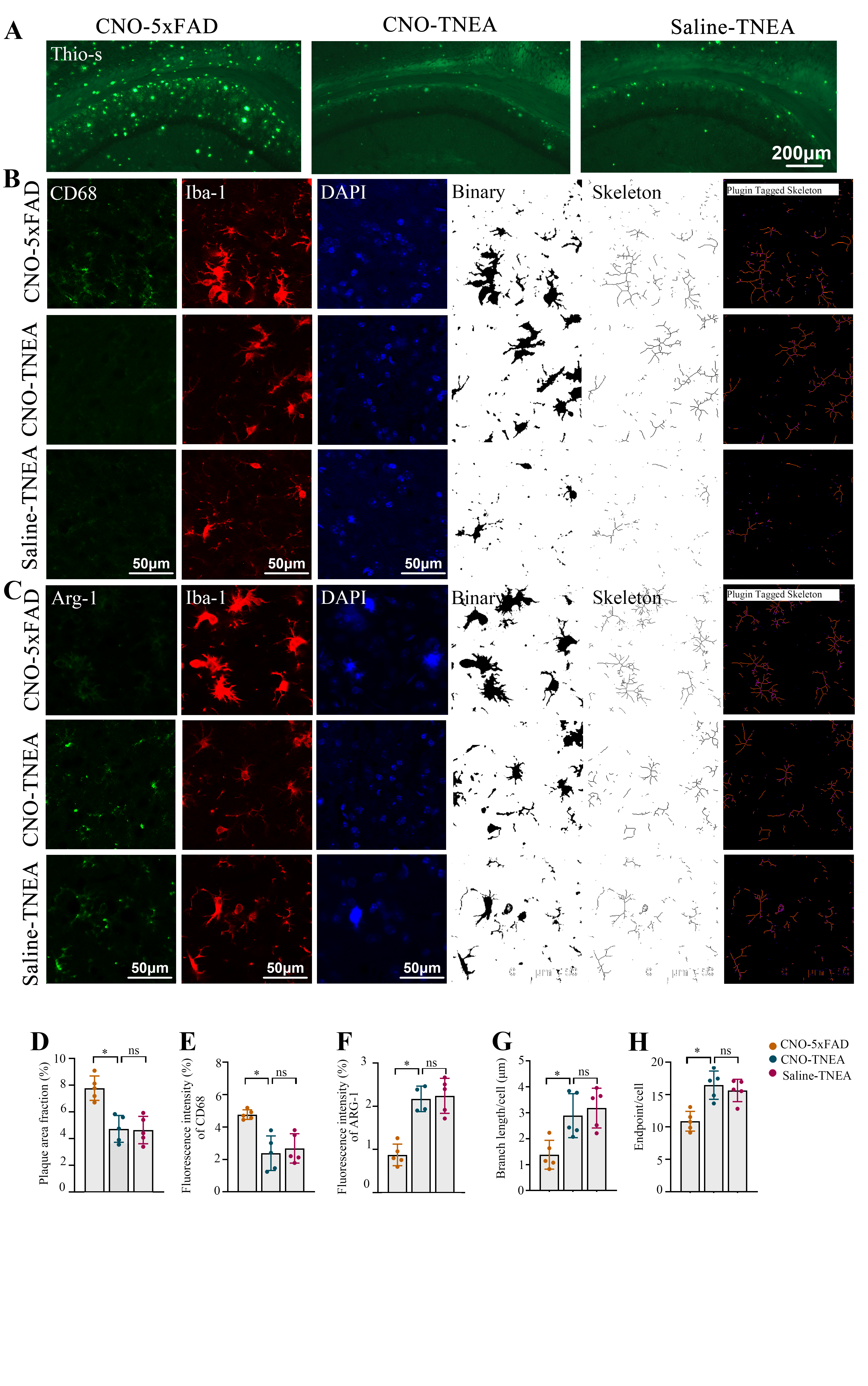

Supplement: Supplementary file 13 — Supplemental Figure 12 [file ADVS-13-e10885-s013.tif]

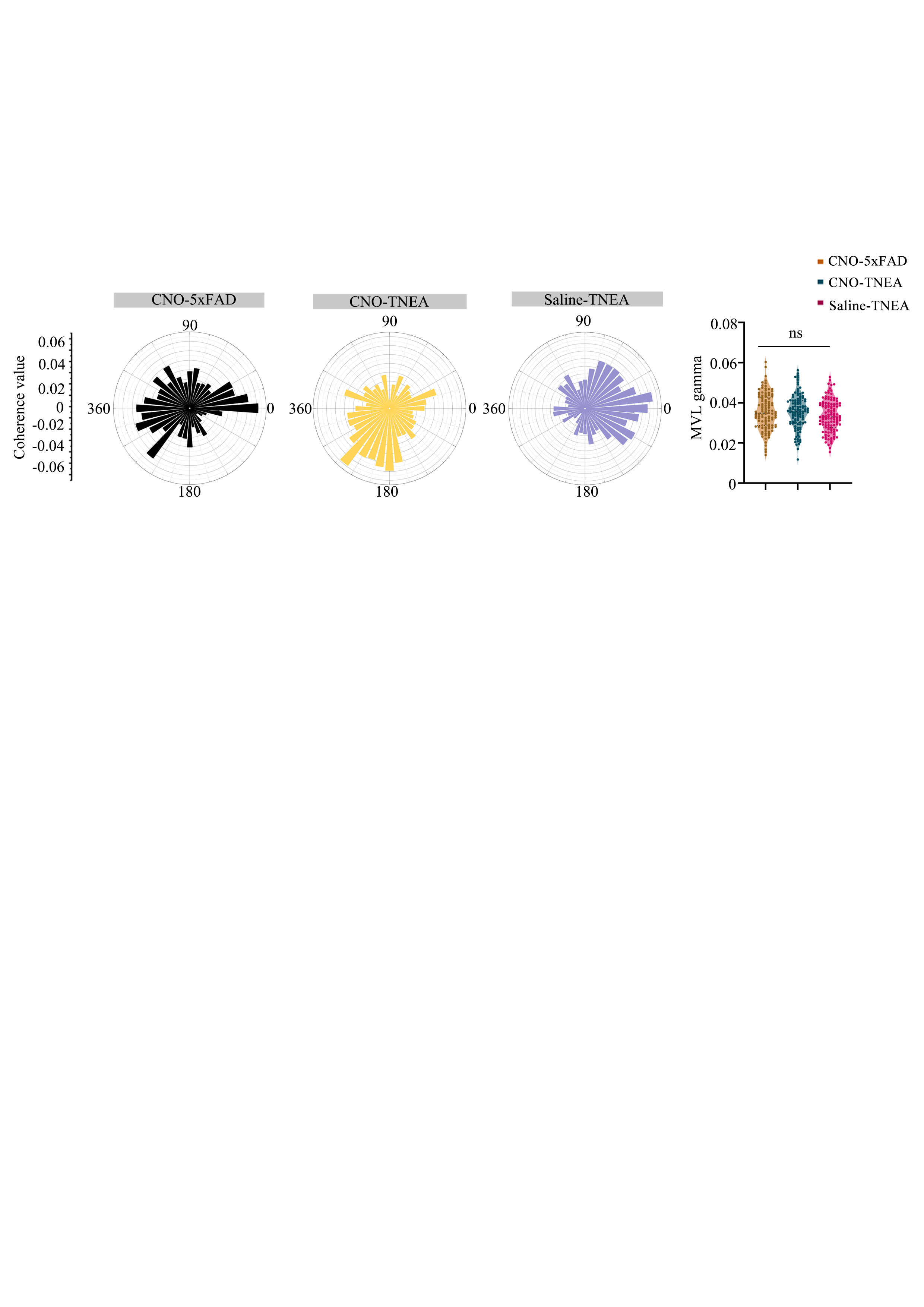

Supplement: Supplementary file 14 — Supplemental Figure 13 [file ADVS-13-e10885-s008.tif]

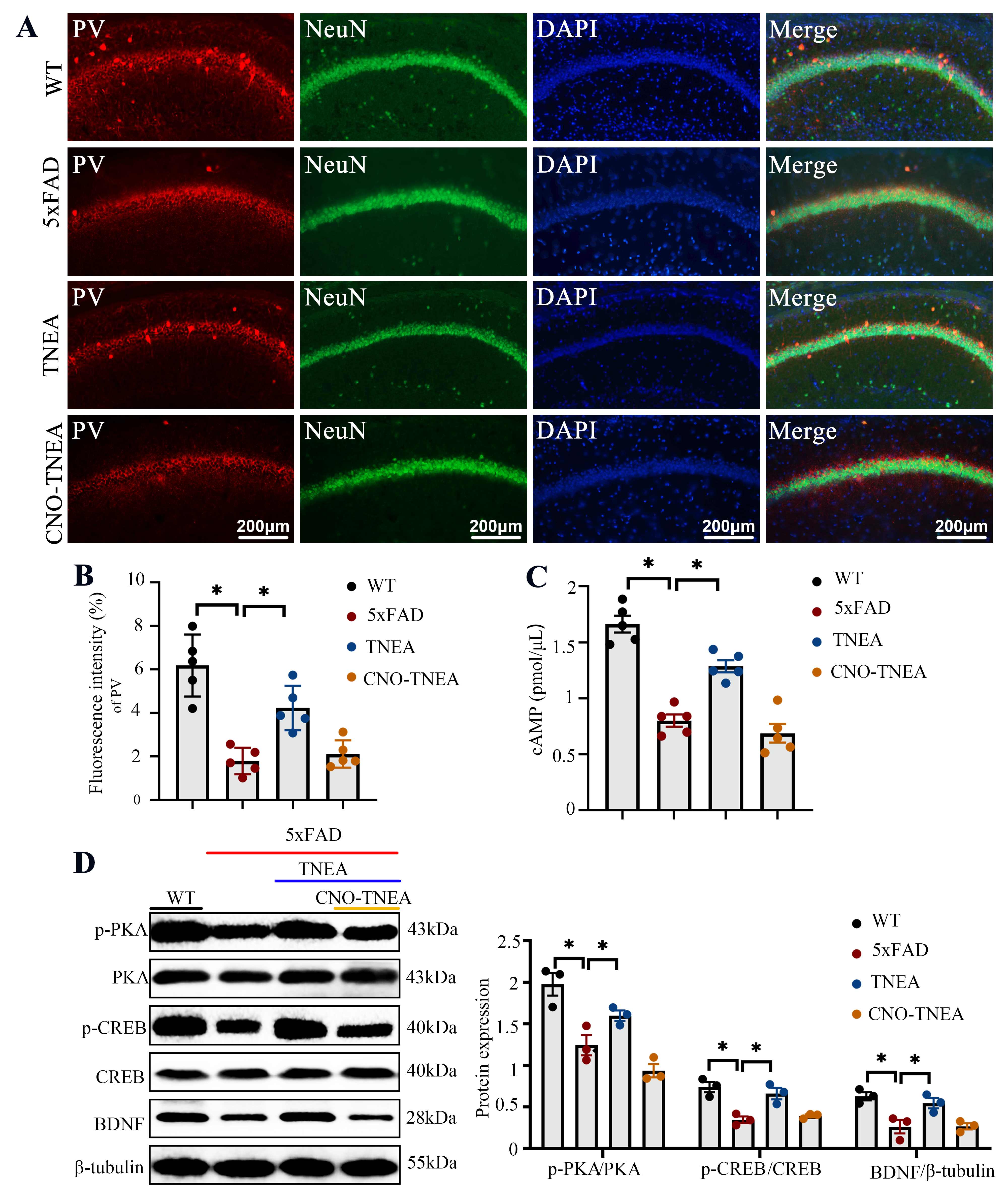

Supplement: Supplementary file 15 — Supplemental Figure 14 [file ADVS-13-e10885-s009.tif]

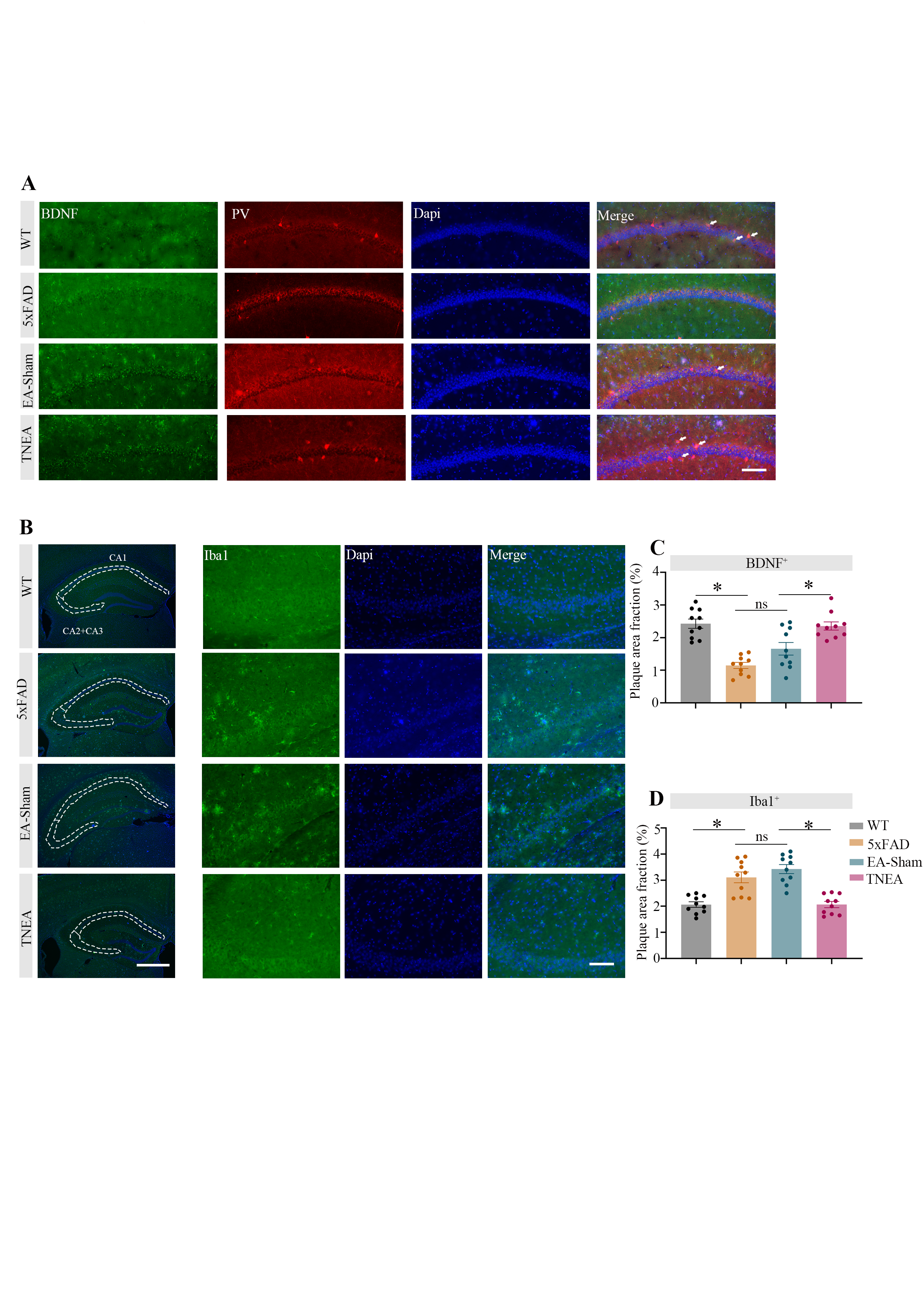

Supplement: Supplementary file 16 — Supplemental Figure 15 [file ADVS-13-e10885-s016.tif]

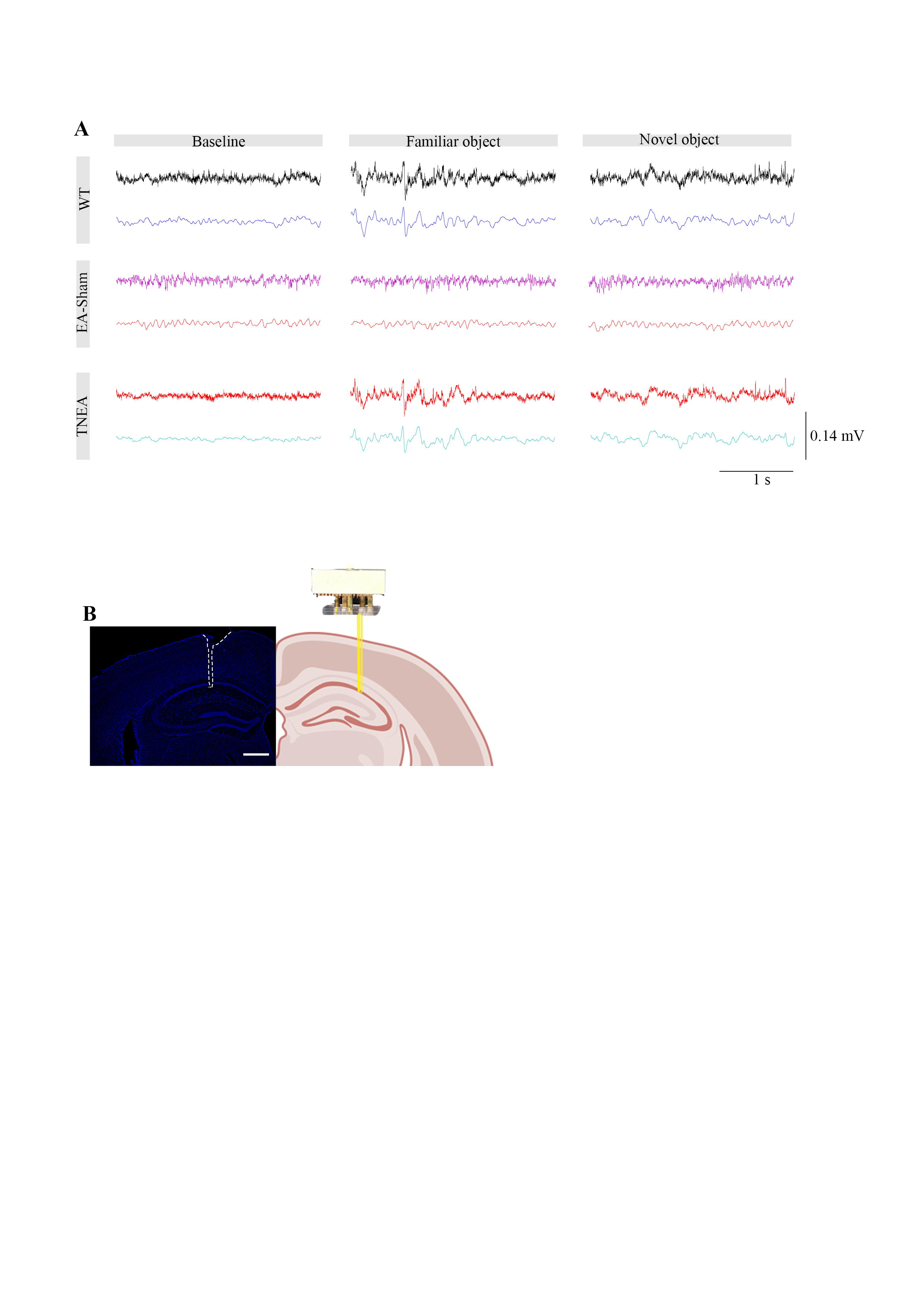

Supplement: Supplementary file 17 — Supplemental Figure 16 [file ADVS-13-e10885-s015.tif]
